# Synthesis, characterization, biological evaluation, and molecular modeling of novel nimesulide urea derivatives as potential MetAP2 inhibitors

**DOI:** 10.1038/s41598-026-50210-0

**Published:** 2026-04-24

**Authors:** Özgür Yılmaz, Yağmur Biliz, Elif Kuloğlu, Kübra Arancı, Ömer Erdoğan, Özge Çevik, Müfide Karahasanoğlu, Naz Mina Mert Şahin, Ayşe Buse Çakır, Bilge Tuzcu, Kemal Yelekçi, Ş. Güniz Küçükgüzel

**Affiliations:** 1 TUBITAK Marmara Research Center, 41470 Gebze, Kocaeli Türkiye; 2 Department of Pharmaceutical Chemistry, Faculty of Pharmacy, Istanbul University, 34116 Istanbul, Türkiye; 3 Department of Chemical Engineering, Faculty of Engineering and Natural Sciences, Istanbul Health and Technology University, 34275 Istanbul, Türkiye; 4 Department of Chemistry, Faculty of Art and Science, Bursa Uludağ University, 16059 Bursa, Türkiye; 5 Department of Bioengineering, Faculty of Engineering, Marmara University, 34854 Istanbul, Türkiye; 6 Center for Nanotechnology and Biomaterials Application and Research (NBUAM), Marmara University, Istanbul, Türkiye; 7 Department of Biochemistry, School of Medicine, Gaziantep Islam Science and Technology University, 27010 Gaziantep, Türkiye; 8 Department of Biochemistry, School of Medicine, Aydın Adnan Menderes University, 09010 Aydın, Türkiye; 9 Department of Molecular Biology and Genetics, Faculty of Engineering and Natural Sciences, Kadir Has University, 34083 Istanbul, Türkiye; 10 Department of Pharmaceutical Chemistry, Faculty of Pharmacy, Istanbul Kent University, Kagithane, Istanbul Türkiye; 11 Department of Pharmaceutical Chemistry, Faculty of Pharmacy, Fenerbahçe University, 34758 Atasehir, Istanbul Türkiye

**Keywords:** Nimesulide, Urea, MetAP2, Annexin-V-PI, Antioxidant, Biochemistry, Cancer, Chemical biology, Chemistry, Computational biology and bioinformatics, Drug discovery

## Abstract

A series of nimesulide-derived ureas were synthesized in high yields (69–91%) via a novel synthetic method under organotin catalysis. The structural characterization of the synthesized compounds was determined using a variety of spectroscopic methods, including ¹H NMR, ¹³C NMR, FTIR, and HRMS. Following initial screening, selected compounds were further evaluated using specific biological assays, including cytotoxicity analysis, apoptosis assessment by Annexin V/PI staining, analysis of Bax and Bcl-2 protein expression, and detection of DNA fragmentation using AO/EB staining. These evaluations were carried out in a variety of cell lines, including MDA-MB-231 (human triple-negative breast cancer cells), HeLa (human cervical cancer cells), PC-3 (androgen-independent human prostate cancer cells), MKN-45 (human gastric cancer cells), U87 (human glioblastoma cancer cells), and HUVEC (human umbilical vein endothelial cells). Three of the compounds demonstrated efficacy in MDA-MB-231 cells, resulting in increased AO/EB staining and annexin-V-PI binding levels, and increased Bax/Bcl-2 ratios. Numerous studies implicate MetAP2 in angiogenesis. MetAP2 stimulates cancer cell proliferation when it is upregulated and appears to play an essential role in tumor progression. In connection with the cell lines studied in this study, a crucial MetAP2 enzyme target was selected for in silico studies to support the experimental outcomes. The three promising compounds have been demonstrated to accelerate cell apoptosis and inhibit cell division by targeting MetAP2. The compounds synthesized in this study potential to overcome the challenges of targeted therapies in triple-negative breast cancer. Alongside these findings, the antioxidant potential of the synthesized compounds was evaluated using the 2,2-diphenyl-1-picrylhydrazyl (DPPH) radical scavenging assay. One of the compounds exhibited a radical scavenging activity comparable to the reference standard. In-silico molecular modeling studies were conducted in the final phase to evaluate the newly designed inhibitors as potential anticancer and antioxidant drug candidates. The most likely conformations of the MetAP2-ligand complex were sorted according to the free energy of binding score (kcal/mol) and agree with the experimental inhibition values. The computational results suggest that the newly synthesized compounds may be potential anticancer drug candidates.

**Supplementary Information:**

The online version contains supplementary material available at 10.1038/s41598-026-50210-0.

## Introduction

Nimesulide (N-(4-nitro-2-phenoxyphenyl)-methanesulfonamide) is a nonsteroidal anti-inflammatory drug (NSAID) with relatively low toxicity, widely prescribed for the management of headache, fever, vascular disorders, and inflammatory conditions^1–3^. Although long-term NSAID therapy is commonly associated with gastrointestinal adverse events, accumulating pharmacological evidence suggests that nimesulide is generally better tolerated and is linked to a lower incidence of gastrointestinal complications compared with conventional NSAIDs^4^.

In addition to the well-established association between inflammation and cancer, accumulating evidence indicates that chronic inflammation plays a significant role in tumor initiation and progression. Given the pro-tumorigenic role of COX-2, nimesulide has emerged as a promising anticancer candidate with demonstrated antiproliferative and pro-apoptotic effects^5–29^. Despite these promising biological activities, the clinical application of nimesulide is limited due to safety concerns, most notably dose- and duration-dependent hepatotoxicity^30–34^. Accordingly, structural modification and repurposing strategies support nimesulide as a promising scaffold for novel anticancer agents^8^.Various nimesulide analogs have been designed to enhance its anticancer activity, and their biological effects have been evaluated across different cancer cell lines. Representative derivatives have been reported to exhibit pronounced antiproliferative and pro-apoptotic effects in multiple cancer models, including hepatoma, prostate, breast, and colorectal cancers^35–38^. In addition, Güngör et al. synthesized novel amide/sulfonamide derivatives of nimesulide and demonstrated significant antiproliferative activity in cancer cell lines, with IC₅₀ values in the low micromolar range^31,39^.

Extensive chemical modification of the nimesulide scaffold has demonstrated that the anticancer activity of its derivatives cannot be fully explained by cyclooxygenase-2 (COX-2) inhibition alone, as nimesulide analogs frequently exhibit COX-2-independent mechanisms of action. In this context, increasing attention has been directed toward alternative molecular targets, among which methionine aminopeptidase-2 (MetAP2) has emerged as a particularly compelling candidate that can account for the COX-2-independent anticancer effects of nimesulide and its derivatives.

MetAP2 is an evolutionarily conserved, metal-dependent metalloprotease that catalyzes the removal of the initiator methionine residue from newly synthesized polypeptides, a process that is fundamental for protein maturation and cell viability.^40,41^ Beyond its essential role in protein biosynthesis, MetAP2 plays a critical role in endothelial cell growth and proliferation during tumor angiogenesis and is frequently overexpressed in multiple human cancers, where its upregulation has been associated with enhanced tumor cell proliferation and disease progression^42^. Importantly, pharmacological inhibition of MetAP2 has been shown to suppress cell growth and tumor progression, further supporting its functional involvement in cancer-related regulatory pathways and validating MetAP2 as a therapeutically relevant target in oncology^41,43^. Numerous studies have implicated MetAP2 in angiogenic signaling pathways, supporting its classification as a druggable molecular target and a key determinant of solid tumor progression^44–46^.

The therapeutic relevance of MetAP2 has been further supported by extensive efforts to develop MetAP2 inhibitors as anticancer agents. Potent inhibitors such as fumagillin, derived from Aspergillus fumigatus Fresenius, and its synthetic analogues TNP-470, PPI-2458, and CKD-732, which irreversibly target the MetAP2 enzyme, have demonstrated significant anti-angiogenic and antiproliferative effects in preclinical studies by disrupting endothelial cell cycle progression^42^. However, the clinical translation of these inhibitors has been limited, as the U.S. Food and Drug Administration (FDA) has deemed agents such as TNP-470 unacceptable at high doses due to reversible neurotoxic side effects, systemic toxicity, and low oral bioavailability^40,47^. To date, no MetAP2-targeting drug has been approved for clinical use, and the limitations associated with existing inhibitors underscore the need for safer, reversible, and more selective MetAP2-targeted therapeutic strategies^40^.

Given the overlap between the biological pathways regulated by MetAP2 particularly those governing cell proliferation, apoptosis, and angiogenesis and the anticancer activities reported for nimesulide derivatives, MetAP2 was selected in the present study as a rational molecular target for in silico investigations to support and mechanistically contextualize the experimental findings obtained in the relevant cancer cell lines.

The biological pathways modulated directly or indirectly by MetAP2 inhibitors, such as suppression of tumor cell proliferation, induction of apoptosis, and inhibition of angiogenesis, largely overlap with the anticancer activities reported in the literature for nimesulid and its derivatives^48–50^. The sulfonamide group and aromatic skeleton in the structure of nimesulide offer a favorable platform for structural modifications aimed at non-covalent interactions with the metal-dependent active site of MetAP2. Nimesulide and its derivatives are predicted to be promising candidates for the development of novel, selective MetAP2-targeted anticancer agents that can overcome the toxicity issues of existing inhibitors. Another important feature supporting the potential for this target is that nimesulide exhibits additional biological effects that can modulate cellular redox balance.

Beyond its role in MetAP2-related anticancer mechanisms, accumulating evidence indicates that nimesulide also exerts antioxidant effects that may contribute to its overall biological activity. In this context, researchers have focused on the free radical scavenging activity of nimesulide and its potential to reduce oxidative stress^51^. Maffei et al. investigated the antioxidant activity of nimesulide and its primary metabolites and reported that nimesulide was more effective than its metabolites in preventing hydroxyl radical-induced depolymerization of hyaluronic acid^52,53^.

In light of these findings, the present study aimed to synthesize novel nimesulide analogs with enhanced antiproliferative and antioxidant properties while minimizing the formation of nitro-reduction products responsible for hepatotoxicity. Accordingly, novel nimesulide urea derivatives were synthesized under organotin-catalyzed conditions and structurally characterized using spectroscopic techniques. In addition, molecular simulation studies were performed to explore the potential interactions of the synthesized compounds with the MetAP2 enzyme. Finally, the antioxidant activity, enzymatic effects, and cytotoxic properties of the compounds were evaluated across various cancer cell lines.

## Experimental

### Chemistry

All reagents and solvents utilized in the chemical syntheses and in vitro studies were obtained from commercial suppliers (Merck, Sigma-Aldrich, Acros Organics, and Thermo Fisher Scientific) and used without further purification. The synthesized compounds were purified by column chromatography using silica gel (particle size 0.063–0.200 mm). The progress of the reactions was monitored by thin-layer chromatography (TLC) (Hexane/ethyl acetate t = 25 °C) under ultraviolet (UV) light at 254 nm. Melting points were measured using a Buchi Stuart SMP20 melting point apparatus. ^1^H and^13^ C nuclear magnetic resonance (NMR) spectra (Varian Premium Shielded) were recorded at 600 and 150 MHz, respectively, with DMSO-*d*_*6*_ as the solvent and tetramethylsilane (Me_4_Si) as the internal standard. Fourier-transform infrared (FT-IR) spectra were collected on a PerkinElmer Spectrum BX FT-IR spectrometer (Massachusetts, USA). High-resolution mass spectrometry (HR-MS) analyses were performed using a Thermo Fisher Scientific Orbitrap Q Exactive mass spectrometer (2017, Serial No: 02820 L) equipped with an electrospray ionization (ESI) source.

### Procedure for the synthesis of N-(4-Amino-2-phenoxy-phenyl)-methanesulfonamide (2)^39^

Nimesulide (19.46 mmol) was dissolved in dry methanol (150 mL) under a nitrogen atmosphere. A solution of SnCl₂ (87.02 mmol) in concentrated HCl (30 mL) was prepared and added dropwise to the reaction mixture over a period of 10 min. The resulting mixture was then refluxed for 3h. Upon completion of the reaction, the solvent was removed under reduced pressure using a rotary evaporator. The resulting residue was extracted with ethyl acetate, and the organic phase was neutralized with 1 M NaOH solution. The mixture was subsequently washed with distilled water. The organic layer was dried over anhydrous sodium sulfate (Na_2_SO_4_), filtered, and concentrated under reduced pressure. The crude product was purified by recrystallization from ethanol.

*N*-(4-Amino-2-phenoxy-phenyl)-methanesulfonamide (2): White powder, Yield 68%, mp 166–168 °C, FT-IR υ_max_ (cm^− 1^): 3397 and 3330 (N-H), 3015 (Aromatic ring, =C–H), 2917 (Alkyl, C–H), 1583 and 1506 (Aryl, C=C), 1487, 1321 (C–S), 1211 and 1150 (C–N), 757. ^1^H NMR (600 MHz, *d*_*6*_-DMSO; δ, ppm): 2.86 (S–C**H**_3_, s, 3h), 5.26 (Ar–N**H**_2_, s, 2 H), 6.02 (Ar–C**H**, d, J = 2.4 Hz, 1H), 6.27 (Ar–C**H**, dd, J = 8.5, 2.4 Hz, 1H), 6.97 (Ar–C**H**, d, J = 8.5 Hz, 1H), 7.04 (2xAr–C**H**, d, J = 7.8 Hz, 2 H), 7.14 (Ar–C**H**, pt, J = 7.4 Hz, 1H), 7.40 (2xAr–C**H**, m, 2 H), 8.75 (Ar–N**H**–S, s, 1H). ^13^C NMR (126 MHz, d_6_-DMSO; δ, ppm): 40.0 **(C**H_3_), 102.8 **(**Ar–**C**), 108.8 **(**Ar–**C**), 114.8 **(**Ar–**C**), 119.3 **(**Ar–**C**), 123.5 **(**Ar–**C**), 129.9 **(**Ar–**C**), 130.5 **(**Ar–**C**), 149.1 **(**Ar–**C**), 153.1 **(**Ar–**C**), 156.2 **(**Ar–**C**). HRMS (ESI) m/z: calcd for C_13_H_14_N_2_SO_3_ [M + H]^+^ 279.07; found 279.08.

### Procedure for the synthesis of nimesulide ureas (3a-l)

Substituted isothiocyanates (1.5 mmol) and dibutyltin dilaurate (1.5 mmol) were added to a solution of compound **2** (1 mmol) in tetrahydrofuran (5 mL). The reaction mixture was stirred under reflux for 12–18 h under a nitrogen atmosphere. The progress of the reaction was monitored by thin-layer chromatography (TLC) using hexane/ethyl acetate (7:3) as the eluent. Upon completion, the solvent was removed under reduced pressure, and the crude product was purified by column chromatography.

*N*-{4-[3-(2-Chloro-ethyl)-ureido]-2-phenoxy-phenyl}-methanesulfonamide (3a): White powder, Yield 88%, mp 167 °C, FT-IR υ_max_ (cm^−1^): 3341 (N–H), 3056 (Aromatic ring, =C–H), 2928 and 2853 (Alkyl, C–H), 1650 (C=O), 1586 and 1555 (Aryl, C=C), 1502, 1323 (C–S), 1212 (C–O), 1157 (C–N), 758. ^1^H NMR (600 MHz, DMSO-*d*_*6*_; δ, ppm): 2.91 (S–C**H**_3_, s, 3h), 3.29–3.33 (C**H**_2_, m, 2 H), 3.56–3.60 (C**H**_2_, m, 2 H), 6.35 (Ar–N**H**–CO, t, J = 5.8 Hz, 1H), 7.02 (Ar–C**H**, dd, J = 8.7, 2.3hz, 1H), 7.05 (2xAr–C**H**, d, J = 7.9 Hz 2 H), 7.12 (Ar–C**H**, d, J = 2.2 Hz, 1H), 7.17 (Ar–C**H**, pt, J = 7.4 Hz, 1H), 7.22 (Ar–C**H**, d, J = 8.7 Hz, 1H), 7.41 (2xAr–C**H**, pt, J = 7.9 Hz, 2 H), 8.83 (Ar–N**H**–S, s, 1H), 9.09 (Ar–N**H**–CO, s, 1H). Two sets of signals were observed in the NMR spectra; the second isomer is marked with an asterisk (*). ^13^C NMR (150 MHz, DMSO-*d*_*6*_; δ, ppm): 40.3 **(**S–**C**H_3_), 41.1 **(C**H_2_), 41.4 **(C**H_2_)*, 44.3 **(C**H_2_), 44.5 **(C**H_2_)*, 107.3 **(**Ar–**C**), 112.6 **(**Ar–**C**), 119.1 **(**Ar–**C**), 120.6 **(**Ar–**C**), 123.7 **(**Ar–**C**), 128.6 **(**Ar–**C**), 129.9 **(**Ar–**C**), 139.7 **(**Ar–**C**), 151.5 **(**Ar–**C**), 151.5 **(**Ar–**C**), 154.8 **(**Ar–**C**), 156.0 **(**Ar–**C**), 157.5 **(**NH–**C** **= O**), HRMS (ESI) m/z: calcd for C_16_H_18_N_3_SO_4_Cl [M + H]^+^ 384.07; found 384.08.

*N*-[4-(3-Ethyl-ureido)-2-phenoxy-phenyl]-methanesulfonamide (3b): White powder, Yield 90%, mp 220 °C, FT-IR υ_max_ (cm^−1^): 3311 (N–H), 3022 (Aromatic ring, =C–H), 2969, 2930 and 2865 (Alkyl, C–H), 1647 (C=O), 1589 and 1563 (Aryl, C=C), 1486, 1339 (C–S), 1211 (C–O), 1160 (C–N), 973, 763. ^1^H NMR (600 MHz, DMSO-*d*_*6*_; δ, ppm): 0.95–1.00 (C**H**_3_, m, 3h), 2.91 (S–C**H**_3_, s, 3h), 2.96–3.05 (CH_3_–C**H**_2_, m, 2 H), 6.01 (CH_2_–N**H**–CO, t, J = 5.5 Hz, 1H), 7.00 (Ar–C**H**, dd, J = 8.7, 2.1 Hz, 1H), 7.05 (2xAr–C**H**, d, J = 8.0 Hz 2 H), 7.12 (Ar–C**H**, d, J = 2.1 Hz, 1H), 7.16 (Ar–C**H**, pt, J = 7.4 Hz, 1H), 7.21 (Ar–C**H**, pt, J = 8.7 Hz, 1H), 7.41 (2xAr–C**H**, pt, J = 7.9 Hz, 2 H), 8.54 (Ar–N**H**–S, s, 1H), 9.08 (Ar–N**H**–CO, s, 1H). Two sets of signals were observed in the NMR spectra; the second isomer is marked with an asterisk (*). ^13^C NMR (150 MHz, DMSO-*d*_*6*_; δ, ppm): 15.4 **(C**H_3_), 15.8 **(C**H_3_)*, 33.9 **(C**H_2_), 34.0 **(C**H_2_)*, 40.2 **(**S–**C**H_3_), 107.2 **(**Ar–**C**), 112.5 **(**Ar–**C**), 119.1 **(**Ar–**C**), 120.3 **(**Ar–**CF**_**3**_), 123.7 **(**Ar–**C**), 128.6 **(**Ar–**C**), 129.9 **(**Ar–**C**), 140.1 **(**Ar–**C**), 151.5 **(**Ar–**C**), 154.8 **(**Ar–**C**) 156.1 **(**NH–**C** **= O**). HRMS (ESI) m/z: calcd for C_16_H_19_N_3_SO_4_ [M + H]^+^ 350.11; found 350.12.

*N*-[2-Phenoxy-4-(3-propyl-ureido)-phenyl]-methanesulfonamide (3c): White powder, Yield 87%, mp 174 °C, FT-IR υ_max_ (cm^−1^): 3323 and 3262 (N–H), 3063 (Aromatic ring, =C–H), 2960, 2932 and 2862 (Alkyl, C–H), 1646 (C=O), 1559 and 1506 (Aryl, C=C), 1489, 1325 (C–S), 1216 (C–O), 1146 (C–N), 960, 692, ^1^H NMR (600 MHz, DMSO-*d*_*6*_; δ, ppm): 0.83 (C**H**_3_, t, J = 7.4 Hz, 3h), 1.36–1.42 (CH_3_–C**H**_2_–CH_2_, m, 2 H), 2.92 (S–C**H**_3_, s, 3h), 2.96–2.99 (CH_3_–CH_2_–C**H**_2_, dd, J = 13.0, 6.7 Hz, 2 H), 6.05 (CH_2_–N**H**–CO, t, J = 5.7 Hz, 1H), 7.00–7.02 (Ar–C**H**, dd, J = 8.7, 2.3hz, 1H), 7.06 (2xAr–C**H**, d, J = 7.8 Hz 2 H), 7.12 (Ar–C**H**, d, J = 2.3hz, 1H), 7.17 (Ar–C**H**, pt, J = 7.4 Hz, 1H), 7.22 (Ar–C**H**, d, J = 8.7 Hz, 1H), 7.42 (2xAr–C**H**, dd, J = 8.3, 7.6 Hz, 2 H), 8.53 (Ar–N**H**–S, s, 1H), 9.08 (Ar–N**H**–CO, s, 1H).^13^C NMR (150 MHz, DMSO-*d*_*6*_; δ, ppm): 11.2 **(C**H_3_), 22.9 **(C**H_2_), 40.2 **(**S**–C**H_3_), 40.8 **(C**H_2_), 107.2 **(**Ar–**C**), 112.4 **(**Ar–**C**), 119.1 **(**Ar–**C**), 120.3 **(**Ar–**CF**_**3**_), 123.7 **(**Ar–**C**), 128.6 **(**Ar–**C**), 129.9 **(**Ar–**C**), 140.1 **(**Ar–**C**), 151.5 **(**Ar–**C**), 154.9 **(**Ar–**C**), 156.1 **(**NH–**C** **= O**). HRMS (ESI) m/z: calcd for C_17_H_21_N_3_SO_4_ [M + H]^+^ 364.13; found 364.13.

*N*-{4-[3-(2,4-Dichloro-phenyl)-ureido]-2-phenoxy-phenyl}-methanesulfonamide (3d): White powder, Yield 79%, mp 223 °C, FT-IR υ_max_ (cm^−1^): 3323 (N–H), 3070 (Aromatic ring, =C–H), 2961 (Alkyl, C–H), 1644 (C=O), 1584 and 1526 (Aryl, C=C), 1489, 1336 (C–S), 1214 (C–O), 1157 (C–N), 789. ^1^H NMR (600 MHz, DMSO-*d*_*6*_; δ, ppm): 2.95 (S–C**H**_3_, s, 3h), 7.09–7.11 (4xAr–C**H**, m, 4 H), 7.19 (Ar–C**H**, pt, J = 7.4 Hz, 1H), 7.30–7.35 (2xAr–C**H**, m, 2 H), 7.43 (2xAr–C**H**, pt, J = 7.9 Hz, 2 H), 7.60 (Ar–C**H**, d, J = 2.4 Hz, 1H), 8.11 (Ar–C**H**, d, J = 9.0 Hz, 1H), 8.29 (Ar–N**H**–CO, s, 1H), 9.22 (Ar–N**H**–S, s, 1H), 9.53 (Ar–N**H**–CO, s, 1H). ^13^C NMR (150 MHz, DMSO-*d*_*6*_; δ, ppm): 40.3 **(**S–**C**H_3_), 107.5 **(**Ar–**C**), 112.9 **(**Ar–**C**), 119.4 **(**Ar–**C**), 121.5 **(**Ar–**C**), 122.1 **(**Ar–**C**), 122.6 **(**Ar–**C**), 124.0 **(**Ar–**C**), 126.2 **(**Ar–**C**), 127.6 **(**Ar–**C**), 128.5 **(**Ar–**C**), 128.6 **(**Ar–**C**), 130.1 **(**Ar–**C**), 134.9 **(**Ar–**C**), 138.5 **(**Ar–**C**), 151.6 **(**Ar–**C**), 151.7 **(**Ar–**C**), 155.8 **(**NH–**C** **= O**), HRMS (ESI) m/z: calcd for C_20_H_17_N_3_SO_4_Cl_2_ [M + H]^+^ 466.03; found 466.04.

*N*-{4-[3-(3-Fluoro-phenyl)-ureido]-2-phenoxy-phenyl}-methanesulfonamide (3e): White powder, Yield 68%, mp 195 °C, FT-IR υ_max_ (cm^−1^): 3330 and 3263 (N–H), 3089 (Aromatic ring, =C–H), 2933 (Alkyl, C–H), 1656 (C=O), 1608 and 1548 (Aryl, C=C), 1488, 1324 (C–S), 1218 (C–O), 1141 (C–N), 746. ^1^H NMR (600 MHz, DMSO-*d*_*6*_; δ, ppm): 2.94 (S–C**H**_3_, s, 3h), 6.75–6.78 (Ar–C**H**, m, 1H), 7.06–7.11 (4xAr–C**H**, m, 4 H), 7.14 (Ar–C**H**, d, J = 2.0 Hz, 1H), 7.18 (Ar–C**H**, pt, J = 7.4 Hz, 1H), 7.25–7.30 (2xAr–C**H**, m, 2 H), 7.38–7.44 (3xAr–C**H**, m, 3h), 8.80 (Ar–N**H**–CO, s, 1H), 8.86 (Ar–N**H**–S, s, 1H), 9.19 (Ar–N**H**–CO, s, 1H). ^13^C NMR (150 MHz, DMSO-*d*_*6*_; δ, ppm): 40.3 **(**S–**C**H_3_), 104.8 **(**Ar–**C**), 105.0 **(**Ar–**C**), 107.9 **(**Ar–**C**), 108.2 **(**Ar–**C**), 108.3 **(**Ar–**C**), 113.2 **(**Ar–**C**), 114.0 **(**Ar–**C**), 119.1 **(**Ar–**C**), 121.5 **(**Ar–**C**), 123.9 **(**Ar–**C**), 128.5 **(**Ar–**C**), 130.0 **(**Ar–**C**), 130.3 **(**Ar–**C**), 130.3 **(**Ar–**C**), 138.7 **(**Ar–**C**), 141.3 **(**Ar–**C**), 151.4 **(**Ar–**C**), 152.1 **(**Ar–**C**), 155.9 **(**Ar–**C**), 161.5 **(**Ar–**C**), 163.1 **(**NH–**C** **= O**). HRMS (ESI) m/z: calcd for C_20_H_18_N_3_SFO_4_ [M + H]^+^ 416.10; found 416.11.

*N*-{4-[3-(4-Fluoro-phenyl)-ureido]-2-phenoxy-phenyl}-methanesulfonamide (3f): White powder, Yield 71%, mp 227 °C, FT-IR υ_max_ (cm^−1^): 3318 and 3256 (N–H), 3065 (Aromatic ring, =C–H), 2930 (Alkyl, C–H), 1721 (C=O), 1611 and 1555 (Aryl, C=C), 1502, 1398 (C–S), 1216 (C–O), 1143 (C–N), 692. ^1^H NMR (600 MHz, DMSO-*d*_*6*_; δ, ppm): 2.94 (S–C**H**_3_, s, 3h), 7.07–7.10 (5xAr–C**H**, m, 5 H), 7.15 (Ar–C**H**, d, J = 2.3hz, 1H), 7.18 (Ar–C**H**, pt, J = 7.4 Hz 1H), 7.28 (Ar–C**H**, d, J = 8.7 Hz, 1H), 7.37–7.40 (2xAr–C**H**, m, 2 H), 7.41–7.44 (2xAr–C**H**, m, 2 H), 8.59 (Ar–N**H**–CO, s, 1H), 8.79 (Ar–N**H**–S, s, 1H), 9.17 (Ar–N**H**–CO, s, 1H). ^13^C NMR (150 MHz, DMSO-*d*_*6*_; δ, ppm): 40.3 **(**S–**C**H_3_), 107.8 **(**Ar–**C**), 113.1 **(**Ar–**C**), 115.2 **(**Ar–**C**), 115.3 **(**Ar–**C**), 119.2 **(**Ar–**C**), 120.1 **(**Ar–**C**), 120.2 **(**Ar–**C**), 121.2 **(**Ar–**C**), 123.9 **(**Ar–**C**), 128.6 **(**Ar–**C**), 130.0 **(**Ar–**C**), 135.7 **(**Ar–**C**), 139.0 **(**Ar–**C**), 151.5 **(**Ar–**C**), 152.4 **(**Ar–**C**), 155.9 **(**Ar–**C**), 156.6 **(**Ar–**C**),158.2 **(**NH–**C** **= O**). HRMS (ESI)m/z: calcd for C_20_H_18_N_3_SFO_4_ [M + H]^+^ 416.10; found 416.11.

*N*-{4-[3-(2-Chloro-phenyl)-ureido]-2-phenoxy-phenyl}-methanesulfonamide (3g): White powder, Yield 81%, mp 173 °C, FT-IR υ_max_ (cm^−1^): 3265 (N–H), 3068 (Aromatic ring, =C–H), 2926 (Alkyl, C–H), 1659 (C=O), 1591 and 1546 (Aryl, C=C), 1486, 1329 (C–S), 1216 (C–O), 1144 (C–N), 691.^1^H NMR (600 MHz, DMSO-*d*_*6*_; δ, ppm): 3.01 (S–C**H**_3_, s, 3h), 7.05–7.09 (Ar–C**H**, m, 1 H), 7.15–7.19 (4xAr–C**H**, m, 4 H), 7.25 (Ar–C**H**, pt, J = 7.4 Hz 1 H), 7.29–7.34 (Ar–C**H**, m, 1 H), 7.36–7.38 (Ar–C**H**, m, 1 H), 7.47–7.51 (3xAr–C**H**, m, 3h), 8.11–8.14 (Ar–C**H**, dd, J = 8.3, 1.5 Hz, 1 H), 8.27 (Ar–N**H**–CO, s, 1 H), 9.26 (Ar–N**H**–S, s, 1 H), 9.56 (Ar–N**H**–CO, s, 1 H).^13^C NMR (150 MHz, DMSO–*d*_*6*_; δ, ppm): 31.2 **(**S–**C**H_3_), 107.9 **(**Ar–**C**), 113.3 **(**Ar–**C**), 119.9 **(**Ar–**C**), 121.8 **(**Ar–**C**), 121.9 **(**Ar–**C**), 122.4 **(**Ar–**C**), 123.9 **(**Ar–**C**), 124.5 **(**Ar–**C**), 128.1 **(**Ar–**C**), 129.1 **(**Ar–**C**), 129.7 **(**Ar–**C**), 130.5 **(**Ar–**C**), 136.2 **(**Ar–**C**), 139.2 **(**Ar–**C**), 152.1 **(**Ar–**C**), 152.3 **(**Ar–**C**), 156.3 **(**NH–**C** **= O**).HRMS (ESI) m/z: calcd for C_20_H_18_N_3_SO_4_Cl [M + H]^+^ 432.07; found 432.08.

*N*-{4-[3-(4-Chloro-phenyl)-ureido]-2-phenoxy-phenyl}-methanesulfonamide (3h): White powder, Yield 86%, mp 237 °C, FT-IR υ_max_ (cm^−1^): 3313 (N–H), 3059 (Aromatic ring, =C–H), 2921 (Alkyl, C–H), 1635 (C=O), 1595 and 1546 (Aryl, C=C), 1489, 1334 (C–S), 1201(C–O), 1157(C–N), 825. ^1^H NMR (600 MHz, DMSO-*d*_*6*_; δ, ppm): 2.94 (S–C**H**_3_, s, 3h), 7.08–7.09 (3xAr–C**H**, m, 3h), 7.15 (Ar–C**H**, d, J = 7.4 Hz, 1H), 7.18 (Ar–C**H**, pt, J = 7.4 Hz 1H), 7.28 (3xAr–C**H**, d, J = 8.9 Hz, 3h), 7.40–7.44 (4xAr–C**H**, m, 4 H), 8.72 (Ar–N**H**–CO, s, 1H), 8.84 (Ar–N**H**–S, s, 1H), 9.18 (Ar–N**H**–CO, s, 1H). ^13^C NMR (150 MHz, DMSO-*d*_*6*_; δ, ppm): 39.7 **(**S**–C**H_3_), 107.3 **(**Ar–**C**), 112.5 **(**Ar–**C**), 118.6 **(**Ar–**C**), 119.2 **(**Ar–**C**), 120.8 **(**Ar–**C**), 123.2 **(**Ar–**C**), 124.9 **(**Ar–**C**), 126.8 **(**Ar–**C**), 127.9 **(**Ar–**C**), 128.1 **(**Ar–**C**), 129.4 **(**Ar–**C**), 137.8 **(**Ar–**C**), 137.9 **(**Ar–**C**), 138.2 **(**Ar–**C**), 150.8 **(**Ar–**C**), 151.6 **(**Ar–**C**), 151.8 **(**Ar–**C**), 155.4 **(**NH–**C** **= O**). HRMS (ESI) m/z: calcd for C_20_H_18_N_3_SO_4_Cl [M + H]^+^ 432.07; found 432.08.

*N*-[2-Phenoxy-4-(3-phenyl-ureido)-phenyl]-methanesulfonamide (3i): White powder, Yield 82%, mp 218 °C, FT-IR υ_max_ (cm^−1^): 3271 (N–H), 3059 (Aromatic ring, =C–H), 2929 (Alkyl, C–H), 1651 (C=O), 1589 and 1547 (Aryl, C=C), 1487, 1316 (C–S), 1219 (C–O), 1148 (C–N), 692.^1^ H NMR (600 MHz, DMSO-*d*_*6*_; δ, ppm): 2.94 (S–C**H**_3_, s, 3h), 6.94–6.98 (Ar–C**H**, m, 1 H), 7.07–7.09 (3xAr–C**H**, m, 3h), 7.16 (Ar–C**H**, d, J = 2.3hz, 1 H), 7.18 (Ar–C**H**, pt, J = 7.4 Hz, 1 H), 7.23–7.29 (3xAr–C**H**, m, 3h), 7.37 (2xAr–C**H**, d, J = 7.7 Hz, 2 H), 7.42–7.46 (2xAr–C**H**, m, 2 H), 8.57 (Ar–N**H**–CO, s, 1 H), 8.80 (Ar–N**H**–S, s, 1 H), 9.19 (Ar–N**H**–CO, s, 1 H). ^13^C NMR (150 MHz, DMSO-*d*_*6*_; δ, ppm): 40.2 **(**S**–C**H_3_), 107.6 **(**Ar–**C**), 112.9 **(**Ar–**C**), 118.0 **(**Ar–**C**), 118.2 **(**Ar–**C**), 119.1 **(**Ar–**C**), 121.0 **(**Ar–**C**), 121.7 **(**Ar–**C**), 121.8 **(**Ar–**C**), 123.7 **(**Ar–**C**), 128.5 **(**Ar–**C**), 128.6 **(**Ar–**C**), 128.7 **(**Ar–**C**), 129.9 **(**Ar–**C**), 138.9 **(**Ar–**C**), 139.3 **(**Ar–**C**), 151.4 **(**Ar–**C**), 152.1 **(**Ar–**C**), 155.9 **(**NH–**C** **= O**).HRMS (ESI) m/z: calcd for C_20_H_19_N_3_SO_4_ [M + H]^+^ 398.11; found 398.12.

*N*-[4-(3-Benzyl-ureido)-2-phenoxy-phenyl]-methanesulfonamide (3j): *White* powder, Yield 91%, mp 172 °C, FT-IR υ_max_ (cm^−1^): 3269 (N–H), 3054 (Aromatic ring, =C–H), 2930 (Alkyl, C–H), 1648 (C=O), 1551 and 1504 (Aryl, C=C), 1487, 1321 (C–S), 1216 (C–O), 1146 (C–N), 690.^1^H NMR (600 MHz, DMSO-*d*_*6*_; δ, ppm): 2.91 (S–C**H**_3_, s, 3h), 4.23 (NH–C**H**_2_–Ar, d, J = 5.9 Hz, 2 H), 6.55 (CH_2_–N**H**–CO, t, J = 6.0 Hz, 1 H), 7.03–7.06 (3xAr–C**H**, m, 3h), 7.13 (Ar–C**H**, d, J = 2.3hz, 1 H), 7.16 (Ar–C**H**, pt, J = 7.4 Hz, 1 H), 7.21–7.25 (4xAr–C**H**, m, 4 H), 7.31 (2xAr–C**H**, pt, J = 7.5 Hz, 2 H), 7.41 (2xAr–C**H**, pt, J = 8.0 Hz, 2 H), 8.72 (Ar–N**H**–S, s, 1 H), 9.12(Ar–N**H**–CO, s, 1 H). ^13^C NMR (150 MHz, DMSO-*d*_*6*_; δ, ppm): 40.1 **(**S–**C**H_3_), 42.5 **(**NH–**C**H_2_–Ar), 107.1 **(**Ar–**C**), 112.4 **(**Ar–**C**), 119.0 **(**Ar–**C**), 120.3 **(**Ar–**C**), 123.6 **(**Ar–**C**), 126.6 **(**Ar–**C**), 126.9 **(**Ar–**C**), 128.2 **(**Ar–**C**), 128.5 **(**Ar–**C**), 129.9 **(**Ar–**C**), 139.8 **(**Ar–**C**), 140.1 **(**Ar–**C**), 151.4 **(**Ar–**C**), 154.8 **(**Ar–**C**), 155.9 **(**NH–**C** **= O)**.HRMS (ESI) m/z: calcd for C_21_H_21_N_3_SO_4_ [M + H]^+^ 412.13; found 412.13.

*N*-{4-[3-(4-Methoxy-phenyl)-ureido]-2-phenoxy-phenyl}-methanesulfonamide (3k): White powder, Yield 89%, mp 228 °C, FT-IR υ_max_ (cm^−1^): 3304 (N–H), 3076 (Aromatic ring, =C–H), 2923 (Alkyl, C–H), 1708 (C=O), 1553 and 1504 (Aryl, C=C), 1440, 1334 (C–S), 1243 (C–O), 1154 (C–N), 825. ^1^H NMR (600 MHz, DMSO-*d*_*6*_; δ, ppm): 2.93 (S–C**H**_3_, s, 3h), 3.71 (OC**H**_3_, s, 3h), 7.06–7.08 (3xAr–C**H**, m, 3h), 7.15 (Ar–C**H**, d, J = 2.3hz, 1H), 7.18 (Ar–C**H**, pt, J = 7.4 Hz, 1H), 7.27 (3xAr–C**H**, dd, J = 8.9, 3.2 Hz, 3h), 7.33 (2xAr–C**H**, d, J = 9.0 Hz, 2 H), 7.42 (2xAr–C**H**, pt, J = 8.0 Hz, 2 H), 8.36 (Ar–N**H**–CO, s, 1H), 8.71 (Ar–N**H**–S, s, 1H), 9.16 (Ar–N**H**–CO, s, 1H). ^13^C NMR (150 MHz, DMSO-*d*_*6*_; δ, ppm): 40.1 **(**S–**C**H_3_), 54.9 **(**O**C**H_3_), 107.5 **(**Ar–**C**), 112.8 **(**Ar–**C**), 113.8 **(**Ar–**C**), 118.9 **(**Ar–**C**), 119.7 **(**Ar–**C**), 120.0 **(**Ar–**C**), 120.8 **(**Ar–**C**), 123.6 **(**Ar–**C**), 128.4 **(**Ar–**C**), 129.8 **(**Ar–**C**), 132.2 **(**Ar–**C**), 132.7 **(**Ar–**C**), 139.1 **(**Ar–**C**), 151.3 **(**Ar–**C**), 152.3 **(**Ar–**C**), 152.8 **(**Ar–**C**), 154.1 **(**Ar–**C**), 154.4 **(**Ar–**C**), 155.9 **(**NH–**C** **= O**). HRMS (ESI) m/z: calcd for C_21_H_21_N_3_SO_5_ [M + H]^+^ 428.12; found 428.13.

*N*-[4-(3-Phenethyl-ureido)-2-phenoxy-phenyl]-methanesulfonamide (3l): White powder, Yield 85%, mp 199 °C, FT-IR υ_max_ (cm^−1^): 3271 (N–H), 3056 (Aromatic ring, =C–H), 2928 (Alkyl, C–H), 1646 (C=O), 1562 and 1504 (Aryl, C=C), 1489, 1327 (C–S), 1214 (C–O), 1152 (C–N), 625.^1^H NMR (600 MHz, DMSO-*d*_*6*_; δ, ppm): 2.69 (C**H**_2_CH_2_NH, t, J = 7.2 Hz, 2 H), 2.91 (C**H**_3_, s, 3h), 3.27 ( CH_2_C**H**_2_NH, dd, J = 13.2, 6.8 Hz, 2 H), 6.04 ( CH_2_–N**H**–CO, t, J = 5.6 Hz, 1 H), 7.00 (Ar–C**H**, dd, J = 8.7, 2.2 Hz, 1 H), 7.05 (2xAr–C**H**, d, J = 7.9 Hz, 2 H), 7.11 (Ar–C**H**, d, J = 2.2 Hz, 1 H), 7.15–7.22 (5xAr–C**H**, m, 5 H), 7.28 (2xAr–C**H**, pt, J = 7.5 Hz, 2 H), 7.41 (2xAr–C**H**, pt, J = 7.9 Hz, 2 H), 8.62 (Ar–N**H**–S, s, 1 H), 9.08 (Ar–N**H**–CO, s, 1 H).^13^C NMR (150 MHz, DMSO-*d*_*6*_; δ, ppm): 30.7 **(C**H_2_), 35.7 **(C**H_2_), 40.2 **(C**H_3_), 107.2 **(**Ar–**C**), 112.5 **(**Ar–**C**), 119.1 **(**Ar–**C**), 123.7 **(**Ar–**C**), 126.1 **(**Ar–**C**), 128.3 **(**Ar–**C**), 128.6 **(**Ar–**C**), 129.9 **(**Ar–**C**), 139.4 **(**Ar–**C**), 139.9 **(**Ar–**C**), 151.5 **(**Ar–**C**), 154.8 **(**Ar–**C**), 156.1 **(**NH–**C= O**). HRMS (ESI) m/z: calcd for C_22_H_23_N_3_SO_4_ [M + H]^+^ 426.14; found 426.15.

### Antioxidant assay

The DPPH (2,2-diphenyl-1-picrylhydrazyl) radical scavenging capacity of the compounds was assessed following a previously reported method in the literature^54–56^. A suitable dilution series (250–1000 µg/mL) was prepared for each compound in methanol. Subsequently, 0.5 mL of each sample was combined with 0.5 mL of a 0.15 mM methanolic DPPH solution. The mixture was then incubated in the dark at room temperature for 30 min, after which the absorbance was measured at 517 nm using methanol as the blank. The equation below illustrates the calculation of the DPPH radical scavenging capacity.

$${\text{DPPH radical scavenging activity}}\left( \% \right)=~\frac{{{A_{{\mathrm{Control}} }}-{A_{{\mathrm{Sample}}}}}}{{{A_{{\mathrm{Control}}}}}} \times 100$$

A_Control_ denotes the absorbance of the control solution (without the test compound), while A_Sample_ denotes the absorbance measured in the presence of the test compound.

### Cytotoxicity assay

MDA-MB-231 (human triple negative breast cancer cells), HeLa (human cervical cancer cells) PC-3 (human prostate cancer cells), MKN-45 (human gastric cancer cells), U87 (human gliablostoma cancer cells) and HUVEC (human umbilical vein endothelial cells were used. Cells were grown and proliferated in medium containing 10% foetal bovine serum (FBS), 1% penicillin-streptomycin, 2 mM L-glutamine and 2000 mg/mL sodium bicarbonate in an incubator at 5% CO_2_ and 75% humidity at 37 °C. MDA-MB-231, HeLa, MKN-45 and U87 cells were grown in DMEM medium and PC- 3 cells were grown in RPMI-1640. HUVEC cells were grown in F12 medium (Kaighn’s Modification of Ham’s F-12 medium) with the addition of 0.04 mg/mL endothelial cell growth supplement.

### MTT assay

The effect of the synthesized compounds was determined using the MTT method on MDA-MB-231, HELA, PC-3, MKN-45, U87 and HUVEC cells^57^. IC_50_ values were calculated. For this purpose, 5 × 10^3^ MDA-MB-231, HeLa, PC-3, MKN-45, U87 and HUVEC cells were seeded in 100 µL of medium in 96-well plates. After ensuring cell adhesion, the synthesised compounds were added to the cells at concentrations of 0.1, 1, 10, 100 and 1000 µM and incubated for 24 h. After incubation, 10µL MTT solution (ODC Research and Development ODC0009D-250 Cytotoxicity assay kit) was added and incubated for 4 h. To dissolve the formazan dye formed, the medium was aspirated and 100 µL of solubilising solution was added to each well. The absorbance of the resulting colour was measured at 570 nm in a microplate reader. The MTT assay was performed in triplicate. The viability rate, the calculation of IC_50_ and the statistical analyses were calculated using the program of the GraphPad Prism package. The selectivity index (SI) was calculated based on the ratio of cytotoxic effects observed in normal control cells to those observed in cancer cells after 24 h of exposure. Based on the values obtained, compounds that could exhibit anti-cancer effects were selected, and studies were continued in the designated cell line. An SI value greater than 1 indicates higher selectivity toward cancer cells compared to normal cells. Images of cells treated with selected compounds of IC50 values (µM) were captured using an inverted microscope after 24 h of incubation.

### AO/EB staining

Acridine orange/ethidium bromide (AO/EB) staining was performed for evaluation of cell death^58^. 5 × 10^4^ MDA-MB-231 cells were seeded in 800 µL of medium in 12-well plates. After cell attachment, synthetic compounds **3i**,** 3j** and **3l** were added at a concentration of IC50 values (µM), paclitaxel 15 nM concentration was added to cells and incubated for 24 h. Following incubation, staining was performed using a commercial kit (ODC0010D, AO/EB staining kit ODC Research and Development Inc.). After being incubated with the compounds for 24 h, 10 µL AO/AB dye was applied to the cells and incubated for 15 min. At the end of the incubation, the plates were washed and the images of the cells were evaluated under an inverted fluorescence microscope (Zeiss Axiovert).

### Annexin-V-PI assay

MDA-MB-231 cells were seeded in 6-well plates at a density of 1 × 10^6^ cells per well and incubated for 16 h with IC50 values of the compounds **3i**, **3j** and **3l**^59^. Following the incubation period, the cell medium was removed and the cells were washed twice with PBS. Subsequently, the cells were detached using trypsin-EDTA and centrifuged at 500 x g for 10 min. The cells were prepared in accordance with the manufacturer’s instructions using the Annexin-V-FITC kit (BD Pharmigen, 556570) and suspended in an annexin binding buffer. A total of 100 µL of the cell suspension was obtained, and 5 µL of both annexin V and PI were subsequently added. The mixture was then incubated for 30 min in the dark at room temperature. Subsequently, 20,000 cells were analyzed using a flow cytometer (Novocyte, Agilent), and the gate was selected for the examination of apoptotic cells and live cells.

### Measurement of Bax and Bcl-2 protein levels

The cells were seeded in 6-well plates and the compounds **3i**, **3j** and **3l** were incubated at a concentration of IC50 values for a period of 24 hours^60^. Following the incubation period, the MDA-MB-231 cells were harvested and lysed using lysis buffer. ELISA kits (Bax [ODC00012eh], Human Bax ELISA kit; and Bcl-2 [ODC00013eh], Human Bcl-2 ELISA kit) were employed to analyze Bax, and Bcl-2, respectively, according to the instructions outlined in the manufacturer’s protocol. Subsequently, the samples were loaded onto the ELISA plates and washed with the wash buffer following the binding of the antibodies. Following the addition of the secondary antibody and subsequent washing step, the substrate was introduced and read at 450 nm in an ELISA reader (Epoch, Biotek).

### Scratch assay

Cells were seeded in a 24-well plate and a scratch was made in the middle of the well with a yellow pipette tip^61^. Then, **3i**,** 3j** and **3l** compounds were placed on the cells and 0th hour images were taken at the time of the first placement. After 24 h of incubation, images were retaken again under a microscope (Zeiss Axiovert, 10X magnification) and the gap closure intervals were analyzed.

### METAP2 enzyme activity assay

The effects of the synthesized compounds on METAP2 enzyme activity were determined using an in vitro enzymatic assay as previously described^62,63^. Recombinant METAP2 enzyme (APREST74783, Sigma-Aldrich, PrEST Antigen METAP2) was obtained commercially and used at a concentration of 1 µg. The enzyme was maintained under cold chain conditions to preserve activity until use. Reaction mixtures were prepared in 50 mM MOPS buffer (pH 7.0) containing 50 µM CoCl₂. Each assay was performed in a final volume of 100 µL, consisting of 1 µg METAP2 enzyme. Synthesized compounds were added at a final concentration of 100 µM (2 µL volume) and incubated with the enzyme for 15 min at room temperature. The reaction was initiated by the addition of 400 µM L-Methionine 7-amido-4-methylcoumarin trifluoroacetate (Met-AMC), a fluorogenic peptide substrate, followed by incubation for 90 min at 37 °C. Enzyme activity was monitored using a fluorescence spectrometer (PerkinElmer, FL 6500) at excitation and emission wavelengths of 360 nm and 460 nm, respectively. The relative fluorescence intensity was used to assess METAP2 activity in the presence of the test compounds.

### Molecular modeling studies

MetAP2 is a critical protein processing enzyme and a validated target for anticancer and antiangiogenic therapies. Potent inhibitors can hinder enzyme activity by stabilizing inactive conformations or preventing the access of substrates. To identify promising inhibitors, computational approaches provide valuable insights into the conformational dynamics and stability that ligand binding imparts. For this purpose, molecular docking studies were conducted to predict the preferred binding orientations and interactions of the 12 nimesulide ureas (**3a-l**) with the active site of MetAP2. These are subsequently refined through molecular dynamics (MD) simulations to inform about the stability and conformational flexibility of the ligand-protein complexes over time^64^. Finally, MM/GBSA (Molecular Mechanics/Generalized Born Surface Area) calculations were carried out to estimate the binding free energies, providing quantitative analysis of the ligands’ binding affinities toward MetAP2.

### Enzyme data set and setup

The 3D crystal structure of the human Methionine aminopeptidase 2 (PDB ID: 5CLS) in complex with a spiroepoxytriazole inhibitor and resolved at 1.75 Å, was downloaded from the RCSB Protein Data Bank (https://www.rcsb.org/). Using Desmond’s “protein preparation” procedure, all water molecules, native ligands, and salt ions were removed, and hydrogen atoms were added at physiological pH of 7.4. After the proper protonation and refinement procedure, the MetAP2 enzyme was then briefly energy minimized.

### Ligand setup and molecular docking

All 12 novel nimesulide ureas (**3a-l**) were initially drawn using ChemBioDraw Ultra and saved as SDF files. Next, the “Prepare Ligands” protocol in BIOVIA DS 4.5 was used to protonate all ionizable groups at pH 7.4, followed by 3D geometry optimization with the “clean geometry” protocol^65^. The ligands were then saved again in SDF format.

The nimesulide ureas (**3a-l**) were docked into the active site of the MetAP2 enzyme using AutoDock Vina^66^. Input files for docking were generated with the Lamarckian genetic algorithm. The grid box was set to dimensions X = 26.53 Å, Y = 21.30 Å, Z = 17.55 Å, with a grid size of 60 × 60 × 60, carefully covering the entire active site. The spacing was fixed at 0.375 Å, and each ligand was docked with ten runs up to 2,500,000 evaluations^67^.

### Molecular dynamics simulation

Molecular dynamics (MD) simulations were conducted to investigate the stabilizing effects of 12 ligands on the MetAP2 enzyme, using the Desmond software package with the OPLS2005 force field^68^ for 200 nanoseconds. The process involves three main steps: protein preparation, system building, and MD simulation, each following standardized protocols. The systems were solvated in an orthorhombic water box using the SPC water model, maintaining a 10 Å buffer between the protein surface and the box boundaries. To mimic physiological conditions, the systems were neutralized by adding appropriate amounts of Na^+^ and Cl^–^ ions to achieve a 0.15 M salt concentration. Before the production run, energy minimization and relaxation protocols were applied in Desmond using default settings to eliminate steric clashes. The temperature was maintained at 303.15 K in an NPT ensemble, with pressure held constant at 1.013 bar throughout the simulation.

### MM/GBSA energy calculations

The molecular mechanics generalized Born surface area (MM/GBSA) calculations are of are highly important in quantitatively providing a more accurate estimation of binding free energies by considering both molecular mechanics interactions and solvation effects. Unlike docking scores or initial simulation energies, MM/GBSA accounts for the energetic contributions of solvation and desolvation processes crucial for ligand-receptor binding affinities^69^. The binding affinities of the 12 complexes (MetAP2 with **3a-l**) were evaluated by calculating the MM/GBSA energies from the last 20 ns of the molecular dynamics trajectories using the thermal_mmgbsa.py script, and the average MM/GBSA energy for each ligand was subsequently determined. This method provided detailed insights into various energy contributions, including van der Waals, electrostatic, and hydrophobic energies, which collectively contributed to the total binding energy, calculated as the sum of these individual components.

## Results and discussion

### Chemistry

**Scheme 1:**
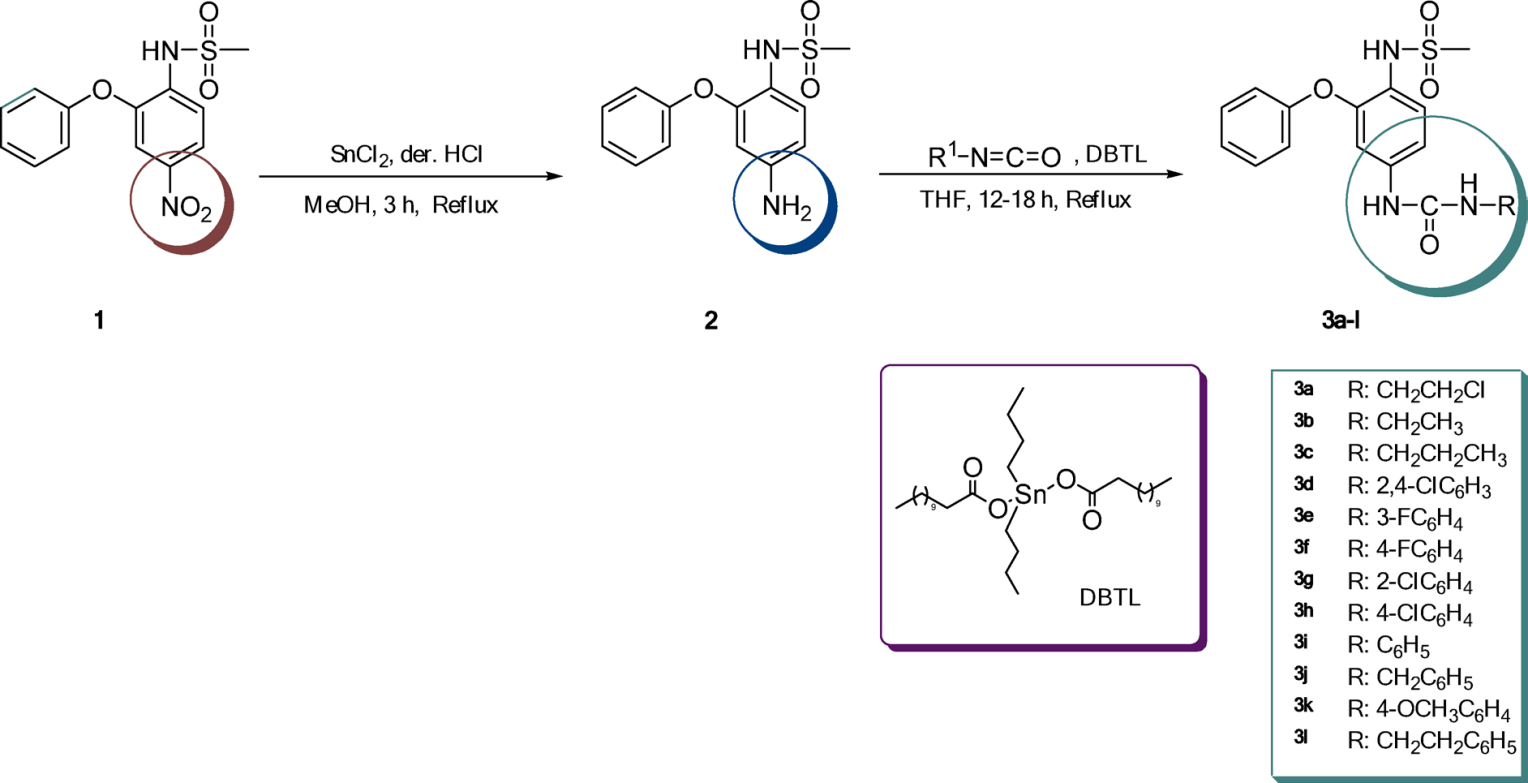
Shows the synthetic route used to synthesize novel nimesulide ureas.

A series of novel nimesulid ureas (**3a-l**) was efficiently synthesized via the reaction of reduced nimesulide with various aliphatic and aromatic isocyanates in the presence of DBTL (dibutyltin dilaurate) as a catalyst, following the synthetic route depicted in Scheme 1. The desired products were obtained in good to excellent yields, ranging from 68% to 91%.

Nimesulide was subjected to hydrogenation in the presence of tin(II) chloride (SnCl_2_), affording the reduced form of nimesulide (**2**). As an aromatic primary amine, the reduced nimesulide exhibits diminished nucleophilicity due to the delocalization of the nitrogen lone pair into the aromatic π-system. This delocalization considerably limits its nucleophilic reactivity; consequently, the reaction proceeds with significantly lower yields in the absence of a catalyst. To determine the optimum reaction conditions, a series of experiments was conducted using compound **3i** as a model substrate in the presence of different catalysts, including triethylamine (Et_3_N), pyridine, and dibutyltin dilaurate (DBTL). Among all the conditions evaluated, the use of 1.5 equivalents of both isocyanate and DBTL proved to be the most effective, delivering the target product with a yield of 82% (molar ratio of compound **2**: 1.0 equivalent in all experiments; see Table 1 footnote).

**Table 1:** Reaction condition screening for nimesulide-derived urea synthesis.


Entry	Isocyanate (eq)	Catalyst (eq)	Catalyst	Solvent	t (^o^C)	Time (h)	Yield (%)
1	1.1	–	–	THF	25	18	20
2	1.1	–	–	Toluene	110	18	27
3	1.1	1.0	Pyridine	THF	66	18	32
4	1.1	1.0	Pyridine	Toluene	110	18	35
5	1.1	1.0	NEt_3_	THF	66	18	34
6	1.1	1.0	NEt_3_	Toluene	110	18	37
7	1.1	1.0	DBTL	THF	66	18	58
8	1.1	1.5	DBTL	THF	66	18	74
9	**1.5**	**1.5**	**DBTL**	**THF**	**66**	**18**	**82**

*Molar ratio of compound **2**: 1.0.

Significance value bold.

The molecular structures of the synthesized nimesulide-derived ureas **3a–l** were elucidated using spectroscopic (IR, ¹H NMR, ¹³C NMR and HR-MS) analysis methods (Figs. S1–S52, Supporting Information).

Upon examination of the^1^ H NMR spectrum of reduced nimesulide (**2**), the primary amine (Ar–N**H**₂) protons appeared as a singlet at δ 5.26 ppm. In the FT-IR spectrum of compound **2**, absorption bands were observed at around 3397 and 3330 cm^−1^, corresponding to the **N**–**H** stretching vibrations of the secondary amine group. The aliphatic and aromatic carbon signals of reduced nimesulide (**2**) were seen at their expected chemical shift values. Furthermore, the MS spectra of compound **2** indicated that the molecular ion peaks corresponded precisely to its molecular weight.

In the^1^ H NMR spectra of the nimesulide-derived urea compounds (**3a–l**), the aromatic Ar–NH_2_ proton signal observed at 5.26 ppm in reduced nimesulide (**2**) disappeared, while two distinct NH proton signals corresponding to the urea functionality, assigned to Ar–CO–NH and Ar–NH–SO_2_ groups, emerged in the range of 8.53–9.56 ppm. These observations confirm the successful formation of the target urea derivatives. Signals corresponding to the S–C**H**_3_ protons of the nimesulide moiety were observed as singlets at δ 2.91–3.01 ppm in the^1^ H NMR spectra of compounds **3a–l**. The aromatic protons exhibited characteristic doublets, triplets, and multiplets within the chemical shift range of δ 6.75–8.14 ppm. In the^1^ H NMR spectra, singlet peaks corresponding to the Ar–N**H**–S and Ar–N**H**–CO protons were observed in the chemical shift ranges of δ 8.53–9.26 ppm and δ 9.08–9.56 ppm, respectively. The CO–N**H**–R protons were observed as singlets at substituent-dependent chemical shifts in the synthesized nimesulide-derived urea compounds. For aliphatic substituents (**3b**, **3c**, **3j**, **3l**), these protons resonated in the range of δ 6.01–6.55 ppm, whereas for aromatic substituents (**3a**, **3d**, **3e**, **3f**, **3g**, **3h**, **3i**, **3k**), they appeared at δ 8.27–8.80 ppm.

Analysis of the^13^ C NMR spectra of compounds **3a-l** revealed NH–**C**=O signals at δ 155.38–163.12 ppm. The signals corresponding to the S–**C**H_3_ carbon atoms were observed in the^13^ C NMR spectra within the chemical shift range of δ 31.17–40.33 ppm. The aromatic carbons exhibited resonances in the chemical shift range of δ 107.14–161.53 ppm.

In the FT-IR spectra, weak **N–H** bands were observed at 3341–3256 cm^− 1^ in all target compounds. Carbonyl (**C=O**) group bands of the urea chain appeared as strong absorptions in the 1721–1635 cm^−1^ range. The peaks of aromatic ring vibrations were observed between 3089 − 3022 and 1611 –1504 cm^−1^.

HR-MS spectra of the compounds showed that molecular ion peaks exactly matched the molecular weights of all the synthesized compounds **3a-l**.

### Antioxidant activity

Free radicals, characterized by their high reactivity and unpaired electrons, are produced by various metabolic processes in living organisms, ultimately leading to cellular damage. Antioxidants are molecular compounds that mitigate or inhibit the detrimental effects of oxidative stress caused by free radicals within biological systems. Numerous studies have highlighted the potential antioxidant properties of nimesulide^70^. In this study, the potential antioxidant activity of the novel synthesized nimesulide ureas (**3a-l**) was assessed in comparison to standard antioxidants, including ascorbic acid, quercetin, butylated hydroxytoluene (BHT), and butylated hydroxyanisole (BHA), using the DPPH radical scavenging assay. DPPH (1,1-diphenyl-2-picryl-hydrazyl) is a stable free radical that exhibits a characteristic purple coloration at 517 nm in methanol and can accept either an electron or a hydrogen radical. The DPPH radical changes color from purple to yellow after reacting with various reducing agents or antioxidants. The extent of DPPH radical reduction is quantitatively determined by measuring the decrease in absorbance at 517 nm.

Solutions of novel nimesulide ureas (**3a-l**), along with standard antioxidants, were prepared in methanol at different concentrations (250, 500, and 1000 µg/mL), and each experiment was conducted in triplicate (Table 2).

Among the evaluated compounds, **3f** demonstrated DPPH radical scavenging activity approaching that of the standard antioxidants at a concentration of 1000 µg/mL.

**Table 2:** DPPH scavenging activities of novel nimesulide ureas (**3a-l**).

Compounds and standarts	DPPH (µg/mL)		
	250	500	1000
**3a**	17.91 ± 0.69	24.74 ± 3.25	33.81 ± 4.33
**3b**	38.18 ± 2.61	56.38 ± 2.66	65.20 ± 0.73
**3c**	45.98 ± 1.83	65.85 ± 2.62	77.77 ± 3.26
**3d**	31.36 ± 3.82	56.89 ± 5.88	61.53 ± 4.37
**3e**	24.92 ± 0.15	25.25 ± 3.20	32.59 ± 3.28
**3f**	**78.71 ± 4.35**	**87.11 ± 0.40**	**92.93 ± 4.23**
**3g**	15.32 ± 3.94	23.76 ± 3.09	37.57 ± 1.67
**3h**	30.21 ± 2.39	30.64 ± 2.14	46.12 ± 3.70
**3i**	44.68 ± 3.70	57.50 ± 6.25	74.95 ± 1.84
**3j**	33.22 ± 1.29	50.01 ± 0.85	67.60 ± 0.89
**3k**	43.00 ± 5.47	45.90 ± 3.77	81.90 ± 0.52
**3l**	41.00 ± 5.01	60.10 ± 5.58	60.65 ± 4.08
BHT	95.10 ± 1.52	96.22 ± 2.63	97.13 ± 3.00
BHA	94.39 ± 0.52	96.87 ± 0.69	97.51 ± 0.50
Quarcetin	91.41 ± 0.36	91.66 ± 0.27	91.80 ± 0.67
Ascorbic acid	97.51 ± 0.43	98.01 ± 1.74	98.76 ± 0.45

Significance value bold.

### Cytotoxicity assay

To further support the selection of the cancer models used in this study, expression levels of METAP2 across multiple tumor types were examined using publicly available datasets from The Cancer Genome Atlas (TCGA). Analysis of TCGA datasets corresponding to breast (BRCA), cervical (CESC), prostate (PRAD), gastric (STAD), and glioblastoma (GBM) cancers demonstrated detectable expression of METAP2 across these tumor types. These findings support the relevance of the selected cell lines (MDA-MB-231, HeLa, PC-3, MKN-45, and U87) as representative experimental models for evaluating the biological activity of the studied compounds in METAP2-related cancer contexts.

The IC50 values of compounds synthesized in MDA-MB-231, HeLa, PC-3, MKN-45, U87 and HUVEC cells were calculated (Table 3). The compounds that were effective against cancer cells at 3 µM and not toxic to HUVEC cells were determined by SI (Table 4). While compound **3i** was effective at 2.62 ± 1.16 µM concentration in MDA-MB-231 cells, it was effective at 18.62 ± 1.30 µM in HUVEC cells. Compound **3j** was effective at 0.89 ± 0.03 µM in MDA-MB-231 cells and at 66.05 ± 1.68 µM in HUVEC cells. While compound **3l** was effective at 0.66 ± 0.08 µM concentration in MDA-MB 231 cells, it was effective at 121.13 ± 3.47 µM in HUVEC cells. Paclitaxel, used as a positive control, was effective at 17.24 ± 2.66 µM in MDA-MB-231 cells and at 72.91 ± 2.08 µM in HUVEC cells. Compounds **3i**, **3j**, and **3l** were chosen for apoptosis studies due to their pronounced cytotoxic activity against MDA-MB-231 cells, together with relatively lower toxicity toward HUVEC cells, indicating enhanced anticancer selectivity.

**Table 3:** IC_50_ values (µM) of novel nimesulide isoureas (**3a-l**).

Comp. No	HUVEC (µM)	HELA (µM)	MDA-MB-231 (µM)	PC-3 (µM)	MKN-45 (µM)	U87 (µM)
**3a**	76.79 ± 5.48	121.03 ± 3.10	260.01 ± 4.32	425.35 ± 5.14	98.22 ± 6.24	384.59 ± 9.11
**3b**	64.25 ± 5.32	120.15 ± 7.63	102.84 ± 6.09	718.05 ± 9.33	36.53 ± 4.71	95.59 ± 4.06
**3c**	75.93 ± 6.65	474.69 ± 8.03	100.66 ± 4.08	582.79 ± 6.66	73.16 ± 5.09	70.35 ± 0.88
**3d**	358.97 ± 8.12	414.30 ± 6.58	376.02 ± 3.02	43.88 ± 7.24	596.40 ± 5.18	21.70 ± 2.15
**3e**	348.77 ± 4.14	123.49 ± 8.84	419.21 ± 4.25	623.22 ± 13.06	139.10 ± 10.16	30.48 ± 1.68
**3f**	0.52 ± 0.26	114.04 ± 7.92	21.79 ± 6.55	28.49 ± 2.67	100.51 ± 4.15	0.29 ± 0.05
**3g**	0.192 ± 0.21	9.39 ± 3.44	330.31 ± 2.42	268.11 ± 2.93	76.59 ± 6.11	20.71 ± 0.16
**3h**	539.28 ± 9.87	616.06 ± 9.17	103.88 ± 4.73	292.02 ± 10.88	623.53 ± 8.17	255.90 ± 7.32
**3i**	**18.62 ± 1.30**	**106.12 ± 14.22**	**2.62 ± 1.16**	**716.23 ± 6.76**	**554.05 ± 9.28**	**11.67 ± 0.41**
**3j**	**66.05 ± 1.68**	**9.31 ± 2.75**	**0.89 ± 0.03**	**10.52 ± 0.08**	**27.62 ± 3.04**	**7.55 ± 0.20**
**3k**	422.21 ± 0.14	11.42 ± 0.53	465.68 ± 12.06	25.52 ± 5.12	97.72 ± 3.86	33.74 ± 0.83
**3l**	**121.13 ± 3.47**	**57.53 ± 3.96**	**0.66 ± 0.08**	**9.42 ± 1.30**	**14.85 ± 1.35**	**10.77 ± 0.04**
Paclitaxel	72.91 ± 2.08	11.32 ± 2.41	17.24 ± 2.66	14.06 ± 3.03	22.07 ± 3.56	19.25 ± 3.02

Significance value bold.

**Fig. 1:**
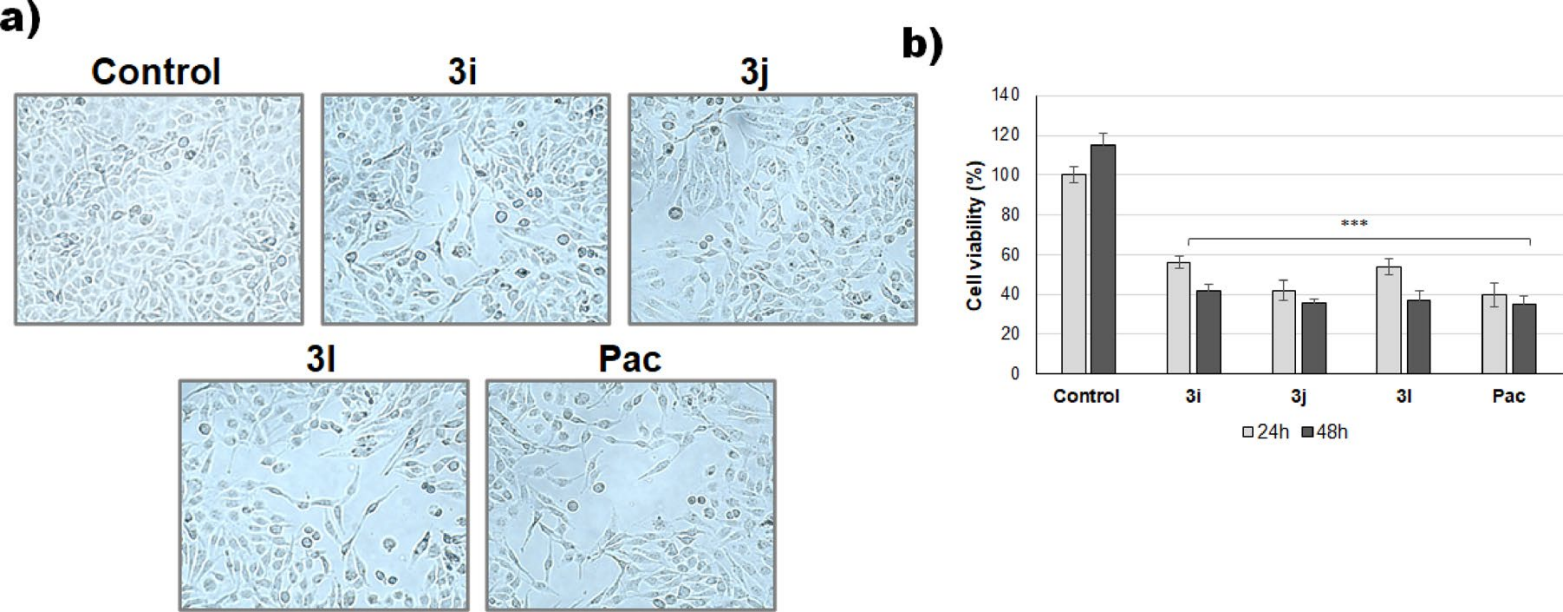
Effects of the target compounds on the morphology and viability of MDA-MB-231 cells. Cells were treated with the compounds at their IC₅₀ concentrations (µM) and evaluated after 24 and 48 h. (**a**) Representative microscopic images showing morphological changes in MDA-MB-231 cells after 24 h of treatment. Scale bar: 50 μm. (**b**) Quantitative analysis of cell viability ratios in MDA-MB-231 cells following 24 and 48 h of treatment. All experiments were performed in triplicate (*n* = 3), and data are presented as mean ± SD.

The morphology and cell viability of the compounds were evaluated in MDA-MB-231 cells at IC50 values (µM) after 24- and 48-hour incubations. It was determined that the compounds exhibited diminished interaction with one another and exhibited a decelerated growth rate compared to the control group, as evidenced by morphological alterations in the cells (Fig. 1a). While a decrease in cell viability was observed for compounds **3i**,** 3j** and **3l** at 24 h of incubation, a significantly greater decrease was observed for the same compounds at 48 h of incubation (Fig. 1b; *p* < 0.001).

**Table 4:** Selectivity index (SI) values of novel nimesulide urea derivatives (**3a–l**) after 24 h of treatment.

	HUVEC/HELA	HUVEC/ MDA-MB-231	HUVEC/PC-3	HUVEC/MKN-45	HUVEC/U87
3a	0.634	0.295	0.181	0.782	0.200
**3b**	0.535	0.625	0.089	1.759	0.672
**3c**	0.160	0.754	0.130	1.038	1.079
**3d**	0.866	0.955	8.179	0.602	16.541
**3e**	2.824	0.832	0.560	2.507	11.440
**3f**	0.005	0.024	0.018	0.005	1.786
**3g**	0.020	0.001	0.001	0.003	0.009
**3h**	0.875	5.191	1.847	0.865	2.107
**3i**	**0.175**	**7.107**	**0.026**	**0.034**	**1.595**
**3j**	**7.089**	**74.213**	**6.277**	**2.391**	**8.748**
**3k**	36.965	0.907	16.541	4.320	12.511
**3l**	**2.105**	**183.530**	**12.859**	**8.155**	**11.242**
Paclitaxel	6.441	4.229	5.186	3.304	3.788

*SI = IC₅₀ (normal cells)/IC₅₀ (cancer cells).

Significance value bold.

**Fig. 2:**
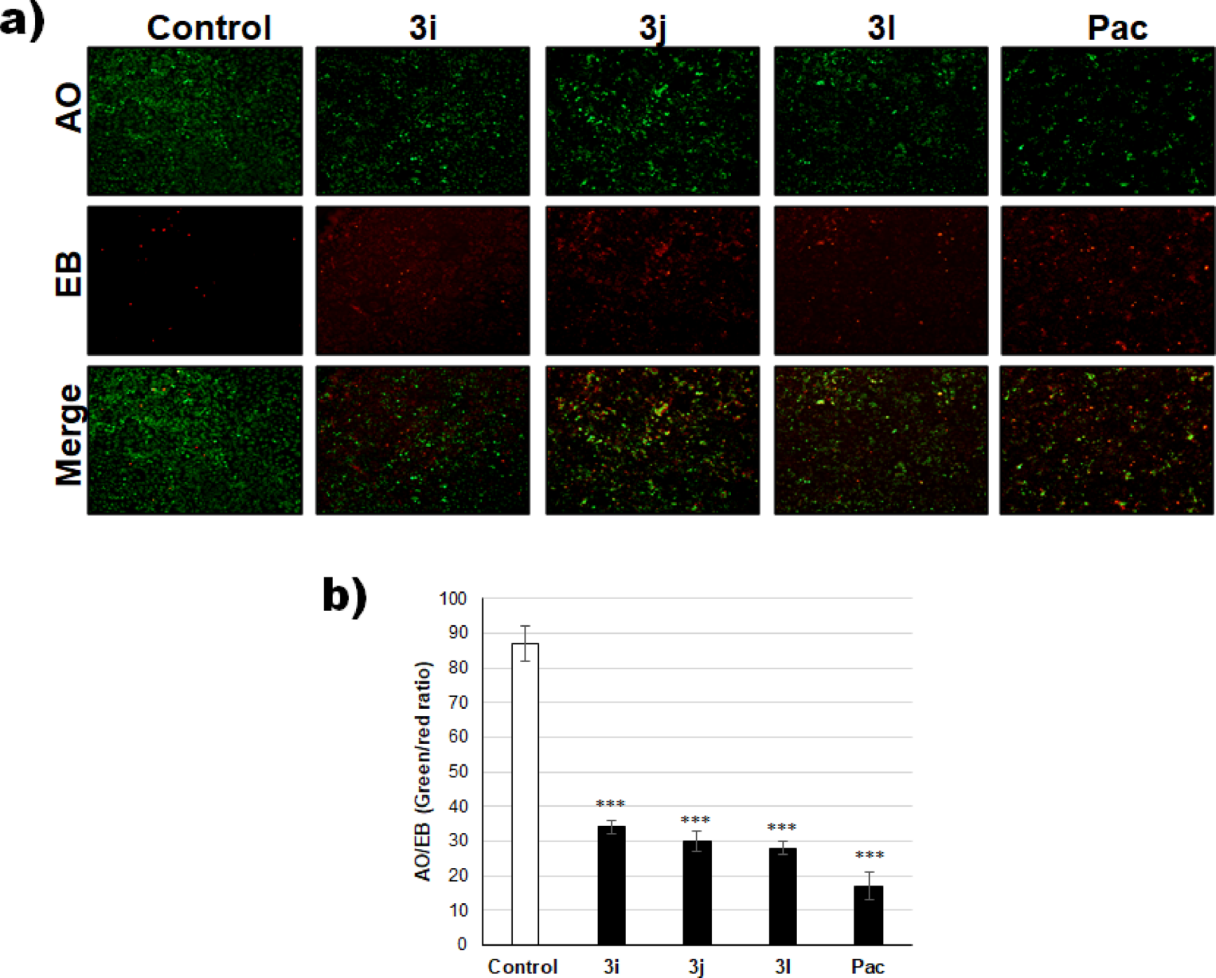
Assessment of apoptosis in MDA-MB-231 cells using acridine orange/ethidium bromide (AO/EB) staining after treatment with the target compounds. Cells were treated with the compounds at their IC₅₀ concentrations (µM) for 24 h and subsequently stained with AO/EB. (**a**) Representative fluorescence microscopy images of stained cells. The upper panel shows acridine orange (AO, green fluorescence), indicating viable or early apoptotic cells; the middle panel shows ethidium bromide (EB, red fluorescence), indicating membrane-compromised late apoptotic or necrotic cells; and the lower panel shows the merged images (orange), illustrating the overlap of AO and EB signals. Scale bar: 50 μm. (**b**) Quantitative analysis of AO/EB fluorescence ratios in MDA-MB-231 cells after 24 h treatment at IC₅₀ concentrations.

AO/EB staining is a method employed to identify cells undergoing apoptosis. AO stains are indicative of cellular vitality and produce a green fluorescent signal. EB has the capacity to bind to the DNA of cells that have undergone apoptosis. As the cell membrane begins to deteriorate and DNA breaks in dead cells, EB immediately enters the cell and stains it, increasing the red color intensity. The combination of AO and EB stains in a single cell reveals an increase in cell death when the colors within the cell begin to transition from green to orange. The AO/EB ratio is evaluated as green/red, and the resulting ratio is employed to assess cell death (Fig. 2a). In MDA-MB-231 cells, compounds **3i**,** 3j** and **3l** were observed to induce cell death at a rate exceeding that of control cells by more than twofold, accompanied by a notable decline in the AO/EB ratio. The AO/EB ratio was significantly altered by paclitaxel (Fig. 2b, *p* < 0.001).

**Fig. 3:**
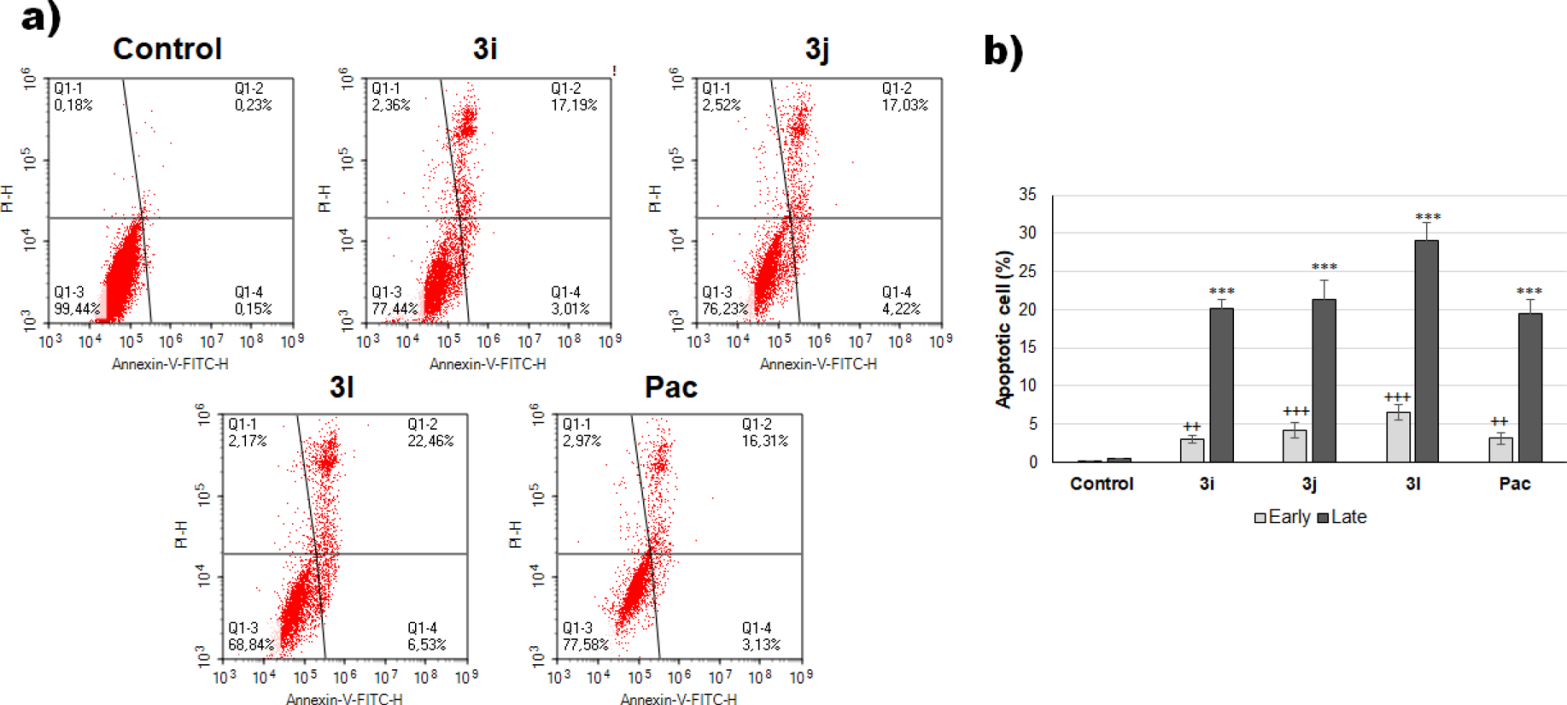
Evaluation of apoptosis in MDA-MB-231 cells by Annexin V-FITC/propidium iodide (PI) staining following treatment with the target compounds. Cells were treated with the compounds at their IC₅₀ concentrations (µM) for 24 h and analyzed by flow cytometry. (**a**) Representative flow cytometry dot plots showing the distribution of cell populations based on Annexin V-FITC and PI staining. Quadrant analysis indicates: Q1-3, viable cells (Annexin V⁻/PI⁻); Q1-4, early apoptotic cells (Annexin V⁺/PI⁻); Q1-2, late apoptotic cells (Annexin V⁺/PI⁺); and Q1-1, necrotic cells (Annexin V⁻/PI⁺). (**b**) Quantitative analysis of apoptotic cell populations in MDA-MB-231 cells after 24 h of treatment. The total apoptotic cell ratio was calculated as the sum of early apoptotic (Q1-4) and late apoptotic (Q1-2) cell percentages.

Annexin V-PI binding is also a reliable indicator of apoptosis. Phosphatidylserine, which is located on the inner surface of the cell membrane, is externalized when apoptotic signals are initiated within the cell, whereupon annexin-V binds to it. Propidium iodide (PI) is a fluorescent dye that binds to DNA. Its binding to DNA increases in cells undergoing late apoptosis and necrosis. The determination of apoptosis rates depends on observing the binding of these dyes to one another. The early and late apoptosis of MDA-MB-231 cells was quantified by flow cytometry following incubation with the **3i**,** 3j**, and **3l** compounds at 16 h. The resulting data were used to calculate the levels of early and late apoptosis (Fig. 3). The early apoptosis levels of compounds **3i**,** 3j** and **3l** were found to be greater than 3%. In contrast, the late apoptosis levels were observed to be more than 20%, in comparison to the control group in MDA-MB-231 cells (*p* < 0.001).

**Fig. 4:**
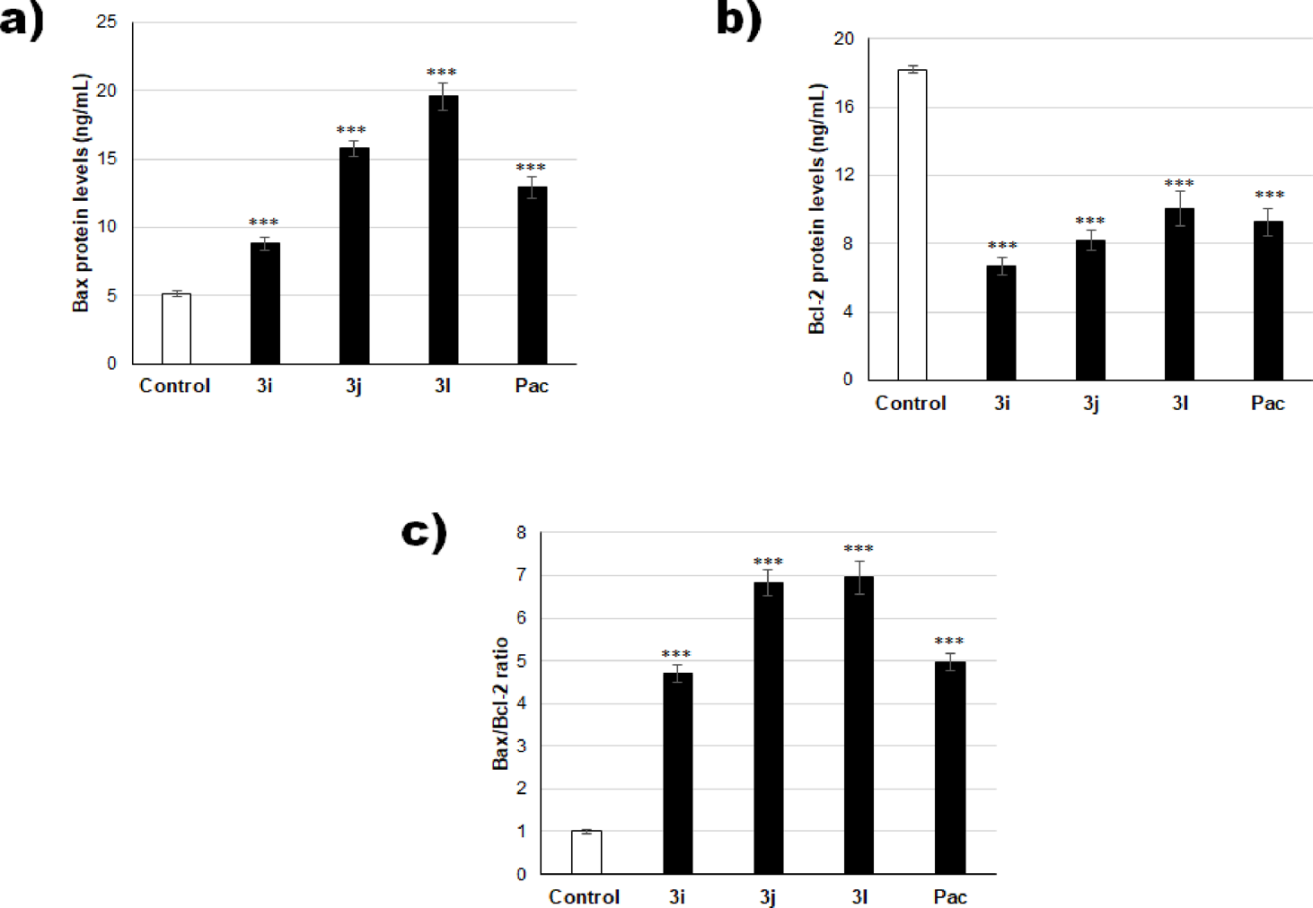
Effects of the target compounds on apoptosis-related protein levels in MDA-MB-231 cells. Cells were treated with the compounds at their IC₅₀ concentrations (µM) for 24 h, and the protein expression levels were determined using enzyme-linked immunosorbent assay (ELISA). (**a**) Bax protein expression levels, (**b**) Bcl-2 protein expression levels, and (**c**) the Bax/Bcl-2 ratio in MDA-MB-231 cells after treatment. All experiments were performed in triplicate (*n* = 3), and the data are presented as mean ± SD.

Two fundamental proteins, Bax and Bcl-2, are involved in the process of apoptosis. Bax is pro-apoptotic, whereas Bcl-2 is anti-apoptotic. The ratio of these proteins within the cell is of great significance. By evaluating the Bax/Bcl-2 ratios collectively, we were able to ascertain the impact of the candidate compounds. Upon examination of the Bax/Bcl-2 ratio, it was observed that the **3i**,** 3j** and **3l** compounds exhibited efficacy in promoting mitochondrial apoptosis within the MDA-MB-231 cells at 24 h (Fig. 4). The expression of the Bax protein was found to increase significantly in the presence of the **3i**,** 3j** and **3l** compounds (*p* < 0.001) (Fig. 4a). The expression of Bcl-2 protein was found to decrease significantly in the presence of compounds **3i**,** 3j** and **3l**, with the most significant decrease observed in the case of compound **3i** (Fig. 4b; *p* < 0.001). The Bax/Bcl-2 ratio exhibited a more than fourfold increase in the compound **3i**,** 3j** and **3l** groups relative to the control group (Fig. 4c, *p* < 0.001).

**Fig. 5:**
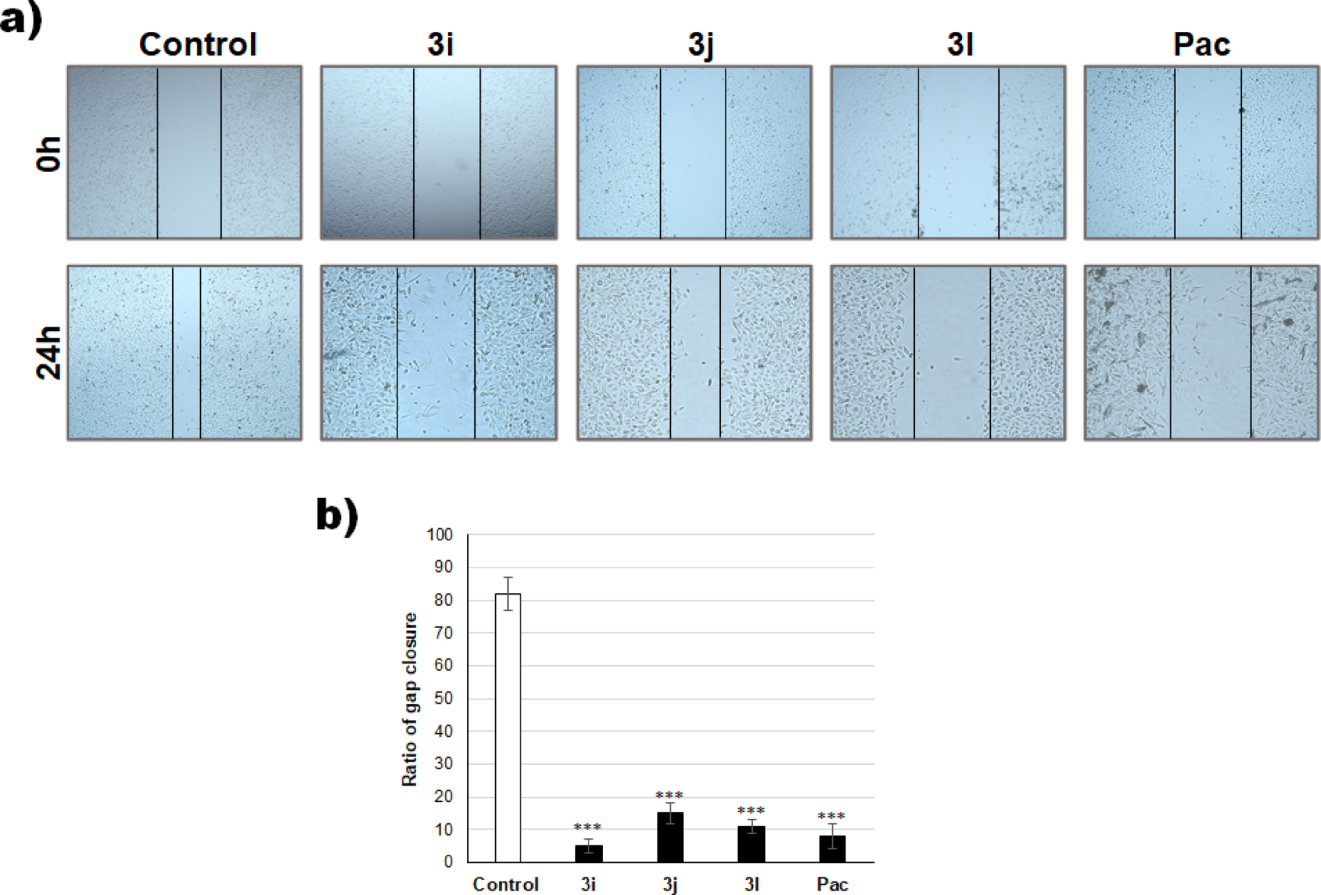
(**a**) Evaluation of the effects of the target compounds on cell migration using the scratch wound healing assay in MDA-MB-231 cells. Cells were treated with the compounds at their IC₅₀ concentrations (µM) and monitored for 24 h. (**a**) Representative images of the scratch assay showing wound closure in MDA-MB-231 cells after 24 h of treatment. (**b**) Quantitative analysis of gap closure ratios following 24 h of treatment. The wound width was measured from three different points along the scratch area, and the values were averaged for analysis. All experiments were performed in triplicate (*n* = 3), and the data are presented as mean ± SD.

Scratch assay is a basic test for cell migration and interactions with each other during the extracellular mesenchymal transition. In the experiment to assess the closure of gaps opened in MDA-MB-231 cells with the application of **3i**,** 3j**, and **3l** compounds, gap closure was measured after 24 h. In the control group cells, the closure was 82%; in the **3i**,** 3j**, and **3l** applications, it was 5%, 11%, and 15%, respectively. The gap closure percentage for paclitaxel was 8%, and the decrease in closures compared to the control group was significant in all groups (Fig. 5b, *p* < 0.001).

### METAP2 enzyme activity assay

In cancer cells, increased protein synthesis associated with rapid proliferation requires the critical activity of methionine aminopeptidase 2 (MetAP2). MetAP2 catalyzes the removal of the N-terminal methionine from newly synthesized proteins, a process that is essential for proper protein folding, structural stability, and subsequent post-translational modifications. Due to the heightened demand for protein synthesis in cancer, this process occurs at an accelerated rate^70,72^. Retention of the N-terminal methionine promotes rapid protein degradation and directly shortens protein half-life; therefore, MetAP2 is highly expressed in cancer cells to maintain protein stability and sustain malignant growth^73^. Inhibition of MetAP2 leads to cell cycle arrest at the G1 phase, followed by the induction of cell death^74–76^. Accordingly, the observed relationship between MetAP2 inhibition and cancer cell death in this study is mechanistically grounded in this biological framework.

**Table 5:** Inhibition results of MetAP2 enzyme.

Compounds	% Inhibition of MetAP2 (at 100 µM)
**3i**	51.86 ± 1.08
**3j**	26.45 ± 1.76
**3l**	44.38 ± 2.34

Fluorogenic substrate Met-AMC cleavage assays demonstrated that the synthesized compounds inhibited MetAP2 enzyme at 100 µM (Table 5). Among the tested derivatives, compounds **3i** and **3l** exhibited the highest inhibitory activity, indicating stronger binding to the enzyme’s catalytic site than the **3j** analogues. The differences in MetAP2 inhibition levels observed among the three apoptosis-inducing compounds may be attributed to variations in their binding regions on the enzyme. These MetAP2-inhibiting compounds are therefore suggested to modulate cellular signaling pathways, thereby exerting biological activity in MDA-MB-231 cells.

To further elucidate the molecular basis underlying these differences in MetAP2 inhibition, a structure–activity relationship (SAR) analysis is therefore undertaken, focusing on the most active compounds (**3i** and **3l**).

**Fig. 6:**
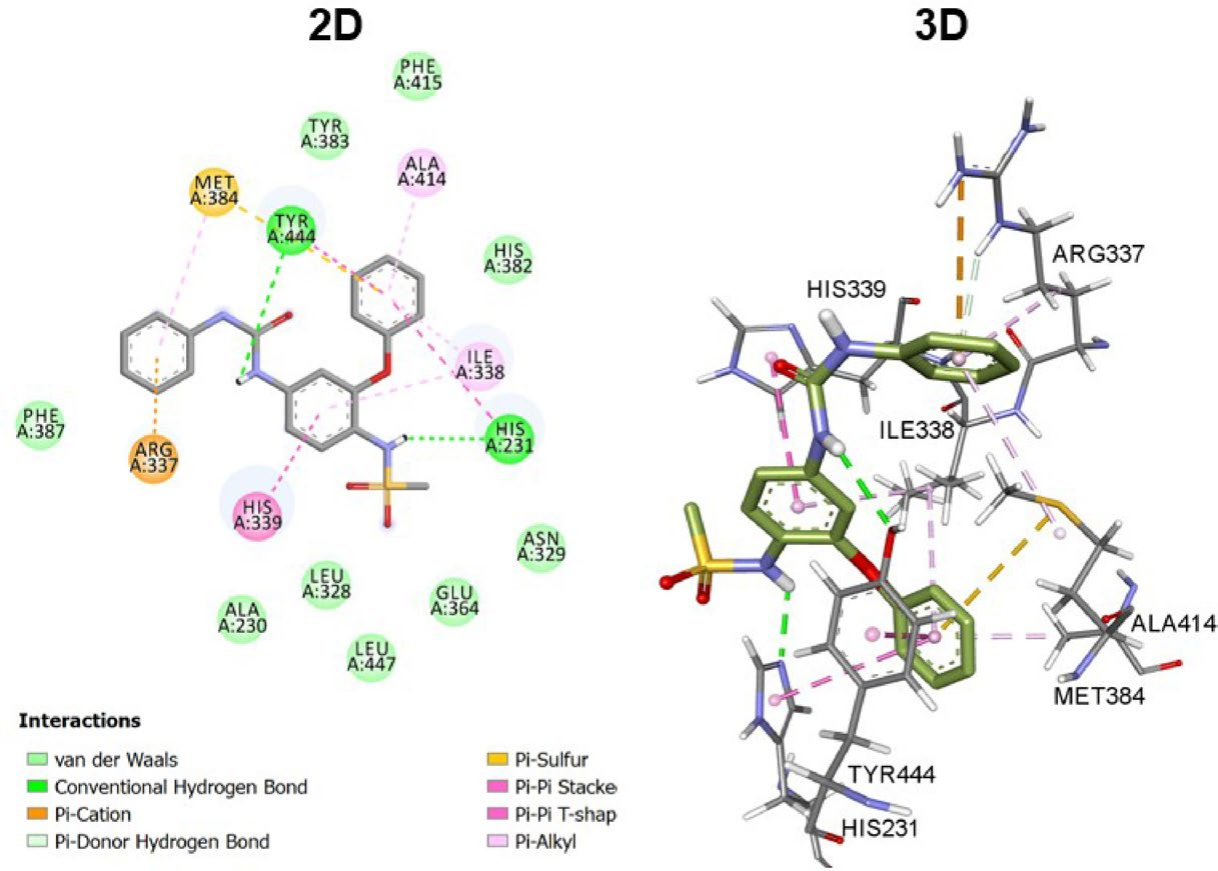
2D interaction network and 3D binding conformation of the compound **3i** docked to the MetAP2 active site, highlighting key residue interactions.

In compound **3i**, the phenyl group directly attached to the urea nitrogen, owing to its aromatic nature, enables the formation of π–π stacking and hydrophobic interactions with aromatic residues such as PHE, TYR, and HIS located within the MetAP2 active site (Fig. 6; Table 6). This planar, rigid structural feature is predicted to improve ligand accommodation within the active site, leading to reduced binding energy and enhanced inhibitory activity (Figs. 9 and 12).

**Table 6:** The binding affinity of Nimesulide urea derivatives against the MetAP2, along with the interacting residue, and the type of interaction.

Compound	Docking score (kcal/mol)	Interacting residues	Type of interactions
**3i**	− 8.0	TYR444	Conventional hydrogen bond
		TYR383	van der Waals
		PHE415	van der Waals
		ALA414	Pi-Pi Stacked
		HIS382	van der Waals
		ILE338	Pi-Alkyl
		HIS231	Conventional hydrogen bond
		ASN329	van der Waals
		GLU364	van der Waals
		LEU447	van der Waals
		LEU328	van der Waals
		ALA230	van der Waals
		HIS339	Pi–Pi stacked
		ARG337	Pi-Cation
		PHE387	van der Waals
**3j**	− 7.9	TYR444	Conventional hydrogen bond
		ILE338	Pi-Alkyl
		MET384	van der Waals
		ARG337	van der Waals
		HIS339	Conventional hydrogen bond
		LEU328	van der Waals
		ASN329	Conventional hydrogen bond
		ASP251	van der Waals
		GLN457	van der Waals
		ASP262	van der Waals
		HIS331	van der Waals
		GLU459	van der Waals
		GLU364	Co van der Waals
		PHE219	van der Waals
		LEU447	van der Waals
		HIS231	Pi-Alkyl
		PRO220	van der Waals
		HIS382	van der Waals
		ALA414	Pi–Pi stacked
**3l**	− 7.8	LEU447	van der Waals
		HIS339	Conventional hydrogen bond
		TYR444	van der Waals
		ILE338	Pi-Alkyl
		MET384	Pi-Sulfur
		ALA414	Pi-Alkyl
		PHE219	van der Waals
		HIS231	van der Waals
		ASP251	van der Waals
		PRO220	van der Waals
		GLY222	van der Waals
		GLN457	van der Waals
		HIS382	Pi-Sulfur
		ALA230	Pi-Alkyl
		ASN329	van der Waals
		GLU364	Conventional hydrogen bond
		HIS331	van der Waals

In compound **3l**, the presence of a methylene linker in addition to the phenyl ring within the benzyl group confers greater conformational flexibility to the R substituent. This flexibility allows the ligand to adapt to different binding orientations within the active site, thereby facilitating π–alkyl, π–π-sulfur, and van der Waals interactions (Fig. 8; Table 6).

The ability of both compounds to effectively engage in hydrophobic and aromatic interactions within the METAP-2 active site is consistent with the formation of more stable complexes observed in the RMSD and RMSF analyses (Figs. 9 and 11). This observation suggests an increase in the dynamic stability of the protein–ligand complex and a more effective suppression of METAP-2’s catalytic activity. These structural features are therefore predicted to contribute to the induction of cancer cell death.

### Molecular modeling and docking studies

The X-ray crystallographic structure of human methionine aminopeptidase2 (PDB ID: 5CLS) complexed with spiroepoxytriazole inhibitor with a resolution of 1.75 Å was retrieved from the protein data bank website (https://www.rcsb.org/). The detailed procedures for ligand and enzyme preparations were reported elsewhere^22^.

As shown in Table 7, the computational docking results revealed that all three compounds, **3i**, **3j**, and **3l**, are promising candidates with favorable binding energies and inhibition constants. **3i**,** 3j**, and **3l** show approximately the same binding affinities, with binding energies of − 8.0, − 7.9, and − 7.8 kcal/mol and Ki values of 0.737, 1.50, and 1.73 µM, respectively, indicating strong potential to bind and inhibit the MetAP2 enzyme effectively.

**Table 7:** Calculated molecular docking scores (ΔG, kcal/mol) and inhibition constants (K_i_, µM) of compounds **3a–l**.

Comp. No	Metap2 protein	
	Free energy of binding (kcal/mol)	Inhibition constant (Ki, µM)
**3a**	− 7.1	5.59
**3b**	− 6.7	12.48
**3c**	− 7.0	6.53
**3d**	− 8.2	0.968
**3e**	− 8.0	1.30
**3f**	− 8.1	1.12
**3g**	− 8.0	1.30
**3h**	− 8.0	1.30
**3i**	− 8.0	0.737
**3j**	− 7.9	1.50
**3k**	− 8.1	1.12
**3l**	− 7.8	1.73

2D and 3D docking pictures are valuable for understanding preferred binding interactions and predicting binding affinities of the 12 ligands. Such that 2D diagrams reveal the general orientation of the ligand within the binding site through key residues with different interaction types. In contrast, in 3D diagrams, we can visualize the ligand’s 3D conformation with precise atom orientations and distances within the binding pocket, providing a more in-depth understanding.

**Fig. 7:**
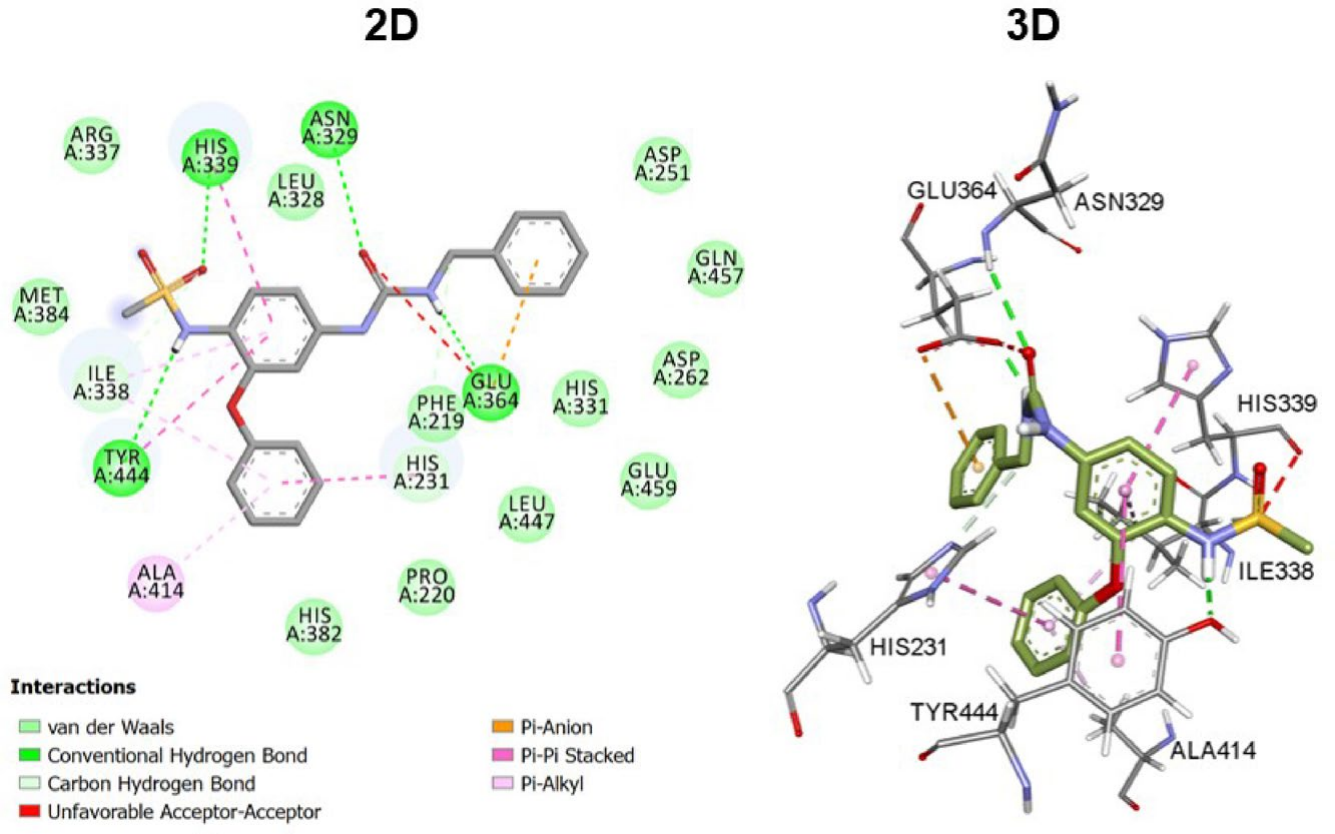
2D interaction network and 3D binding conformation of the compound **3j** docked to the MetAP2 active site, highlighting key residue interactions.

**Fig. 8:**
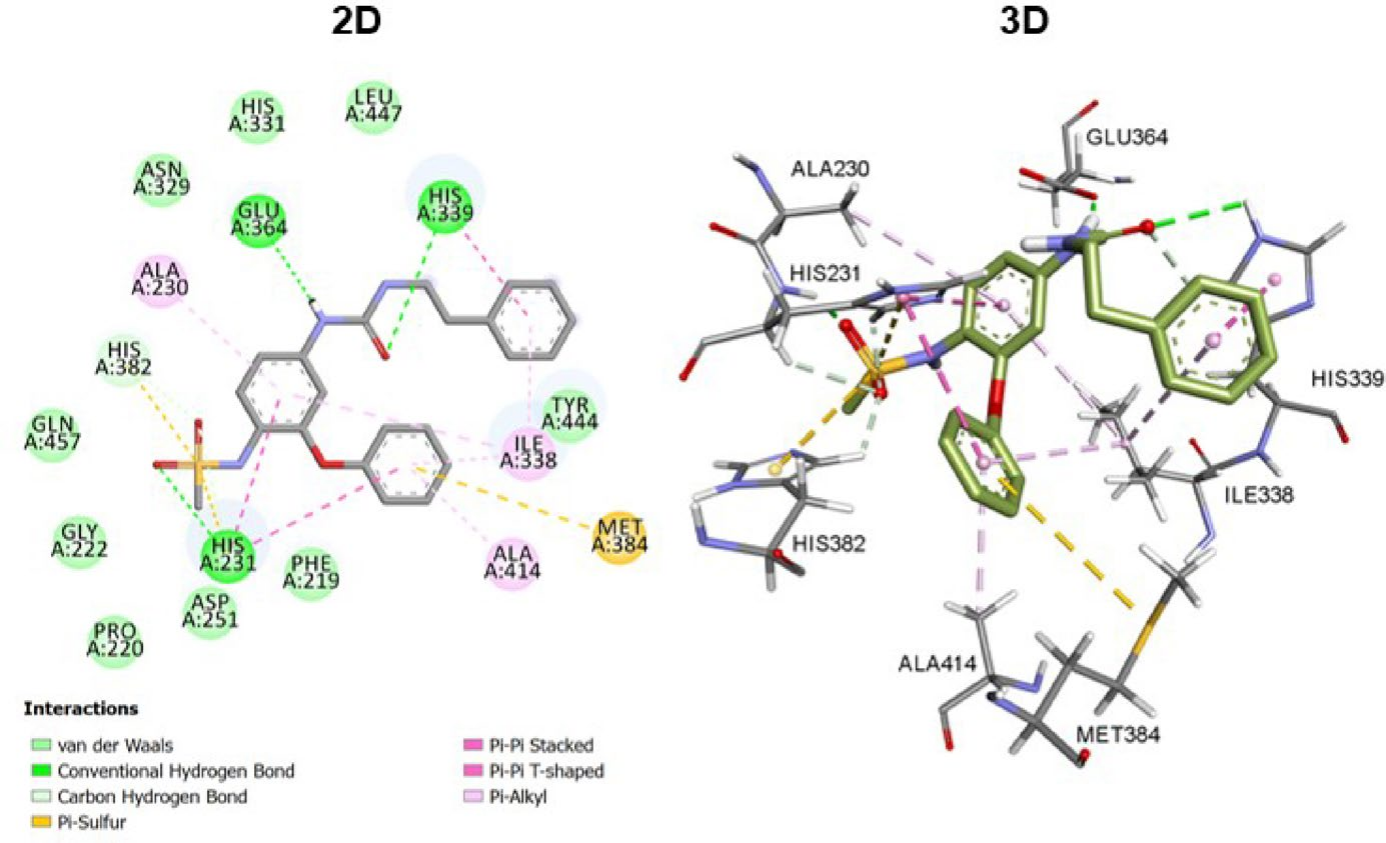
2D interaction network and 3D binding conformation of the compound **3l** docked to the MetAP2 active site, highlighting key residue interactions.

**3i**, **3j**, and **3l** form multiple favorable interactions with key residues in the active site of MetAP2, including hydrogen bonds with HIS231, HIS382, and ASP262, as well as stabilizing hydrophobic contacts involving LEU213 and TYR232. These interaction patterns suggest that the **3i**, **3j**, and **3l** compounds are well-positioned within the enzyme’s binding pocket, contributing to their high inhibitory activity. The 2D and 3D binding poses of **3i**, **3j**, and **3l** in complex with MetAP2 through specific residue interactions are shown in Figs. 6, 7 and 8 in detail.

RMSD plots assessed overall structural deviations over a 200 ns simulation time, while RMSF profiles evaluated residue-specific flexibility. The comparative analysis focused on structural stability, ligand-induced stabilization, and conformational dynamics relevant to inhibitory efficacy.

**Fig. 9:**
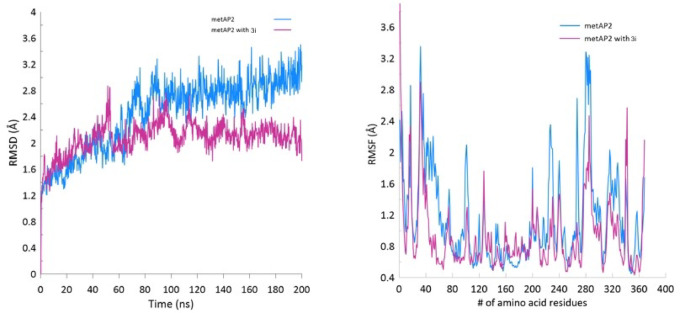
RMSD (left) and RMSF (right) graphs indicating the conformational stability and residue-specific flexibility of apo-MetAP2 and **MetAP2-3i** complexes throughout a 200 ns simulation.

**Fig. 10:**
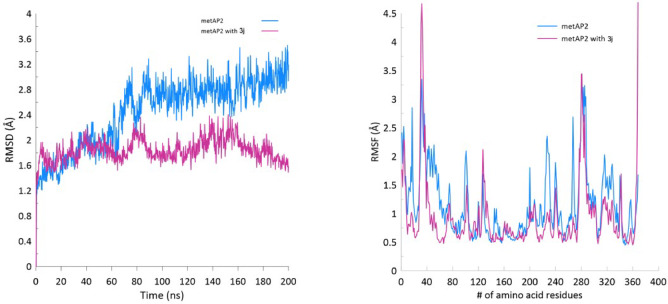
RMSD (left) and RMSF (right) graphs indicating the conformational stability and residue-specific flexibility of apo-MetAP2 and **MetAP2-3j** complexes throughout a 200 ns simulation.

**MetAP2-3i**, **MetAP2-3j**, and **MetAP2-3l** complexes were selected for their most coherent experimental and computational results. The root mean square deviation (RMSD) and root mean square fluctuation (RMSF) graphs for the complexes are shown in Figs. 9 and 10, respectively. The graphs demonstrate that the ligands (**3i**, **3j**, and **3l**) significantly stabilize the enzyme compared to the apo-form, exhibit slightly superior stabilizing properties compared to other candidates, and show strong inhibitory potential.

The apo-MetAP2 displayed higher RMSD values (~ 2.4–3.2 Å), indicating greater conformational flexibility and dynamic behavior without ligands. Nevertheless, MetAP2 with **3i**, **3j**, and **3l** exhibits lower average RMSD values (~ 1.6–2.3 Å) as a complex relative to the apo-form, indicating enhanced structural stability. Among them, **3j** and **3l** show the greatest stabilization with an RMSD peak around 1.8 Å, while **3i** is comparable at approximately 2.0 Å.

Residue-wise RMSF profiles reveal that flexible regions spanning the amino acid residues 0–70, 230–250, and 270–300 in apo-MetAP2 fluctuate up to 3.6 Å, particularly in loop regions and termini. On the other hand, ligand-bound forms reduce these elevated fluctuations in those regions to approximately 1.2–1.8 Å. All three complexes exhibit a pronounced decrease in local flexibility in these key regions. These findings suggest that **3i**, **3j**, and **3l** ligands effectively constrain protein dynamics, particularly near the active site, consistent with their experimentally confirmed potent inhibitory effects.

**Fig. 11:**
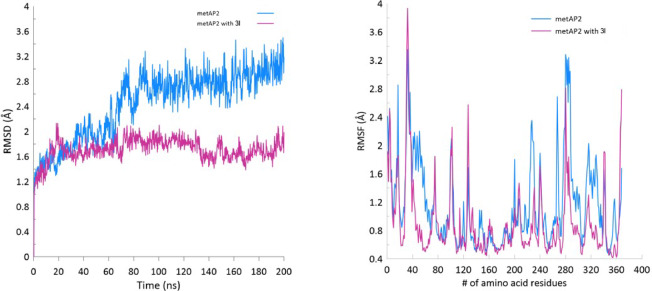
RMSD (left) and RMSF (right) graphs indicating the conformational stability and residue-specific flexibility of apo-MetAP2 and **MetAP2-3l** complexes throughout a 200 ns simulation.

**Fig. 12:**
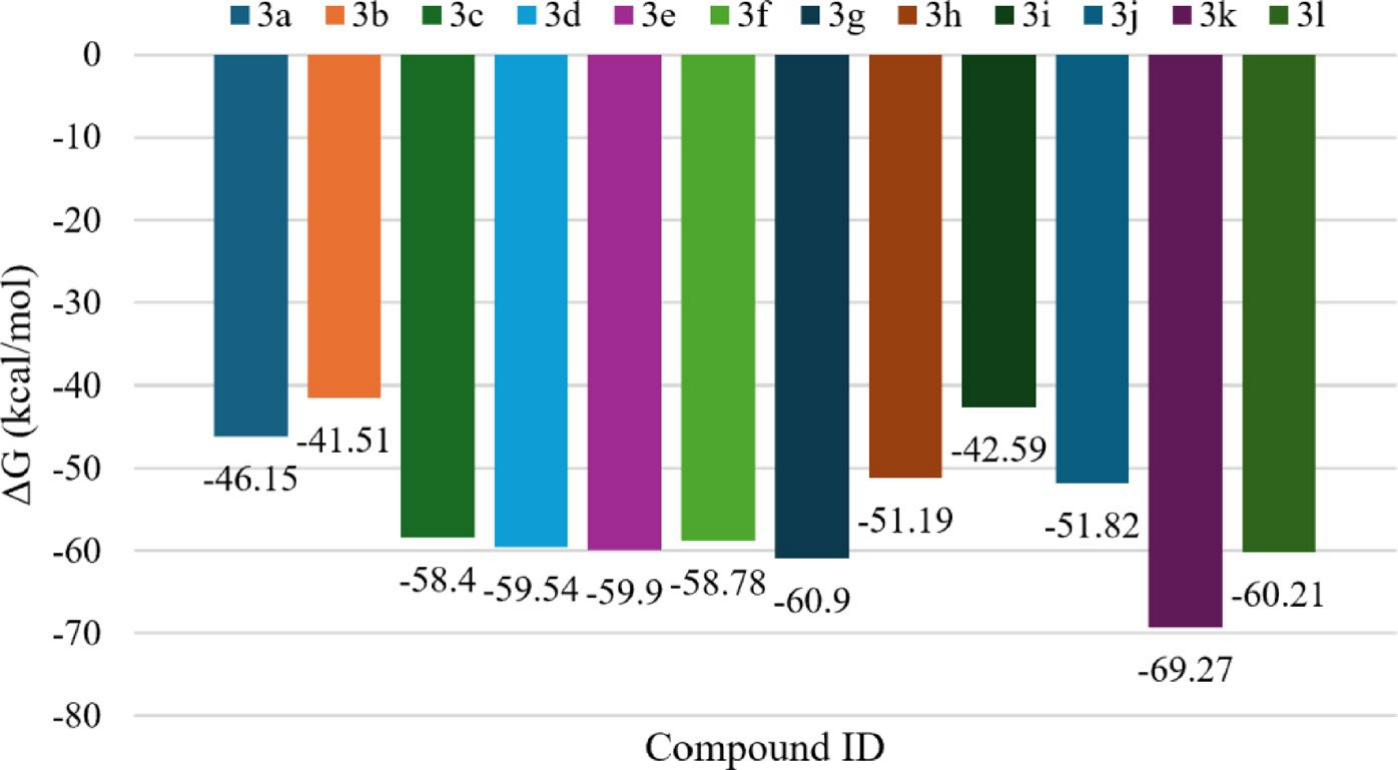
The average MM/GBSA binding energies of the 12 compounds (**3a-l**) are in complex with the MetAP2 enzyme throughout 200 ns MD simulations.

The binding free energy (ΔG) obtained through MM/GBSA calculations is critical in evaluating ligand efficacy. When combined with experimental inhibition data (IC_50_), these computational results can validate the potential of these ligands as effective inhibitors. A more negative ΔG indicates stronger binding, often correlating with stable interactions and higher inhibitory potency. In Fig. 12, MM/GBSA binding energy calculations were displayed for the 12 compounds (**3a-l**) as the average binding free energy (ΔG) values.

As their binding positions, the ligands **3i**, **3j**, and **3l** show multiple key hydrogen bonds and hydrophobic interactions with critical residues in MetAP2’s active site. These favorable binding modes strongly support their efficacy as inhibitors, aligning well with their confirmed potent inhibitory activities in vitro, thereby validating the computational findings as consistent and predictive of experimental results.

Afterwards, the study provides compelling molecular dynamics outcomes indicating that ligands **3i**, **3j**, and **3l** can structurally and effectively stabilize the MetAP2 enzyme, as evidenced by lower RMSD and RMSF values. Further in vivo assays are warranted to confirm their inhibitory activity as potent inhibitors and to assess the therapeutic potential of these ligands, which show promise as anti-cancer or anti-angiogenic agents targeting the MetAP2 enzyme. In addition, to assess potential selectivity toward COX enzymes, additional docking analyses were carried out, and the results are provided in the Supporting Information (Table S1–S2).

## Conclusion

Clinical research findings have demonstrated that non-steroidal anti-inflammatory drugs (NSAIDs) may reduce the risk of breast, lung, prostate, and colon cancers. Among NSAIDs, nimesulide has been frequently cited in the literature due to its potential antiproliferative effects on cancer cells. Compared to other NSAIDs, nimesulide is reported to have a more favorable side effect profile, particularly in terms of gastrointestinal tolerability. However, caution is advised in patients with renal or hepatic impairment, as the most notable adverse effect associated with nimesulide is hepatotoxicity. In addition, the literature frequently emphasizes that larger molecular structures such as nimesulide can enhance target selectivity by providing broader interaction surfaces with biological targets^22,33,53^.

A series of novel nimesulide-derived urea compounds (**3a–l**) were synthesized under organotin catalysis. Through structural modification, these derivatives would allow for reduced adverse effects of nimesulide and improved biological target selectivity. The structures of the synthesized compounds were characterized using chromatographic methods and various spectroscopic techniques, including^1^ H NMR, ^13^C NMR, FTIR, and HR-MS.

Nimesulide is known to exhibit antioxidant properties due to the presence of a sulfonyl sulfonanilide group in its structure^70^. Moreover, the antioxidant activities of urea derivatives are frequently emphasized in the literature. In this context, the antioxidant activities of the newly synthesized nimesulide-derived urea compounds (**3a–l**) were evaluated using the DPPH (2,2-diphenyl-1-picrylhydrazyl) radical scavenging assay. The results revealed that compound **3f**, bearing a para-substituted fluorine group, exhibited antioxidant activity comparable to the reference standards.

The anti-proliferative effects of compounds **3i**, **3j**, and **3l** at low doses in triple-negative breast cancer cells suggest that they may be candidates for cancer treatment. These compounds have been shown to suppress mechanisms that play a key role in apoptosis, thereby increasing cell death. Docking studies showed it is highly probable that inhibition of the MetAP-2 enzyme mediates this effect. Inhibition of MetAP-2, a protein highly expressed in triple-negative breast cancer cells, has been shown to slow cell division and accelerate progression toward apoptosis.

Molecular docking, MD simulations, and MM/GBSA calculations were performed to determine binding interactions and dynamic behaviour of newly designed compounds, **3i**,** 3j**, and **3l**, with the MetAP-2 enzyme. Based on the computational results obtained for newly synthesized compounds, superior docking scores of − 8.0 kcal/mol, − 7.9 kcal/mol, and − 7.8 kcal/mol support the experimental results. As a result of molecular modelling, the generated 2D and 3D poses of these inhibitor-protein complexes present invaluable insight into the binding interaction of these inhibitors with the active side residues of the Mer-tAP-2 enzyme. Results from this study can be used to develop an effective novel design for MetAP-2 inhibitors for breast cancer treatment.

Consequently, the present study substantiates that the compounds synthesized have the potential to serve as new targets and will contribute to the development of new treatments through advanced analysis in drug development.

## Electronic Supplementary Material

Below is the link to the electronic supplementary material.

Supplementary Material 1

## Data Availability

All data generated or analyzed in this study are included in the Supplementary Information file.
