# Supplementary material for: Synthesis, characterization, biological evaluation, and molecular modeling of novel nimesulide urea derivatives as potential MetAP2 inhibitors
Source: Sci Rep. 2026 Apr 24;16:19076. doi: 10.1038/s41598-026-50210-0 (PMC13280157; doi:10.1038/s41598-026-50210-0)
Supplement: Supplementary file 1 — Supplementary Material 1 [file 41598_2026_50210_MOESM1_ESM.docx]

Supporting Information

**Synthesis, Characterization, Biological Evaluation, and Molecular Modeling of Novel Nimesulide Urea Derivatives as Potential MetAP2 Inhibitors**

Özgür Yılmaz^a,b*^, Yağmur Biliz^a,c^, Elif Kuloğlu^a,d^,Kübra Arancı^a,e,f^, Ömer Erdoğan^g^, Özge Çevik^h^, Müfide Karahasanoğlu^a^, Naz Mina Mert Şahin^i^, Ayşe Buse Çakır^a^, Bilge Tuzcu^a,b,j^, Kemal Yelekçi^i^,

Ş. Güniz Küçükgüzel^k^

*^a^TUBITAK Marmara Research Center, Gebze, Kocaeli 41470, Turkiye*

*^b^Department of Pharmaceutical Chemistry, Faculty of Pharmacy, Istanbul University, Istanbul, 34116, Turkiye*

*^c^Department of Chemical Engineering, Faculty of Engineering and Natural Sciences, Istanbul Health and Technology University, Istanbul, 34275, Turkiye*

*^d^Department of Chemistry, Faculty of Art and Science, Bursa Uludağ University, Bursa, 16059 Turkiye*

*^e^Department of Bioengineering, Faculty of Engineering, Marmara University, 34854,Istanbul,* *Turkiye*

*^f^Center for Nanotechnology &amp; Biomaterials Application and Research (NBUAM), Marmara University, Turkiye*

*^g^Department of Biochemistry, School of Medicine , Gaziantep Islam Science and Technology University, Gaziantep, 27010, Turkiye*

*^h^Department of Biochemistry, School of Medicine , Aydın Adnan Menderes University, Aydın, 09010, Turkiye*

*^i^Department of Molecular Biology and Genetics, Faculty of Engineering and Natural Sciences, Kadir Has University, Istanbul 34083, Turkiye*

*^j^Department of Pharmaceutical Chemistry, Istanbul Kent University, Faculty of Pharmacy, Kagithane/Istanbul, Turkiye*

*^k^Department of Pharmaceutical Chemistry, Faculty of Pharmacy, Fenerbahçe University, Atasehir, Istanbul 34758, Turkiye*

*Corresponding authors. [yilmaz.ozgur@tubitak.gov.tr](mailto:yilmaz.ozgur@tubitak.gov.tr)

**Contents**

| FTIR, MS and NMR Spectra of synthesized compound (**2**)...................................... | S2 |
| --- | --- |
| FTIR, MS and NMR Spectra of synthesized compound (**3a-l**).................................. | S4 |
| Molecular Modeling and Docking Studies………………………………………… | S29 |

**Copies of the FTIR, HRMS, ^1^H NMR and ^13^C NMR spectra of synthesized N-(4-Amino-2-phenoxy-phenyl)-methanesulfonamide (2)**


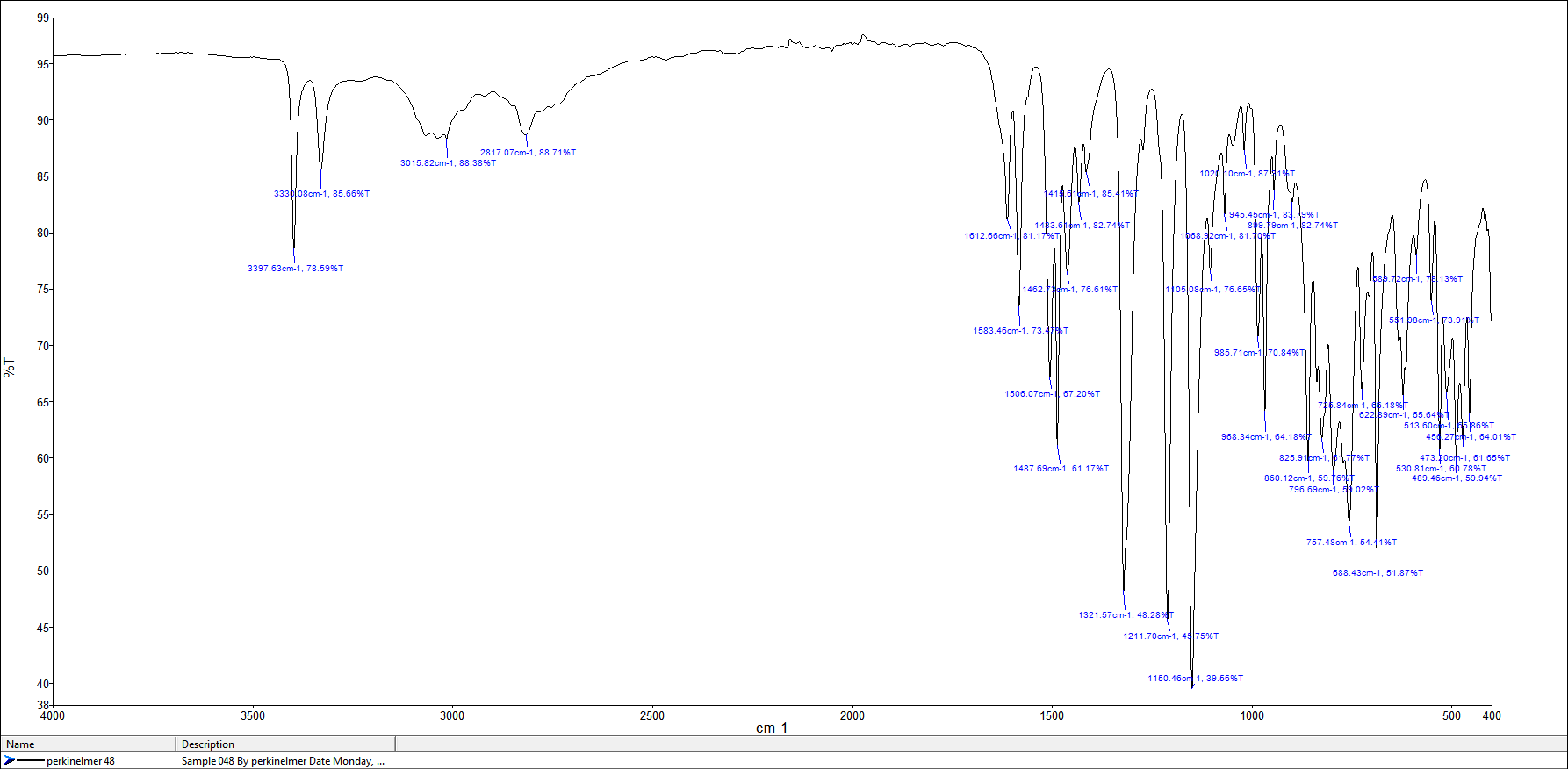


**Fig. S1**. FTIR spectrum of **2**

**
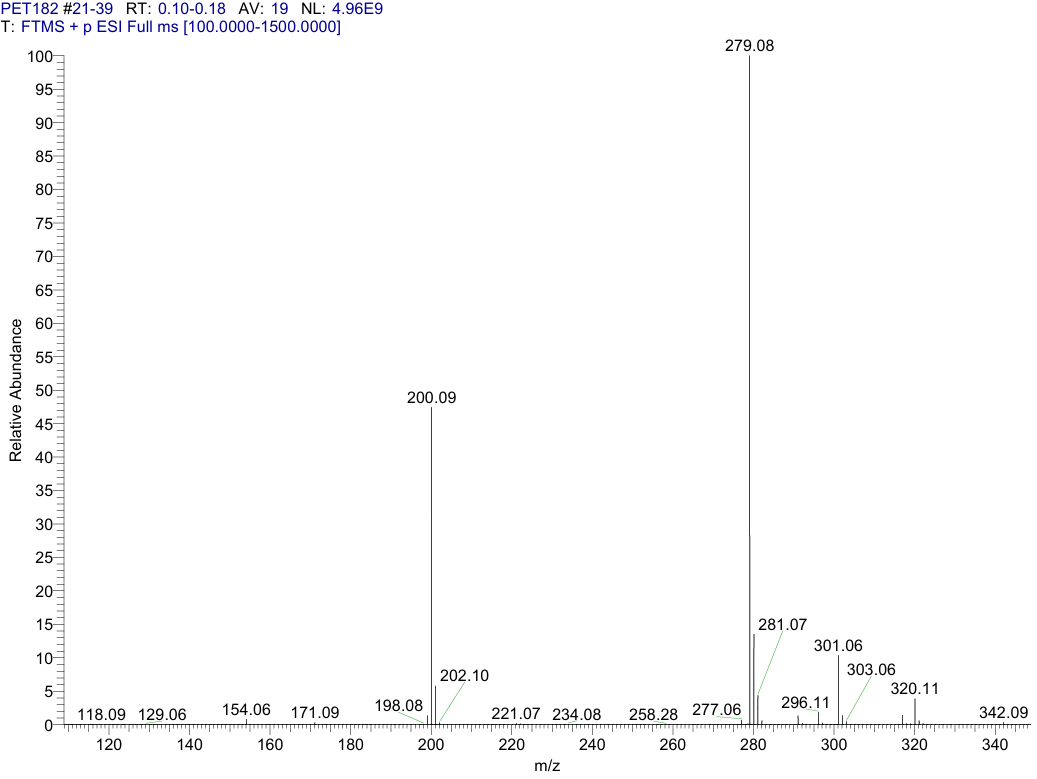
**

**Fig. S2**. HRMS (ESI-Orbitrap) spectrum of compound **2**.


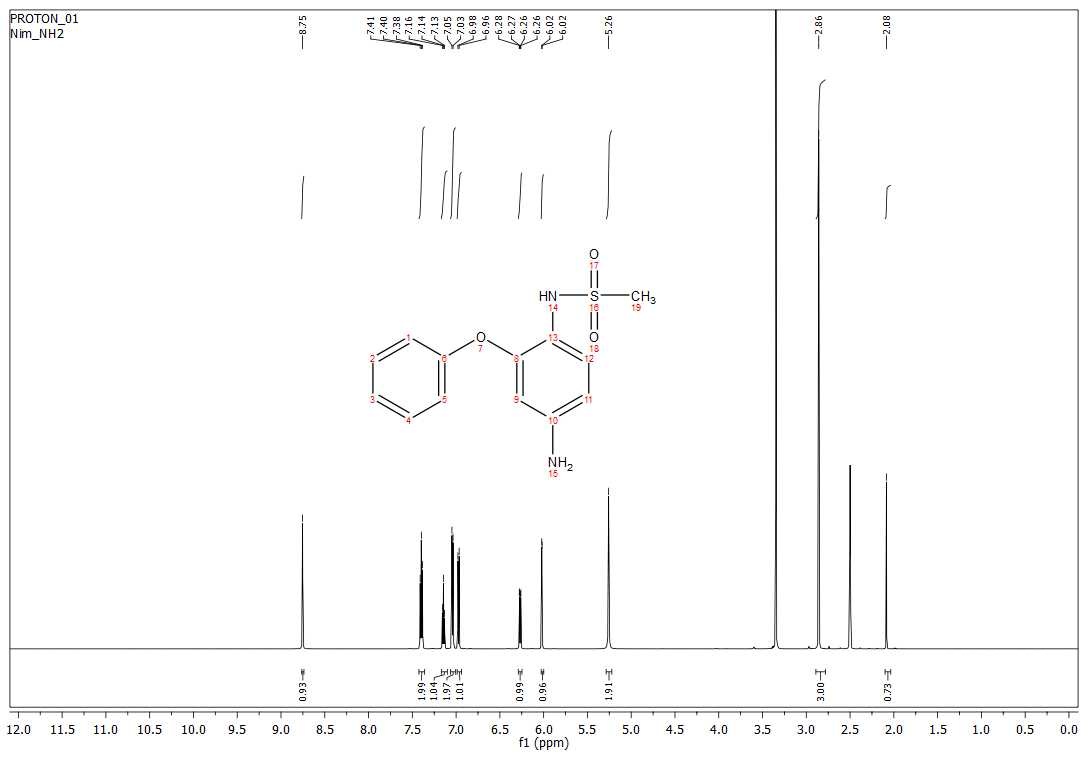


**Fig. S3**. ^1^H NMR spectrum (600 MHz, DMSO) of **2**


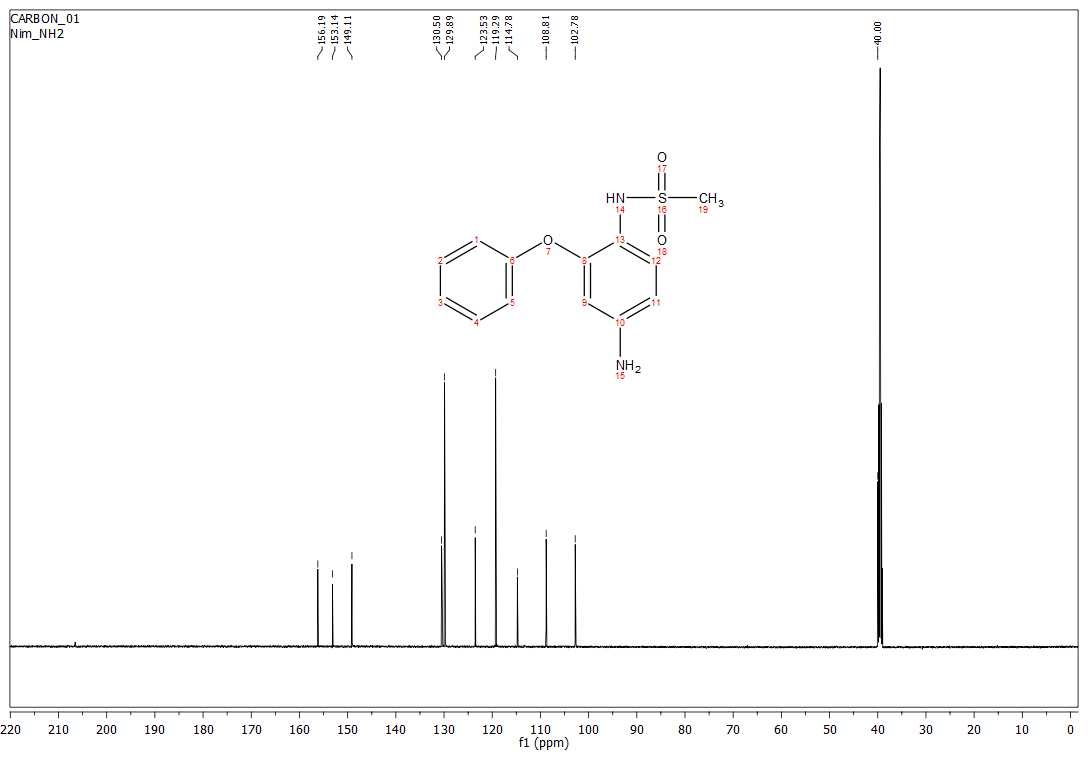


**Fig. S4**. ^13^C NMR spectrum (150 MHz, DMSO) of **2.**

**Copies of the FTIR, HRMS, ^1^H NMR and ^13^C NMR spectra of synthesized N-[4-[3-(2-Chloro-ethyl)-ureido]-2-phenoxy-phenyl]-methanesulfonamide (3a)**


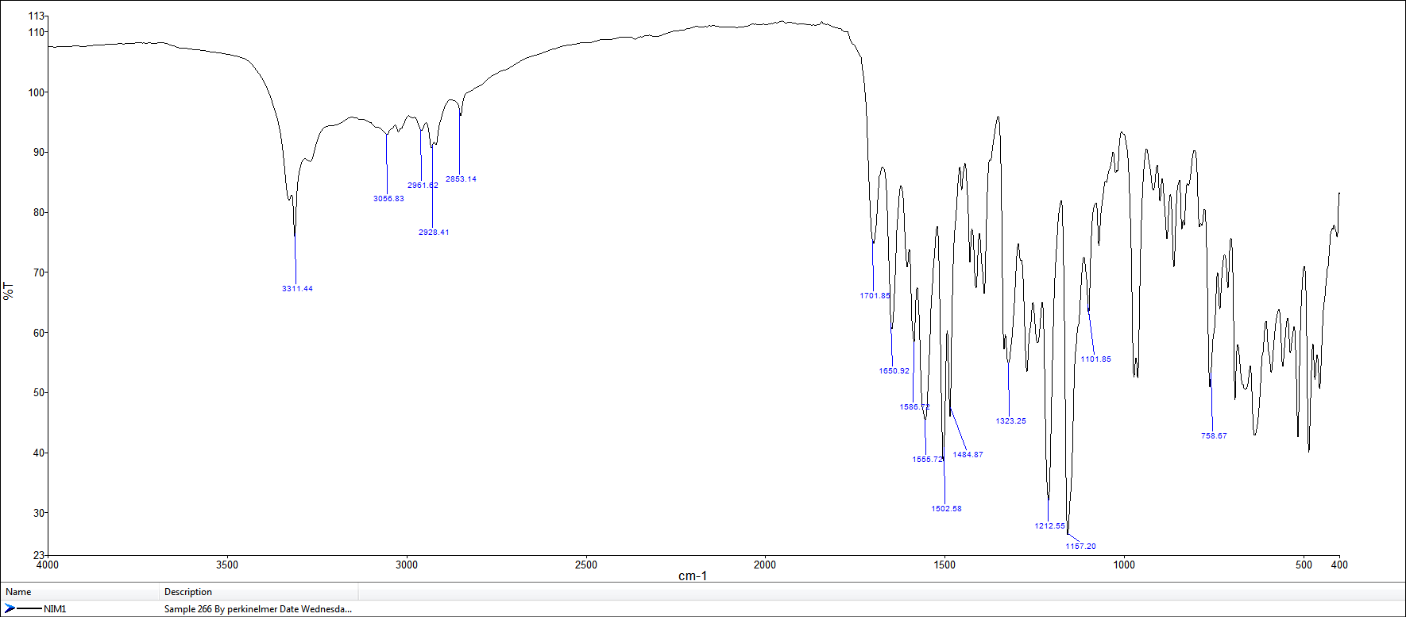


**Fig. S5**. FTIR spectrum of **3a**


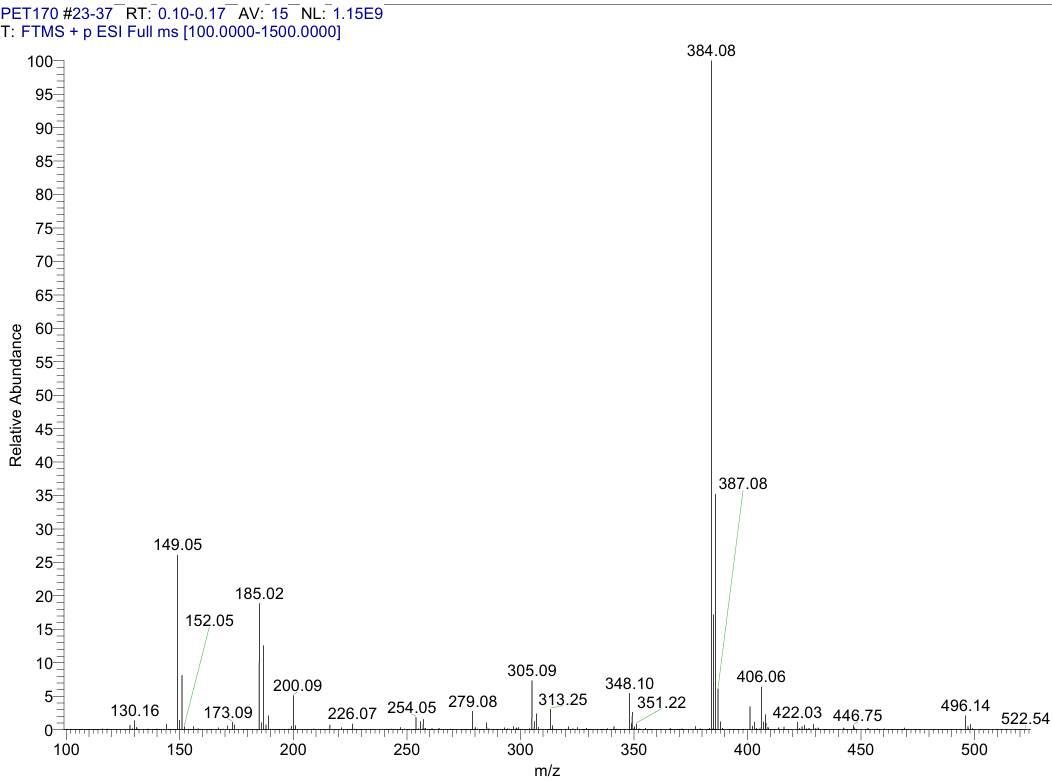


**Fig. S6**. HRMS (ESI-Orbitrap) spectrum of compound **3a.**


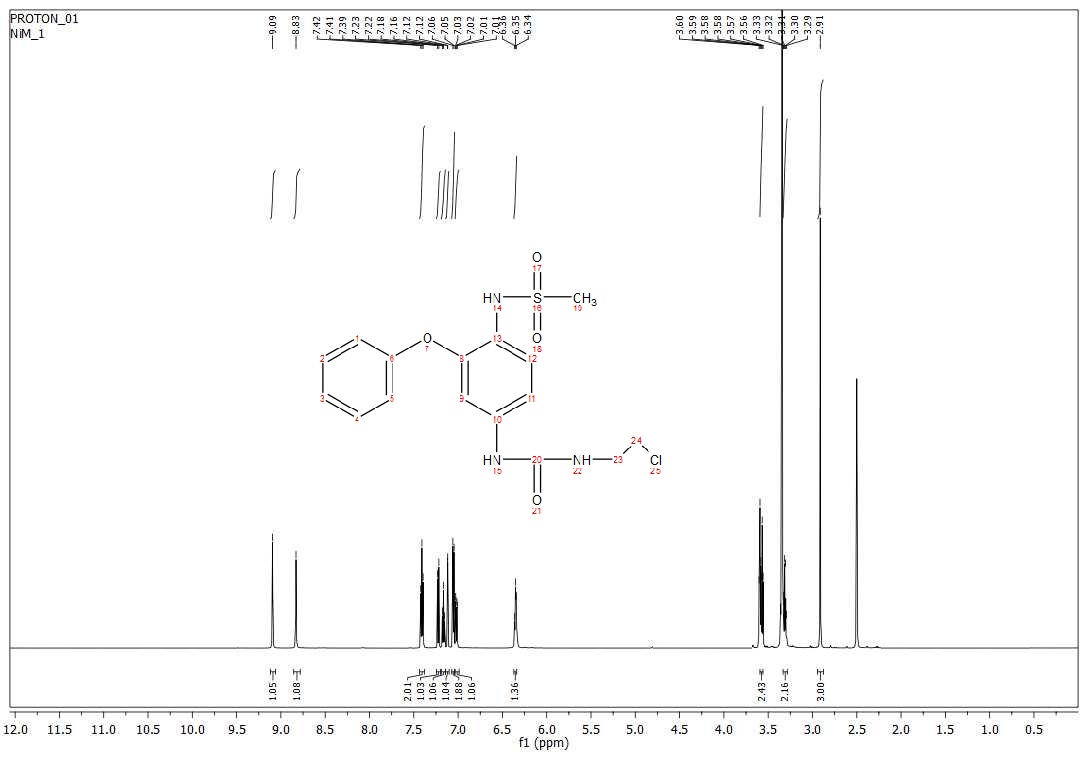


**Fig. S7**. ^1^H NMR spectrum (600 MHz, DMSO) of **3a**


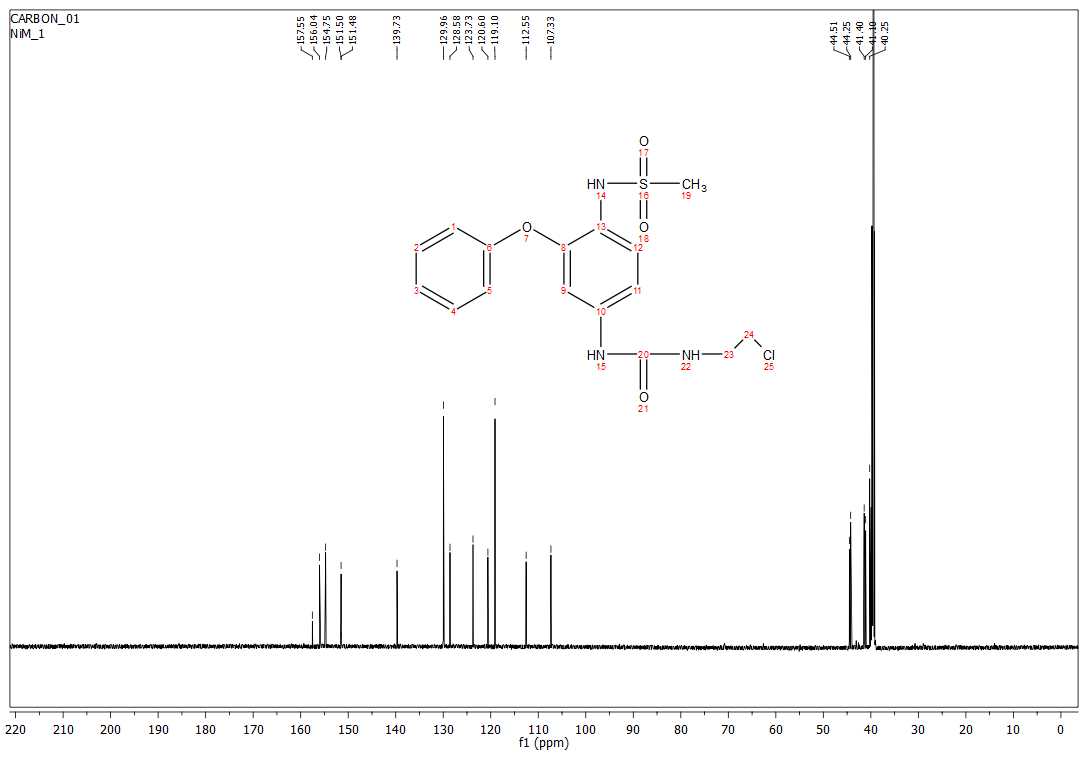


**Fig. S8**. ^13^C NMR spectrum (150 MHz, DMSO) of **3a.**

**Copies of the IR, HRMS, ^1^H NMR and ^13^C NMR spectra of synthesized N-[4-(3-Ethyl-ureido)-2-phenoxy-phenyl]-methanesulfonamide (3b)**


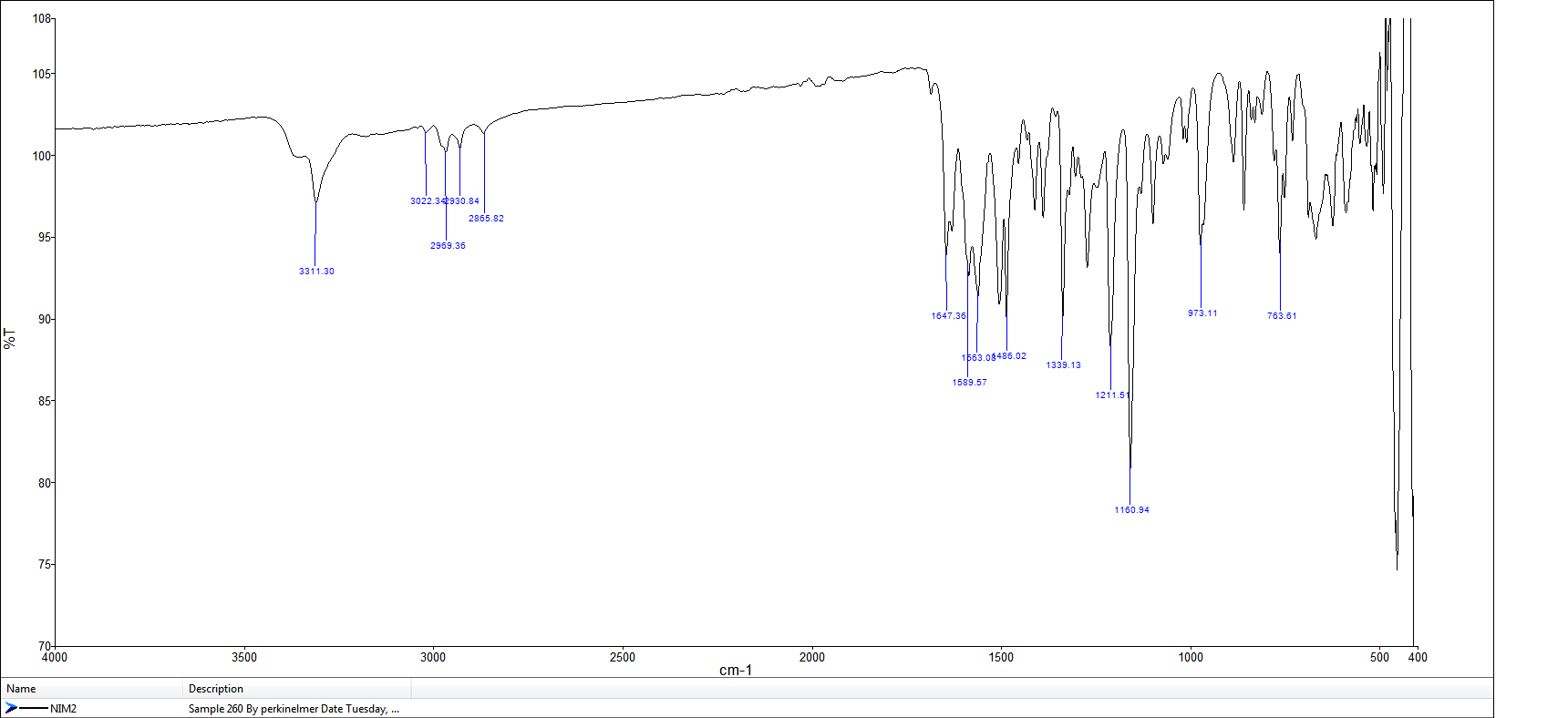


**Fig. S9**. FTIR spectrum of **3b**


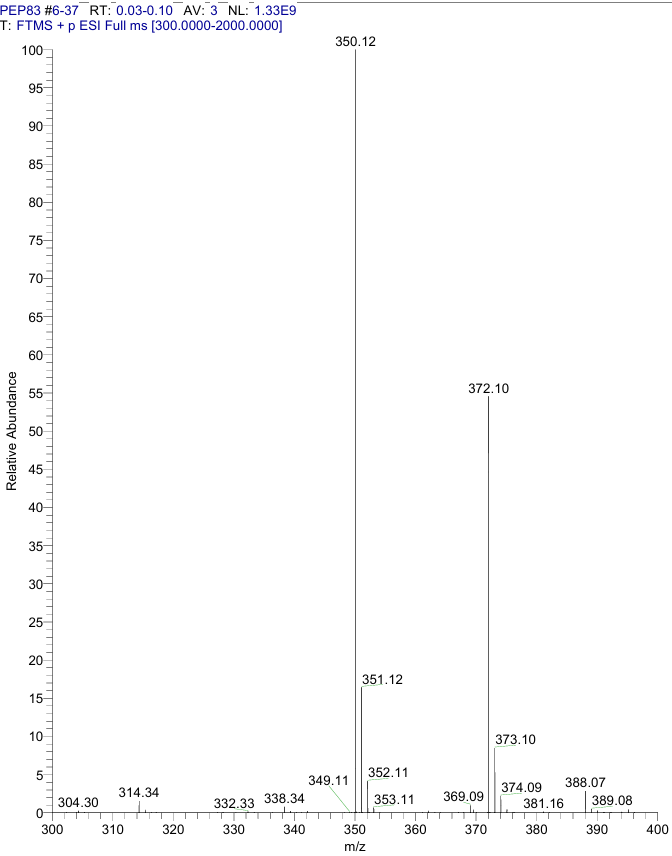


**Fig. S10**. HRMS (ESI-Orbitrap) spectrum of compound **3b**.


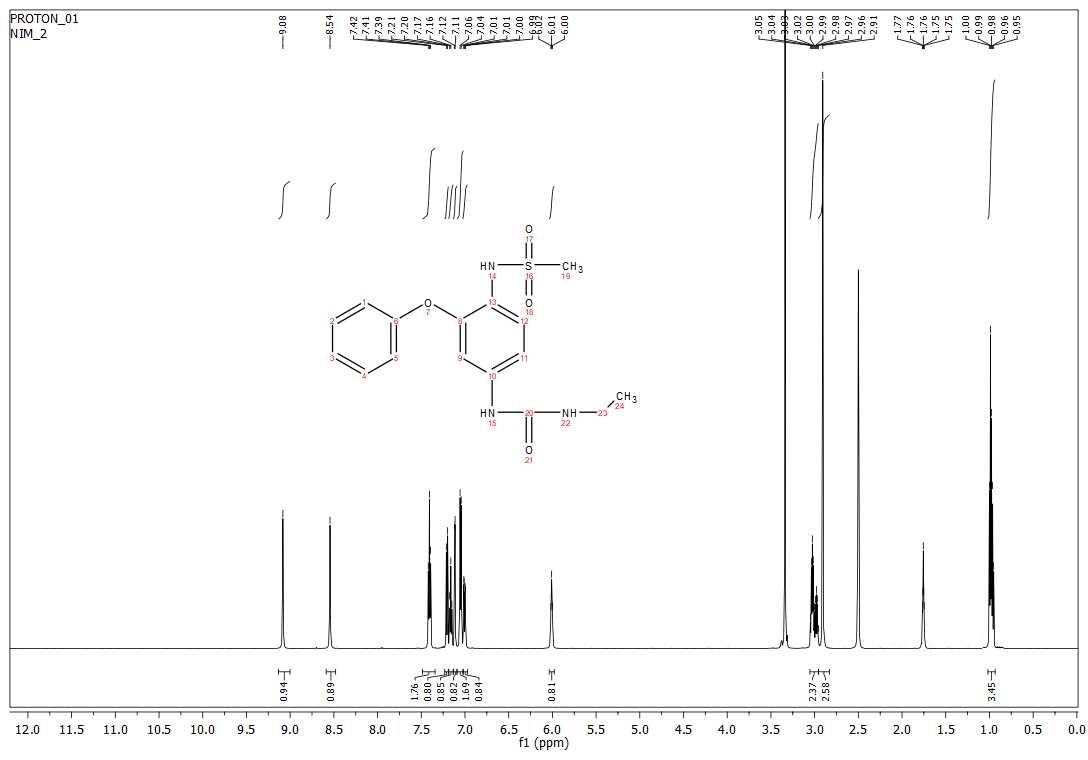


**Fig. S11**. ^1^H NMR spectrum (600 MHz, DMSO) of **3b.**


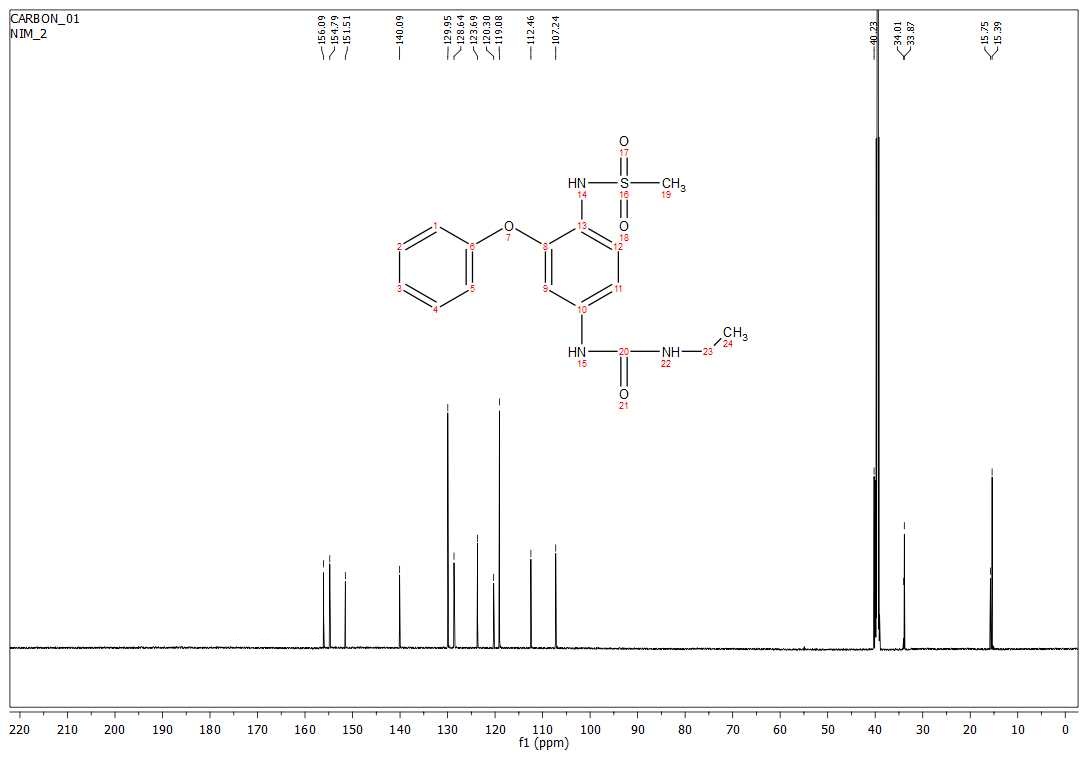


**Fig. S12**. ^13^C NMR spectrum (150 MHz, DMSO) of **3b.**

**Copies of the FTIR, HRMS, ^1^H NMR and ^13^C NMR spectra of synthesized N-[2-Phenoxy-4-(3-propyl-ureido)-phenyl]-methanesulfonamide (3c)**


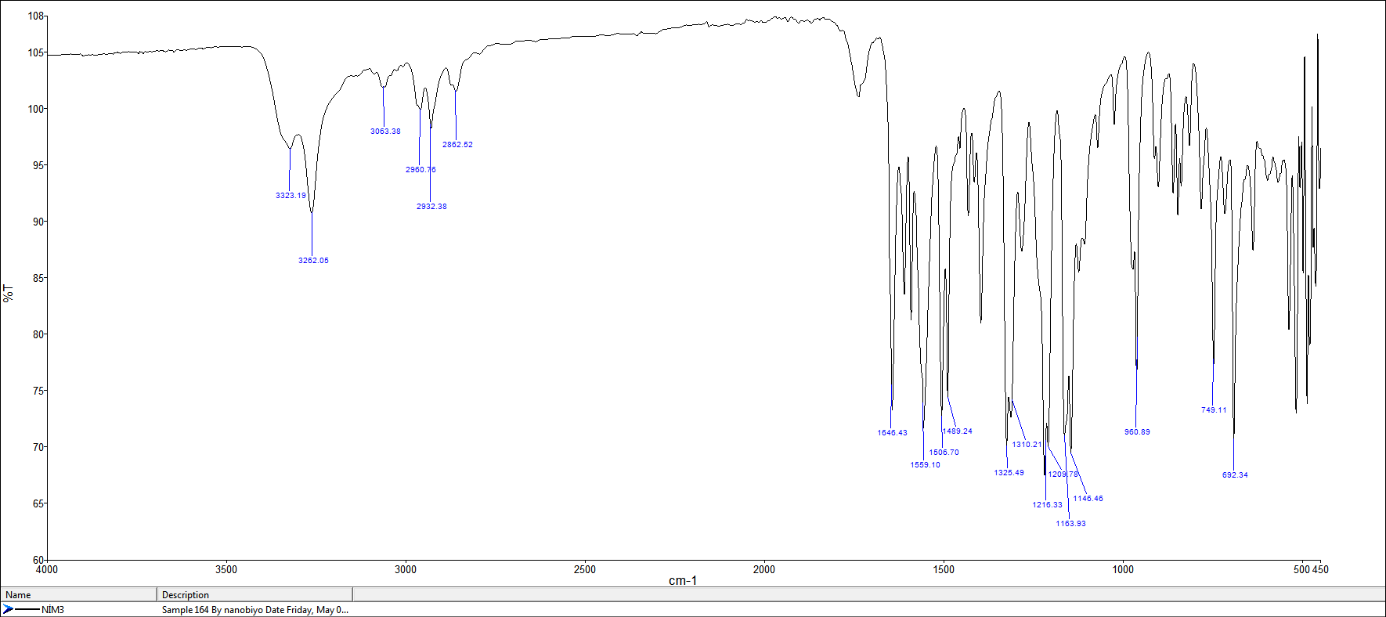


**Fig. S13**. FTIR spectrum of **3c**

**
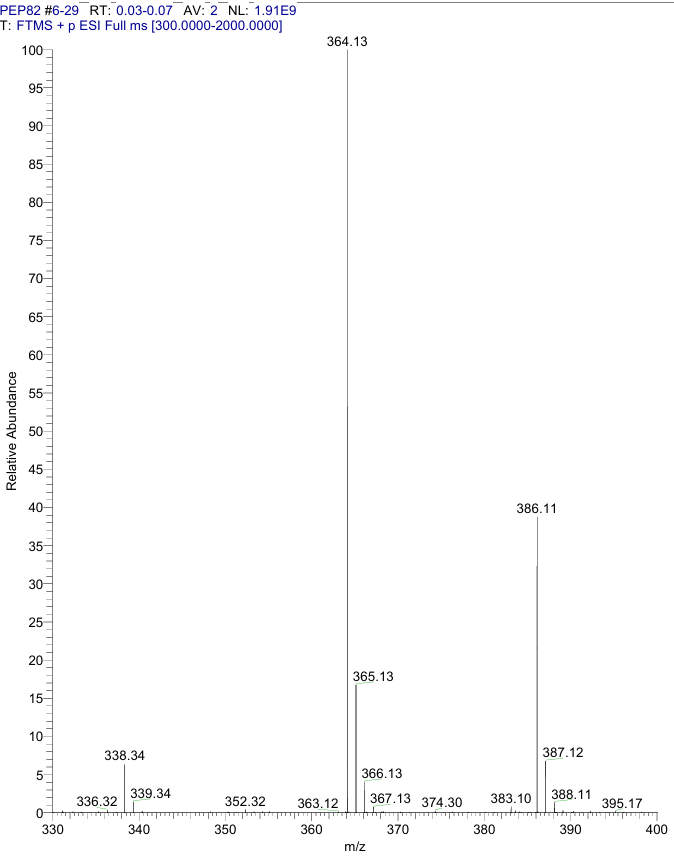
**

**Fig. S14**. HRMS (ESI-Orbitrap) spectrum of compound **3c**


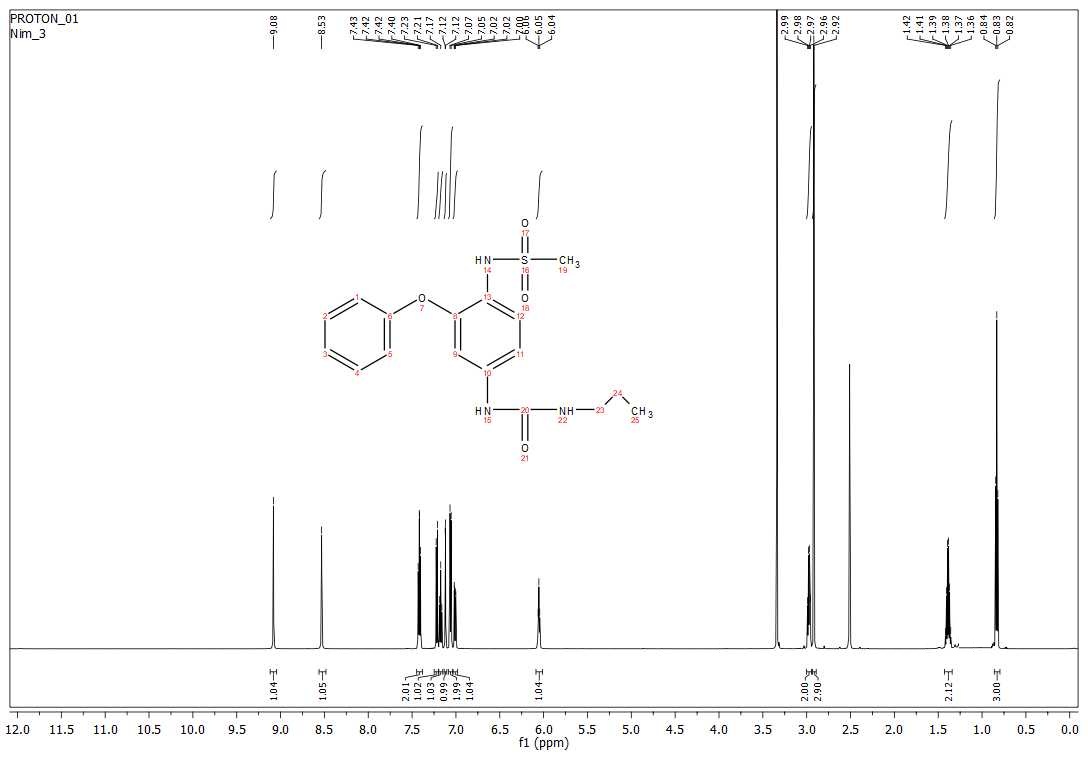


**Fig. S15**. ^1^H NMR spectrum (600 MHz, DMSO) of **3c.**


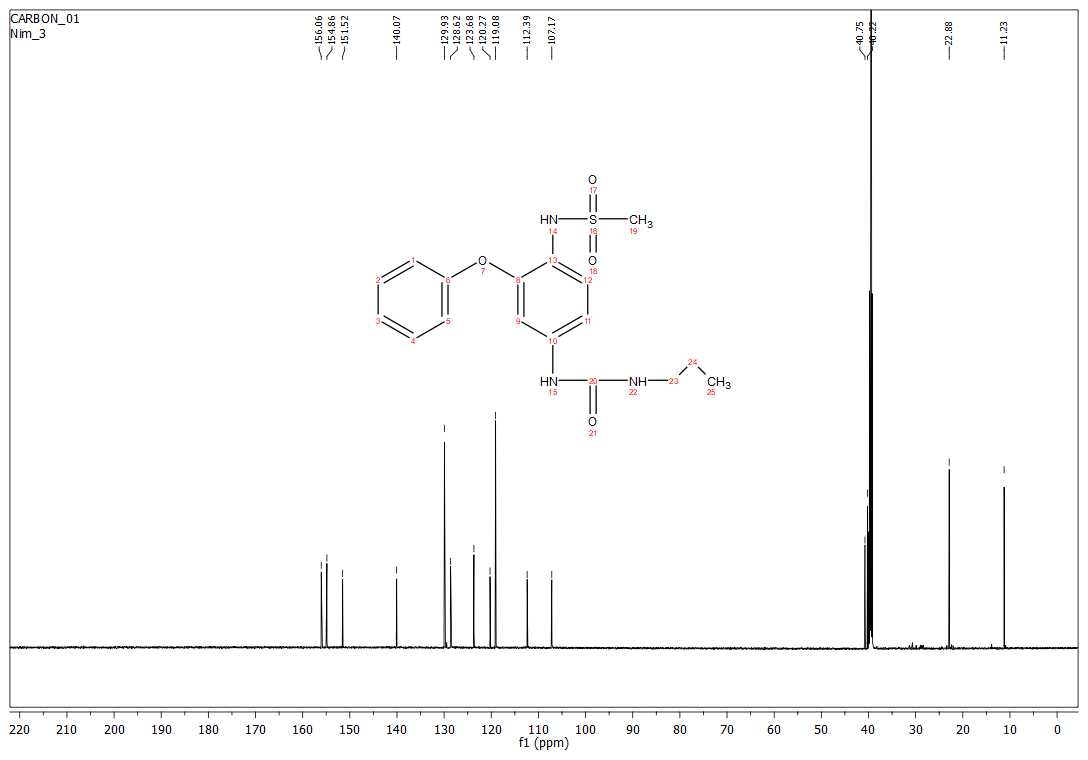


**Fig. S16**. ^13^C NMR spectrum (150 MHz, DMSO) of **3c.**


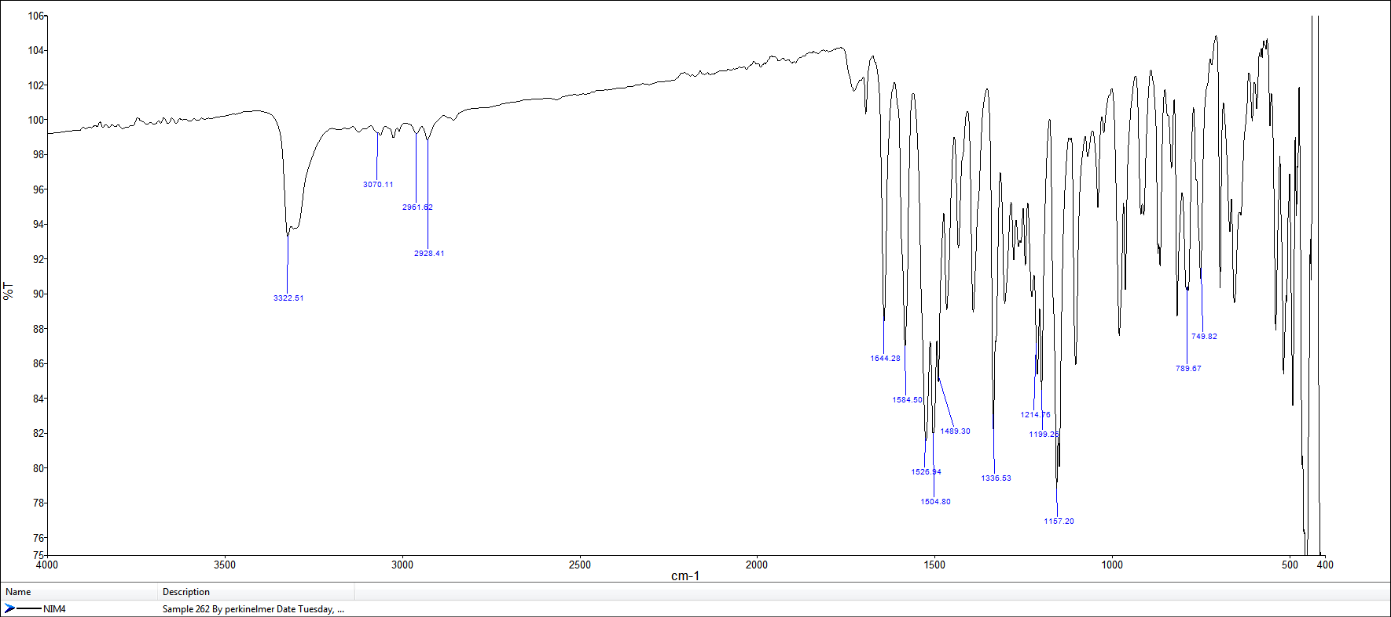
**Copies of the FTIR, HRMS, ^1^H NMR and ^13^C NMR spectra of synthesized N-[4-[3-(2,4-Dichloro-phenyl)-ureido]-2-phenoxy-phenyl]-methanesulfonamide (3d)**

**Fig. S17**. FTIR spectrum of **3d**

**
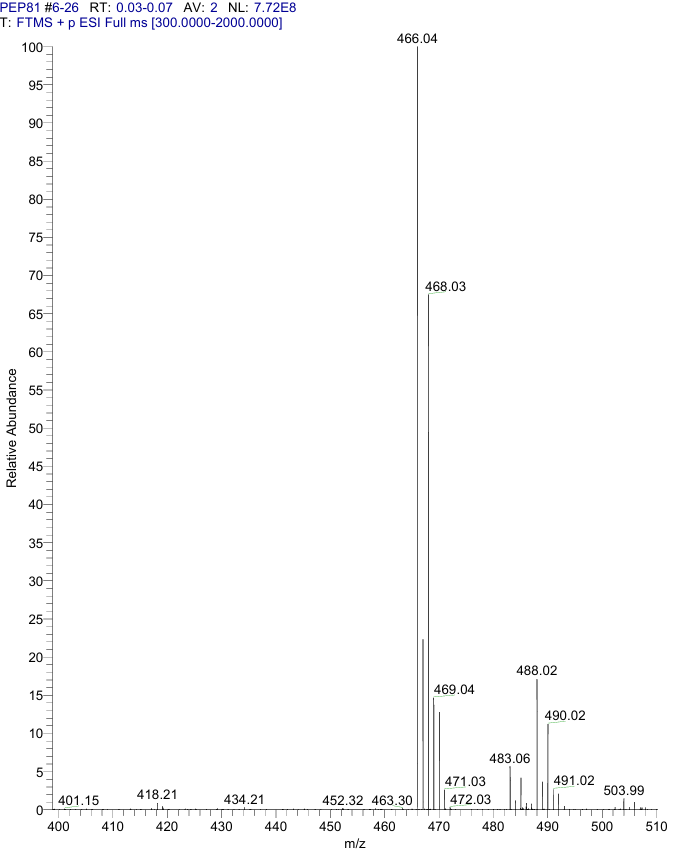
**

**Fig. S18**. HRMS (ESI-Orbitrap) spectrum of compound **3d**


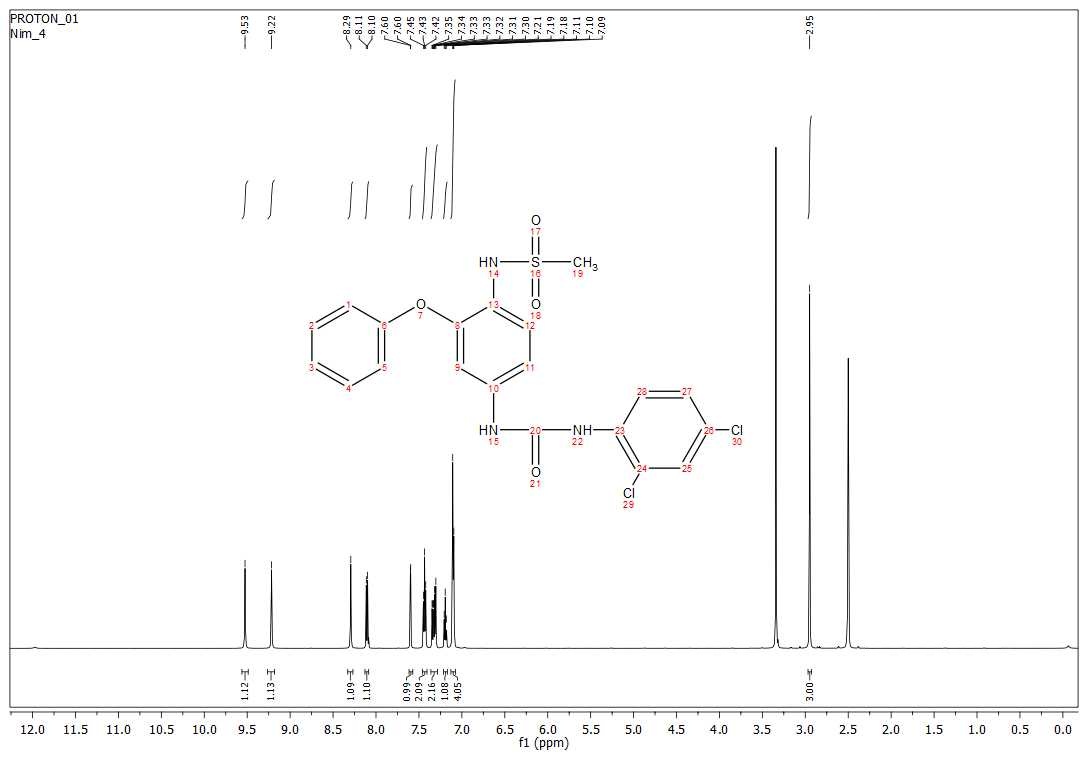


**Fig. S19**. ^1^H NMR spectrum (600 MHz, DMSO) of **3d**


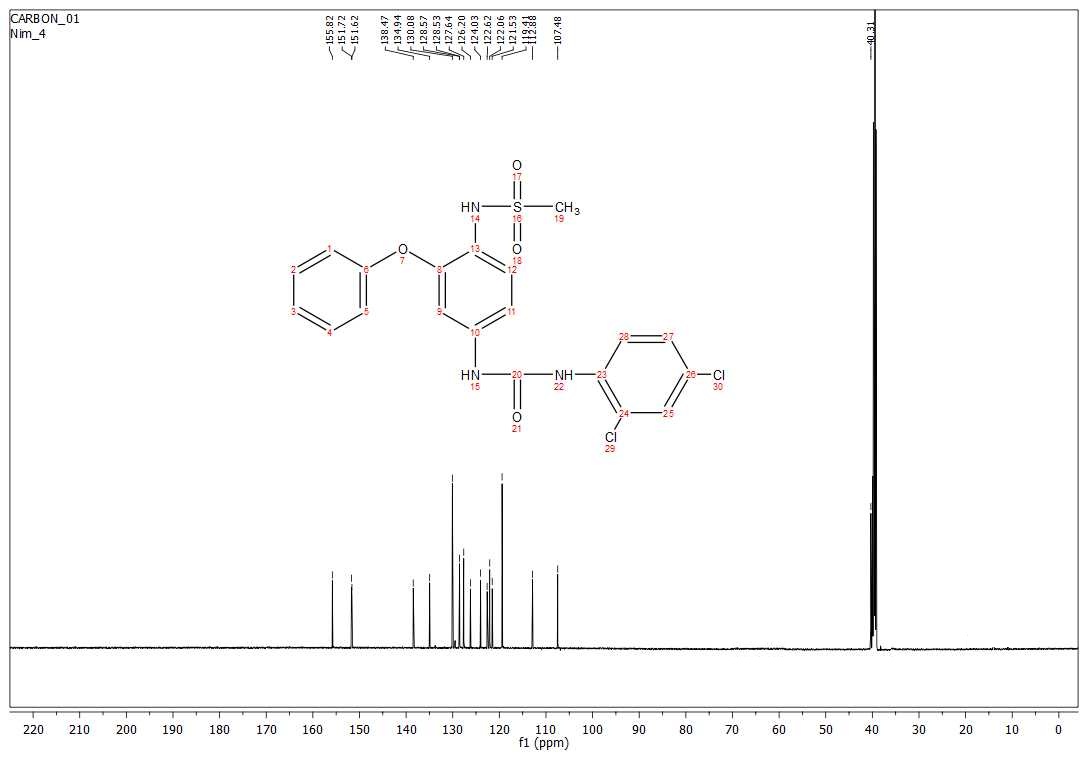


**Fig. S20**. ^13^C NMR spectrum (150 MHz, DMSO) of **3d.**


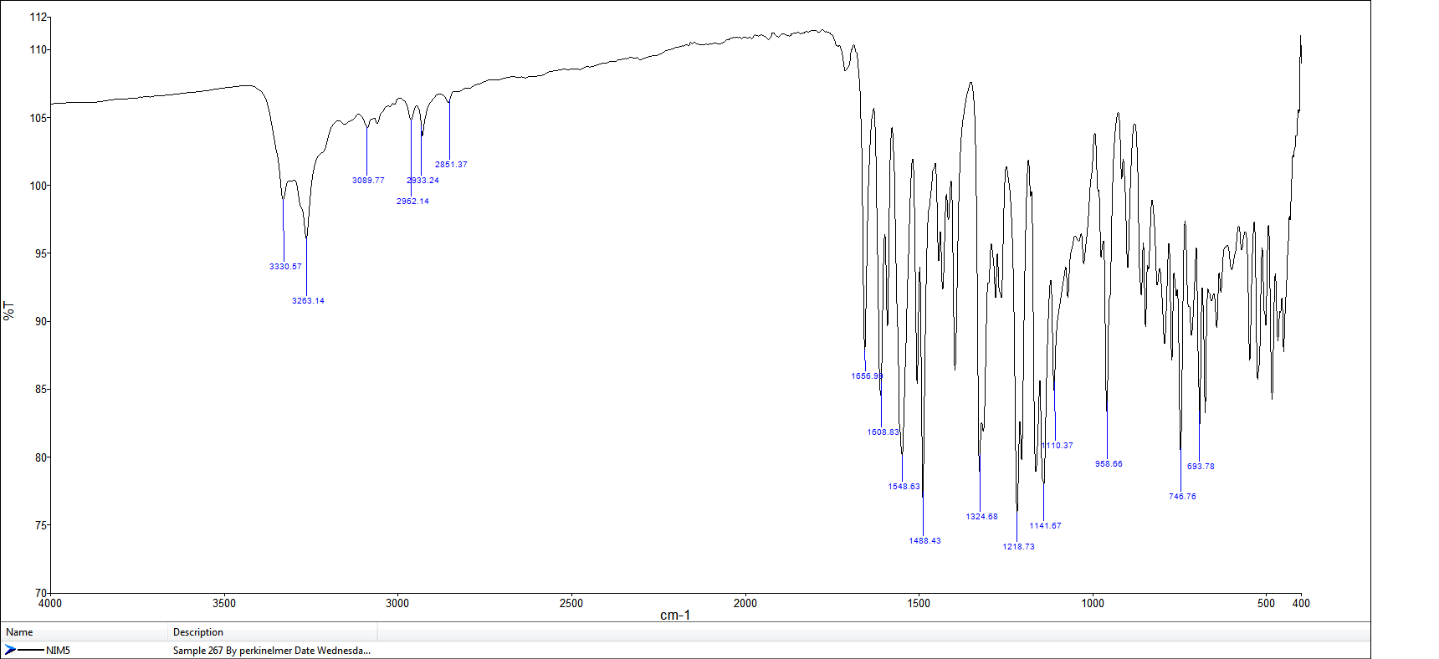
**Copies of the FTIR, HRMS, ^1^H NMR and ^13^C NMR spectra of synthesized N-[4-[3-(3-Fluoro-phenyl)-ureido]-2-phenoxy-phenyl]-methanesulfonamide (3e)**

**Fig. S21**. FTIR spectrum of **3e**

**
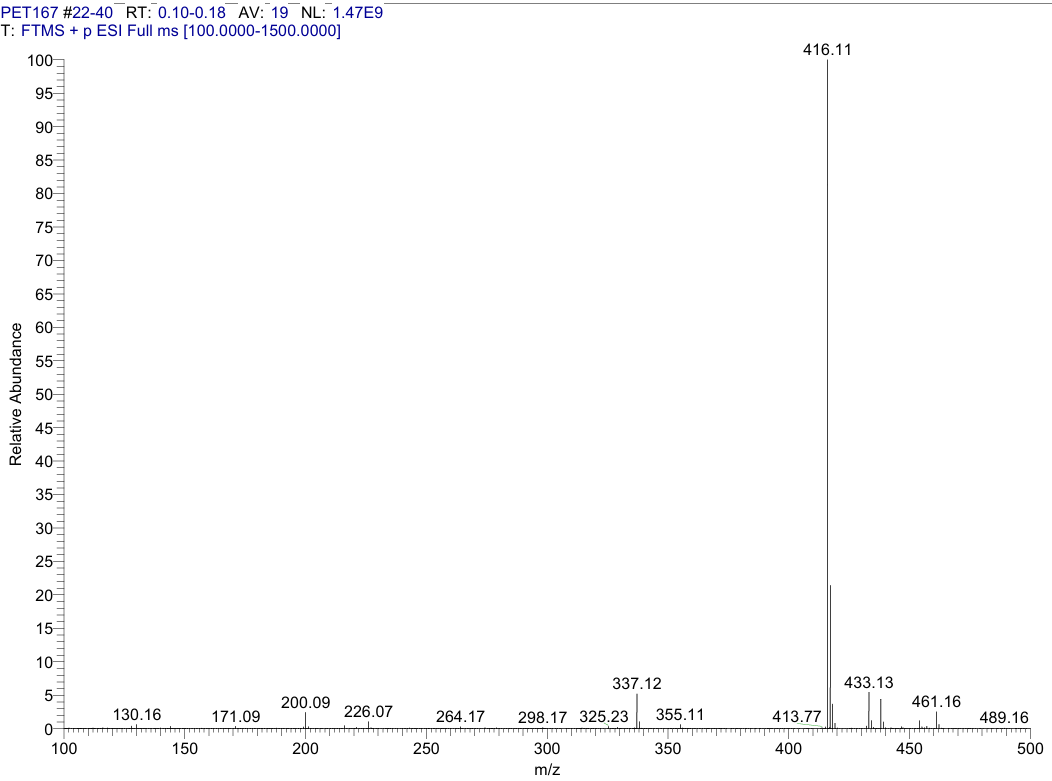
**

**Fig. S22**. HRMS (ESI-Orbitrap) spectrum of compound **3e**


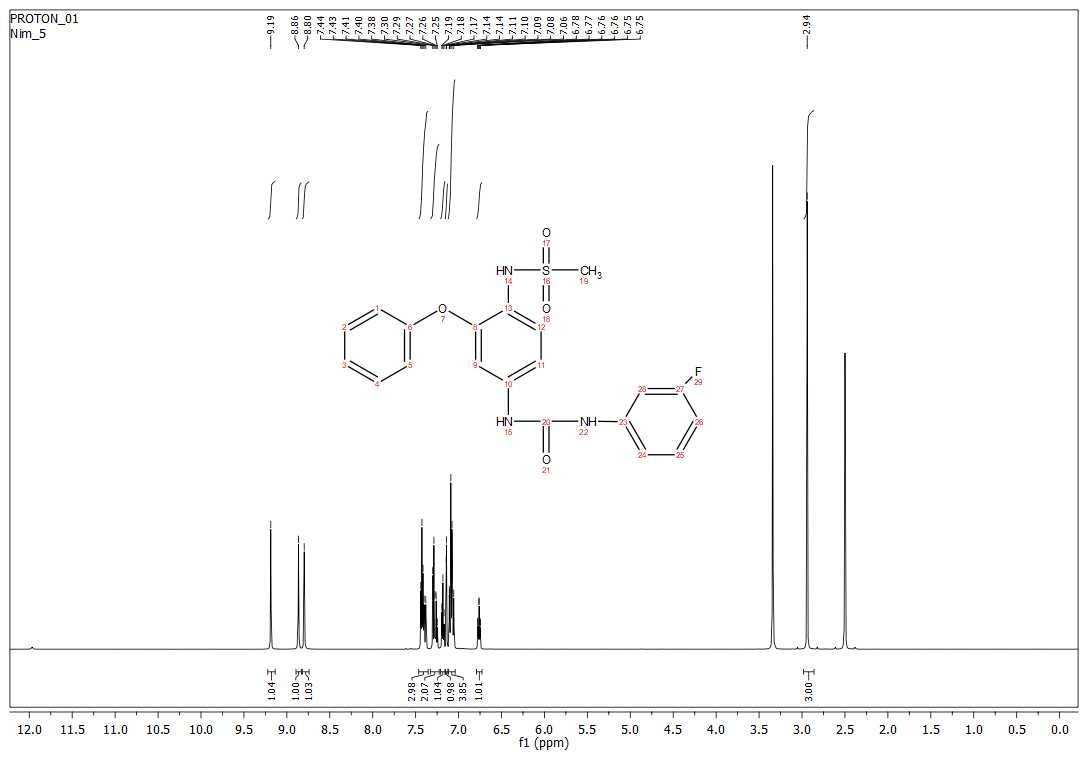


**Fig. S23**. ^1^H NMR spectrum (600 MHz, DMSO) of **3e**


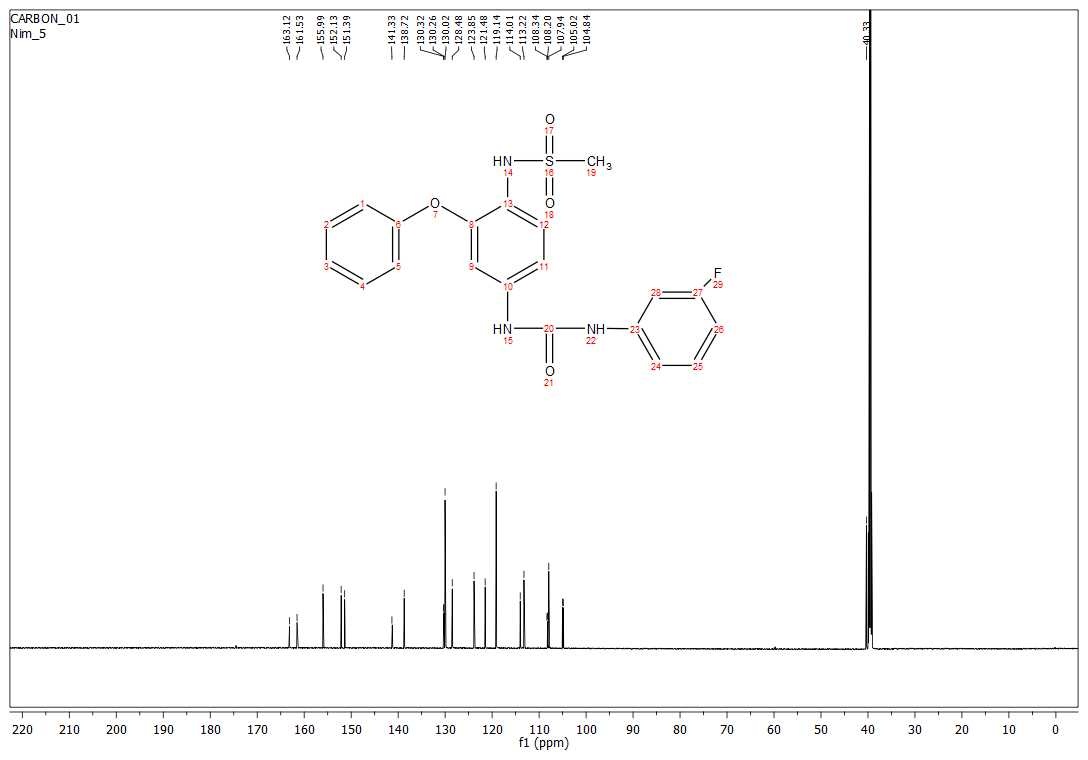


**Fig. S24**. ^13^C NMR spectrum (150 MHz, DMSO) of **3e.**

**Copies of the FTIR, HRMS, ^1^H NMR and ^13^C NMR spectra of synthesized N-{4-[3-(4-Fluoro-phenyl)-ureido]-2-phenoxy-phenyl}-methanesulfonamide (3f)**


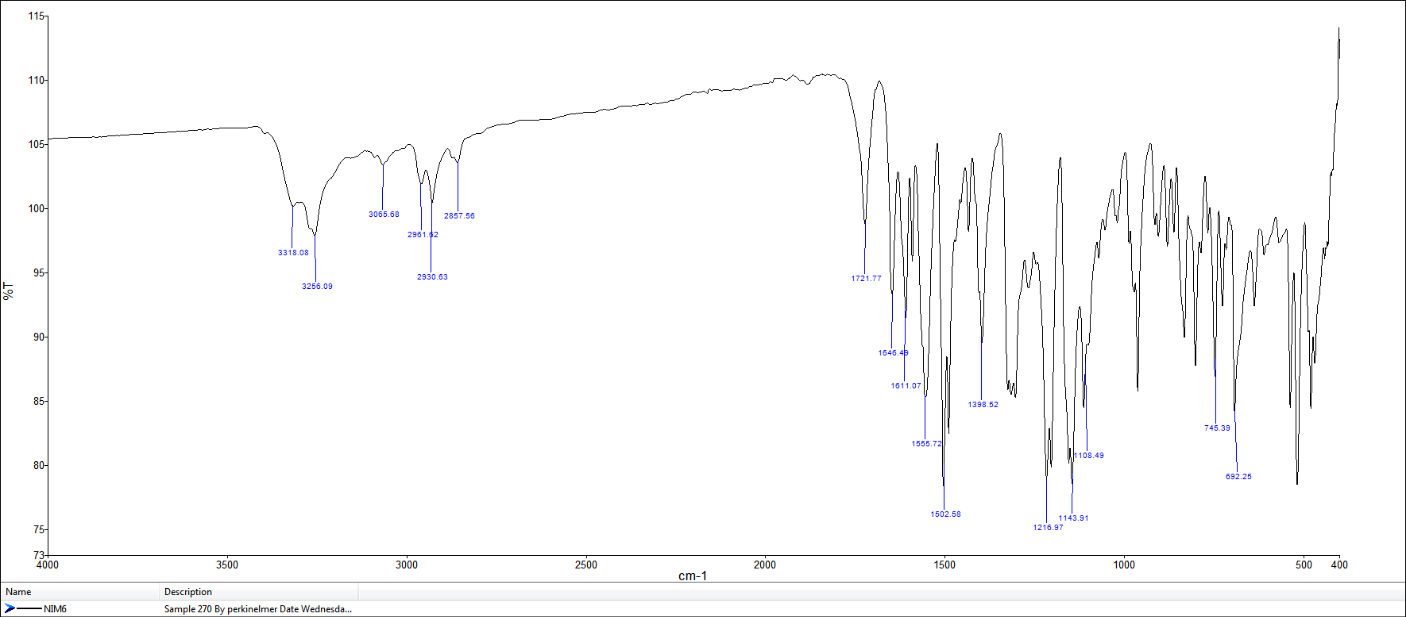


**Fig. S25**. FTIR spectrum of **3f**


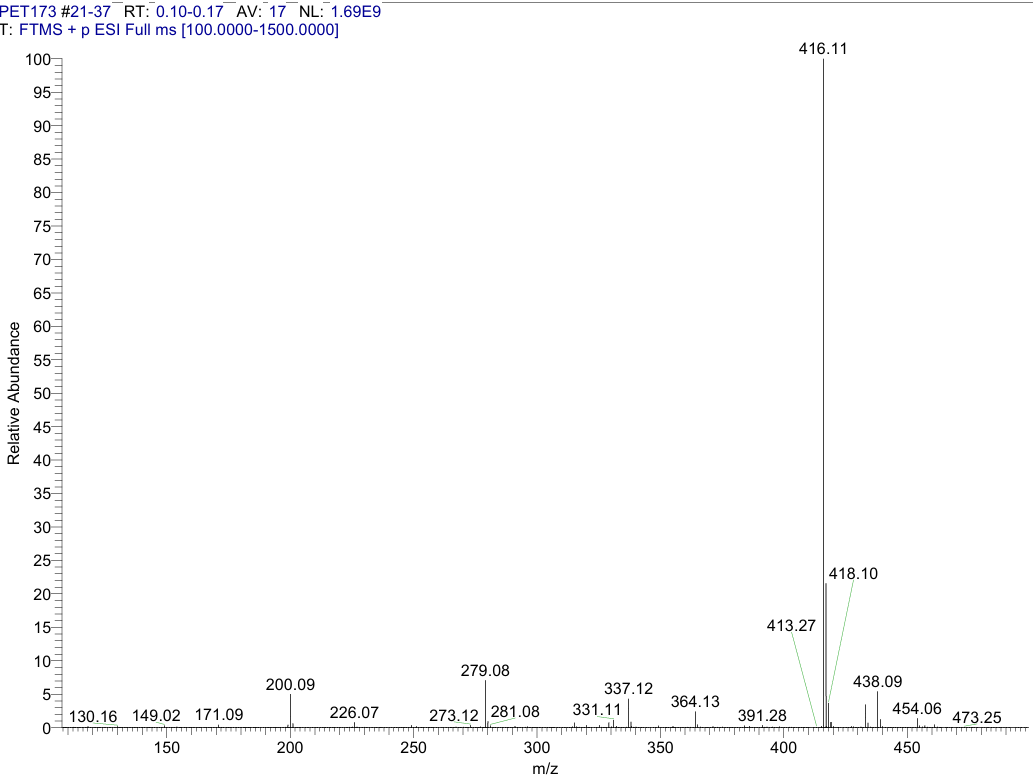


**Fig. S26**. HRMS (ESI-Orbitrap) spectrum of compound **3f.**


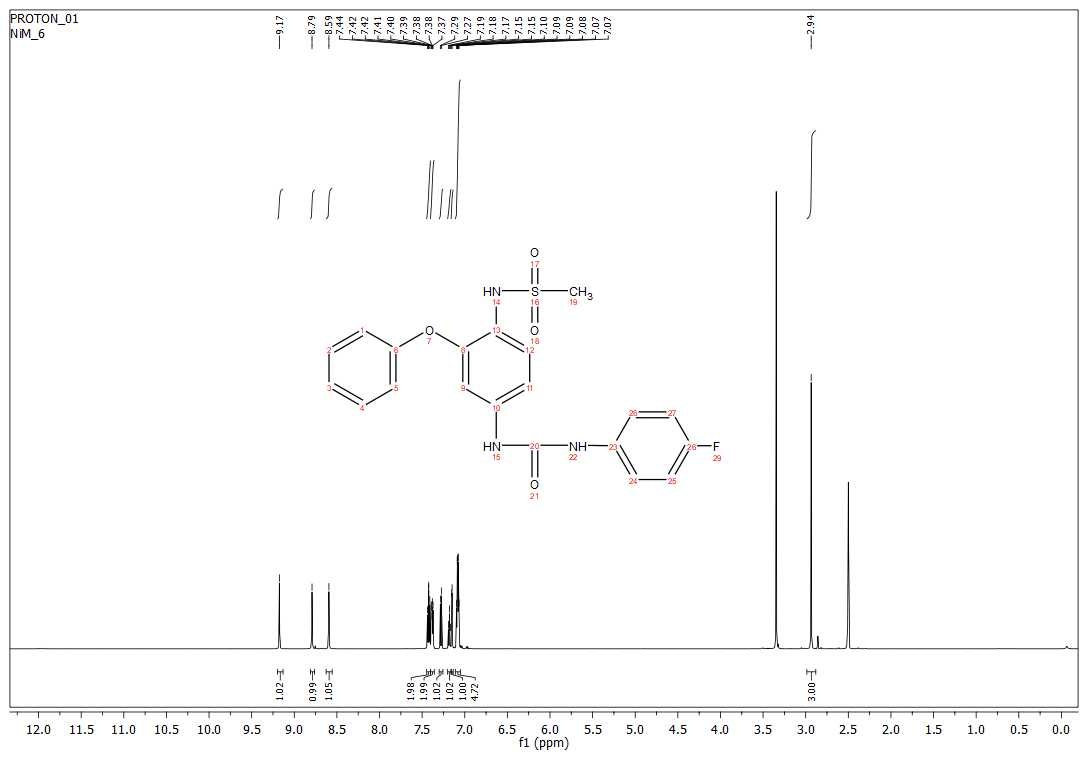


**Fig. S27**. ^1^H NMR spectrum (600 MHz, DMSO) of **3f.**


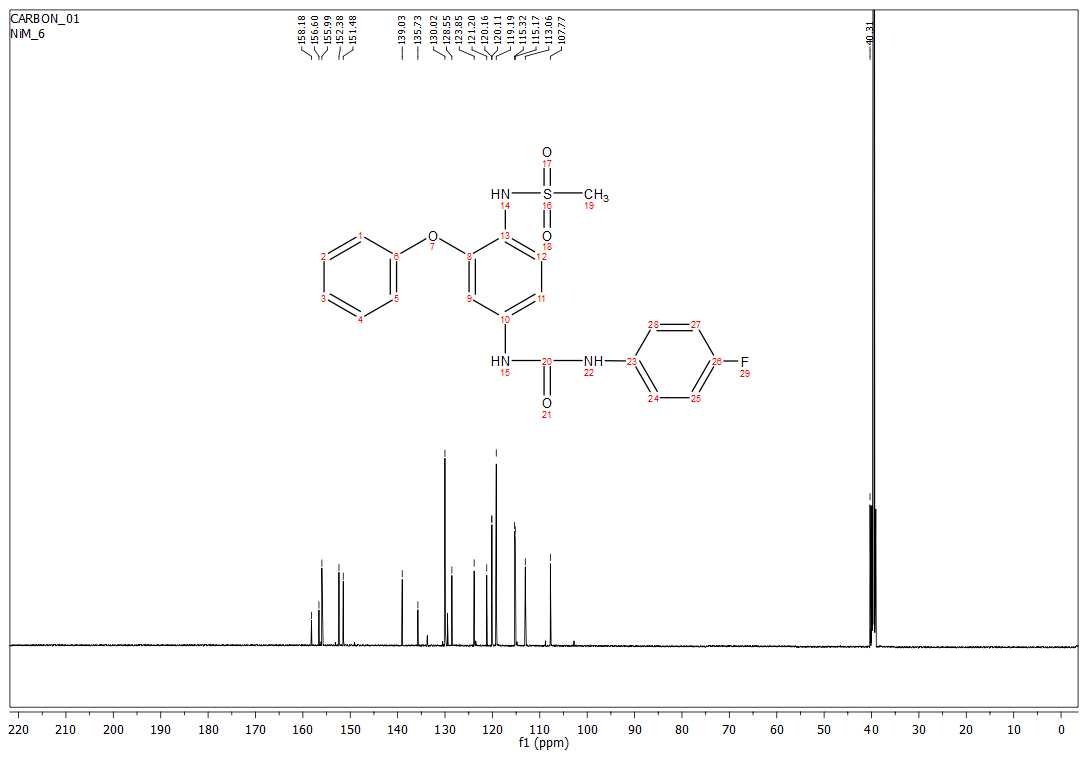


**Fig. S28**. ^13^C NMR spectrum (150 MHz, DMSO) of **3f.**


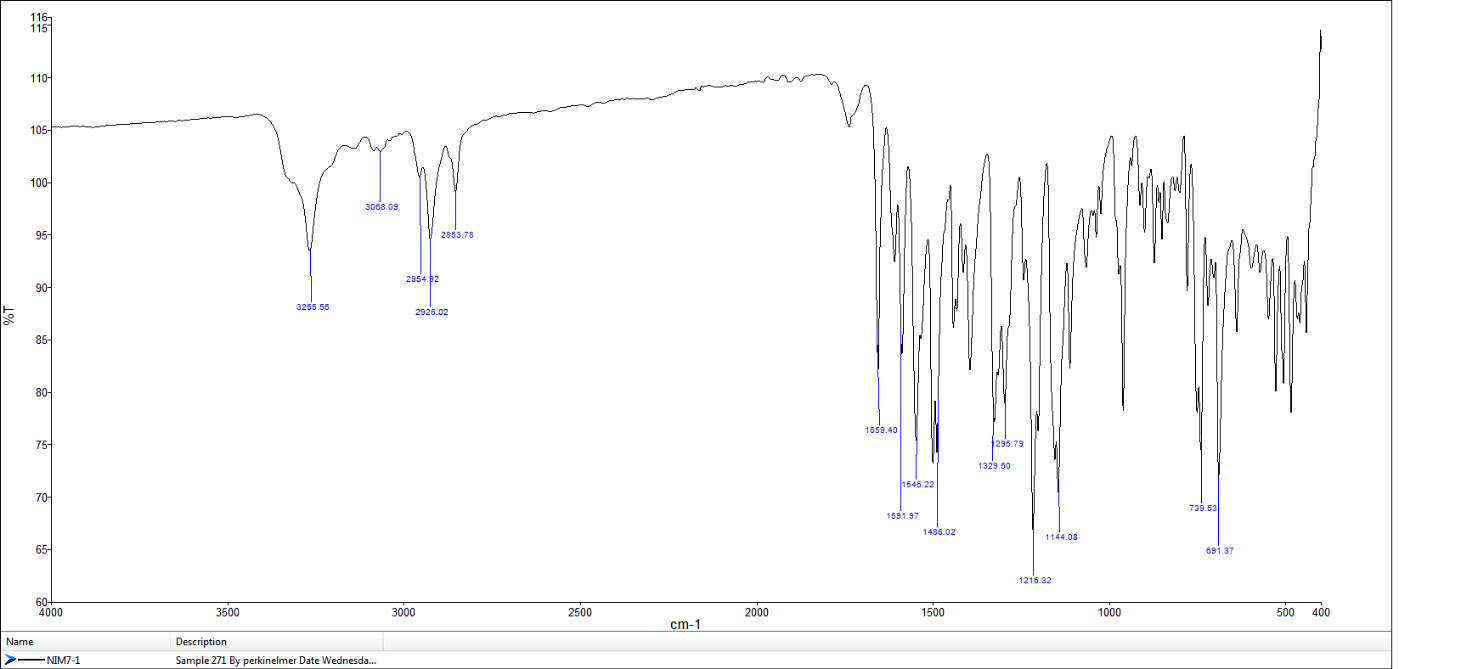
**Copies of the FTIR, HRMS, ^1^H NMR and ^13^C NMR spectra of synthesized N-{4-[3-(2-Chloro-phenyl)-ureido]-2-phenoxy-phenyl}-methanesulfonamide (3g)**

**Fig. S29**. FTIR spectrum of **3g**

**
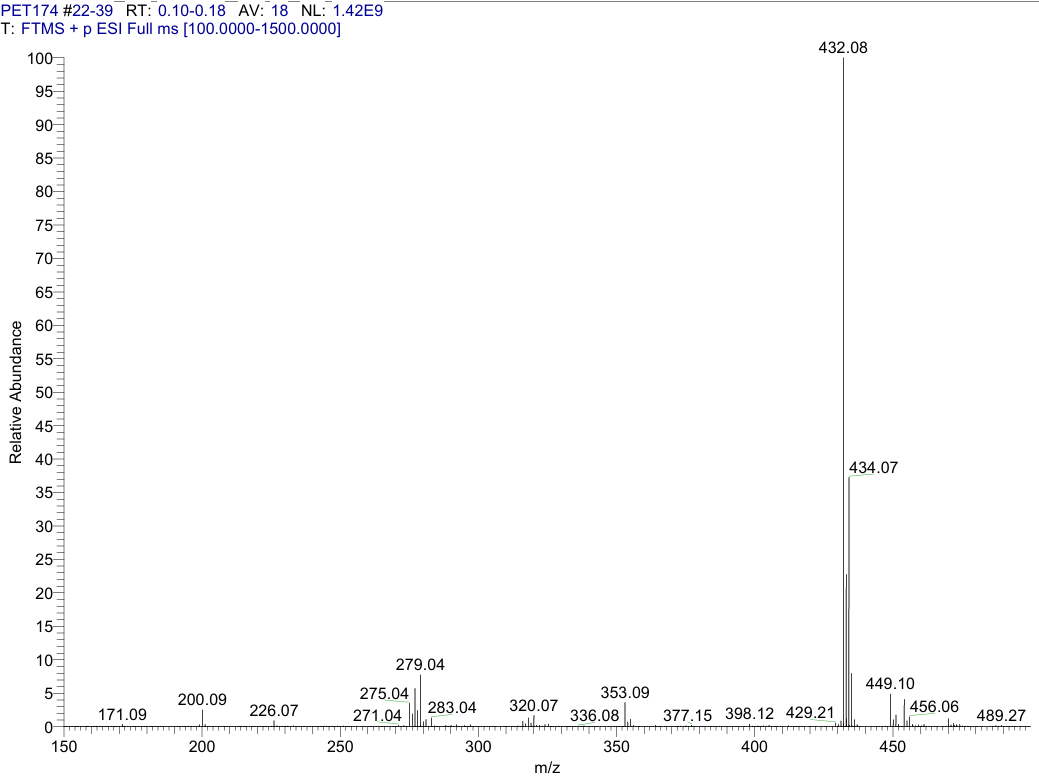
**

**Fig. S30**. HRMS (ESI-Orbitrap) spectrum of compound **3g**


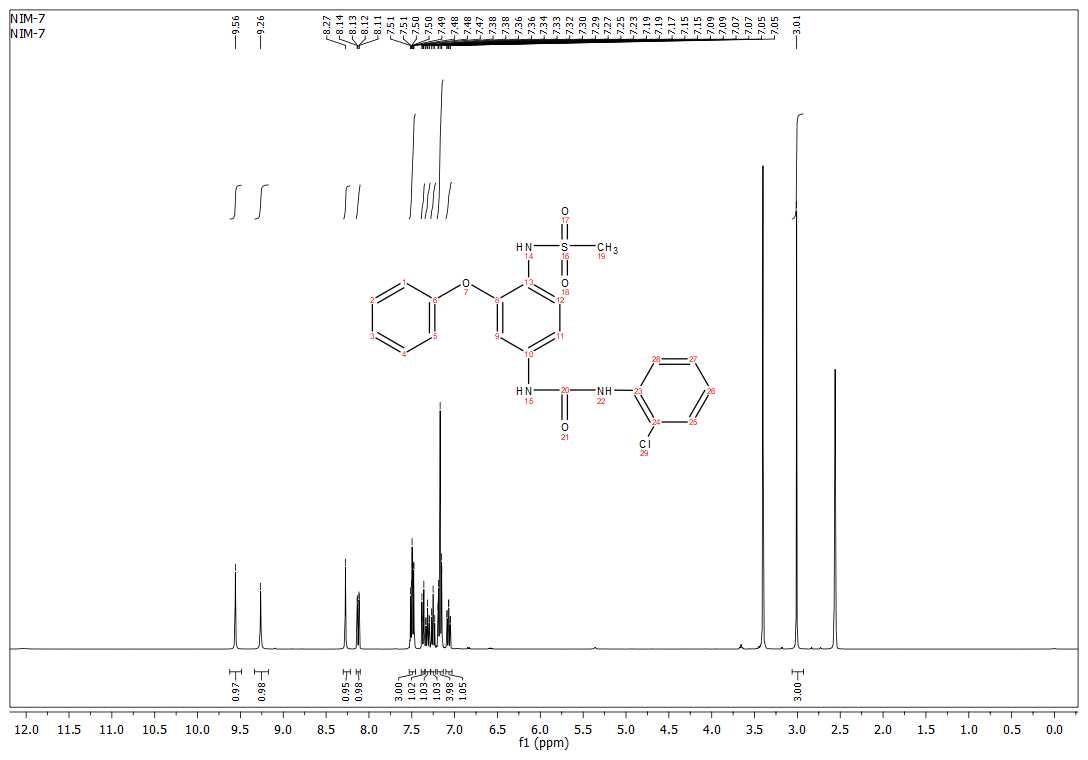


**Fig. S31**. ^1^H NMR spectrum (600 MHz, DMSO) of **3g.**


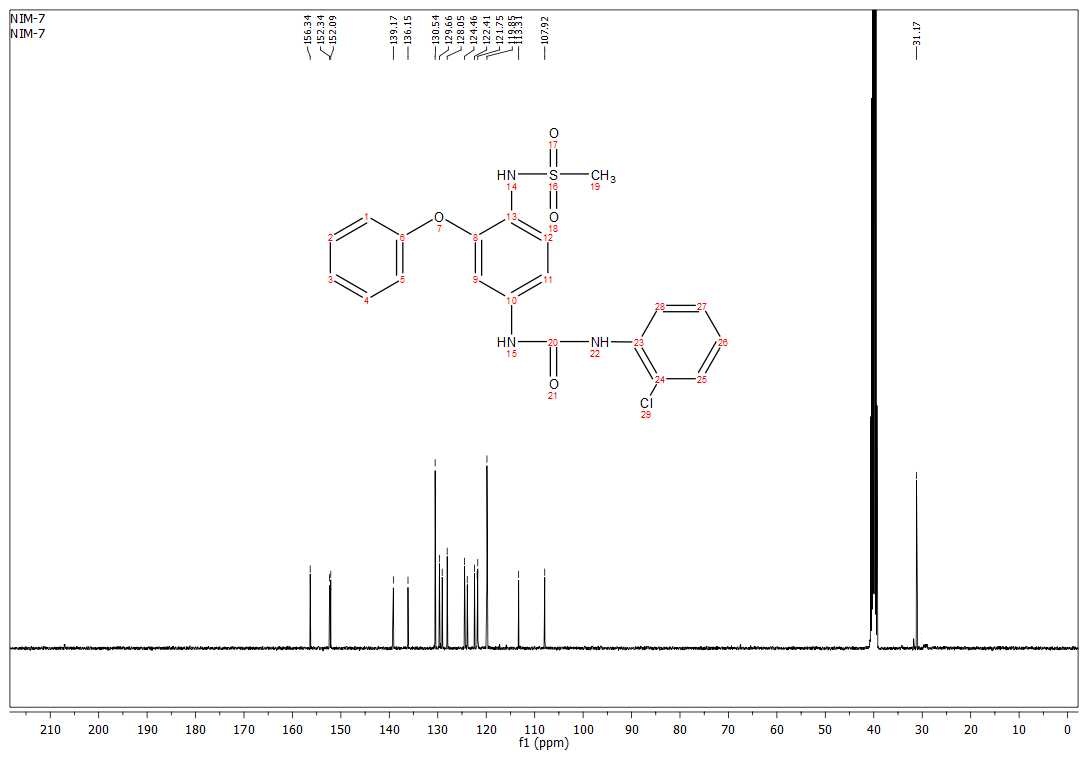


**Fig. S32**. ^13^C NMR spectrum (150 MHz, DMSO) of **3g.**


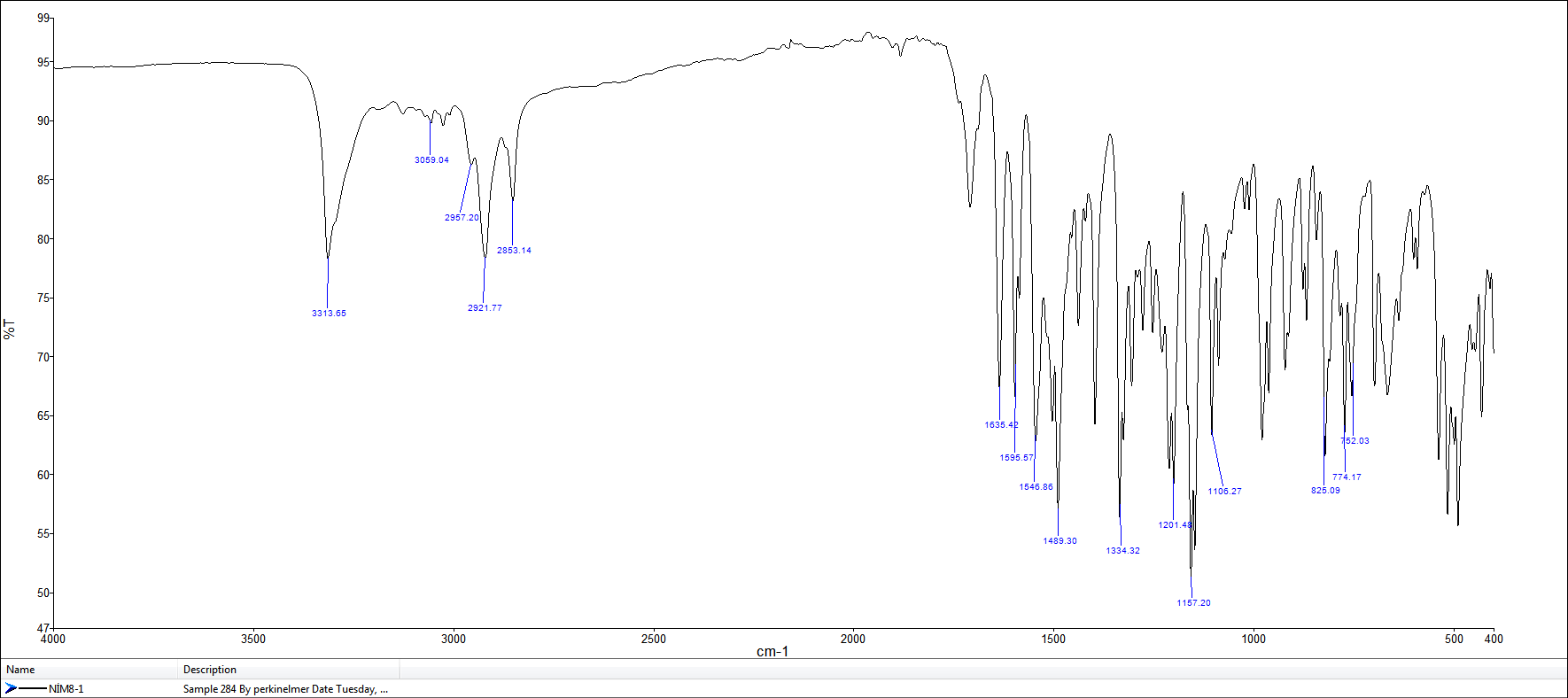
**Copies of the FTIR, HRMS, ^1^H NMR and ^13^C NMR spectra of synthesized N-{4-[3-(4-Chloro-phenyl)-ureido]-2-phenoxy-phenyl}-methanesulfonamide (3h)**

**Fig. S33**. FTIR spectrum of **3h**


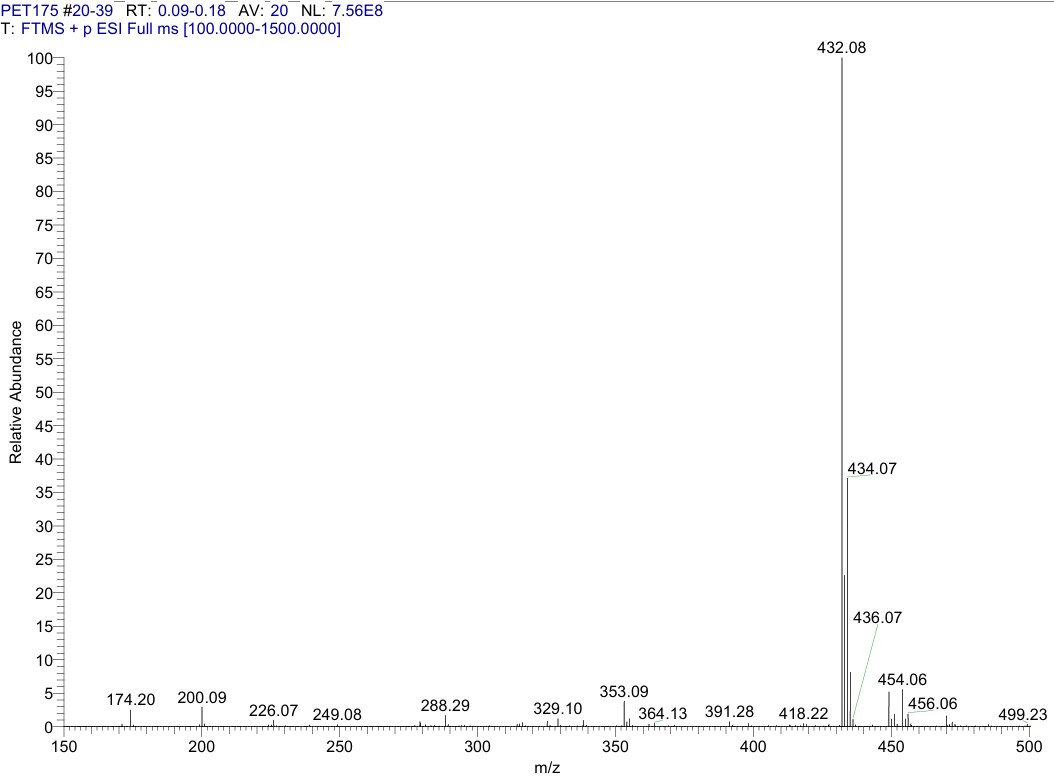


**Fig. S34**. HRMS (ESI-Orbitrap) spectrum of compound **3h**


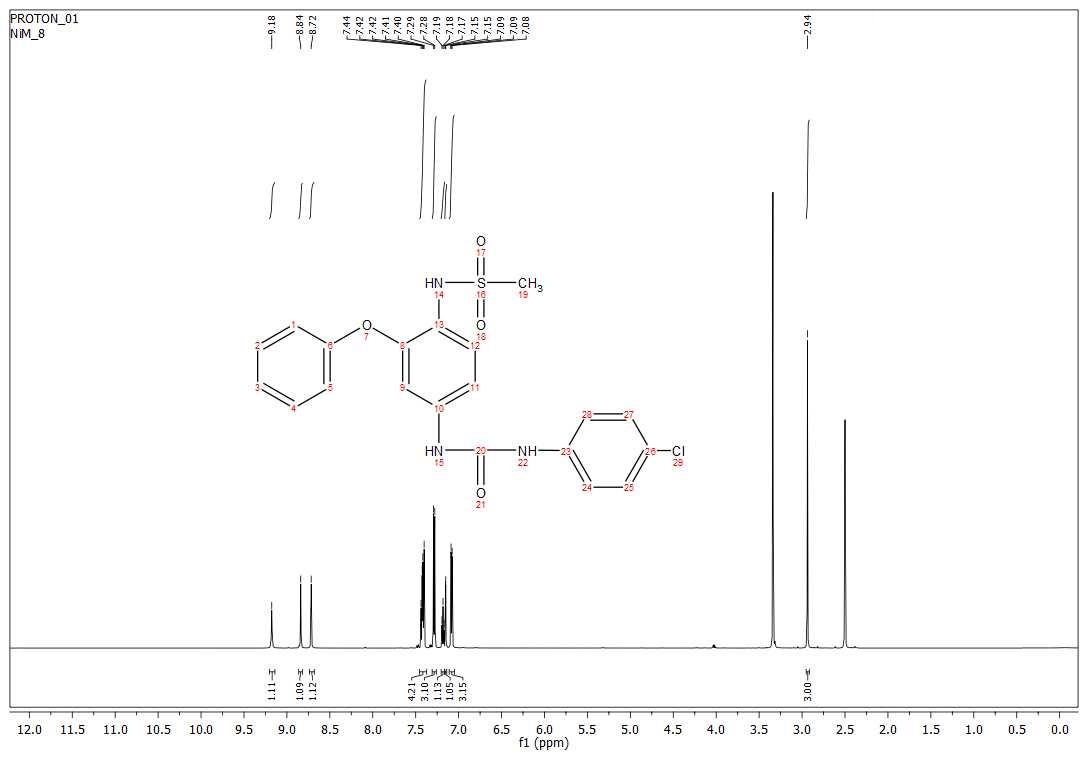


**Fig. S35**. ^1^H NMR spectrum (600 MHz, DMSO) of **3h.**


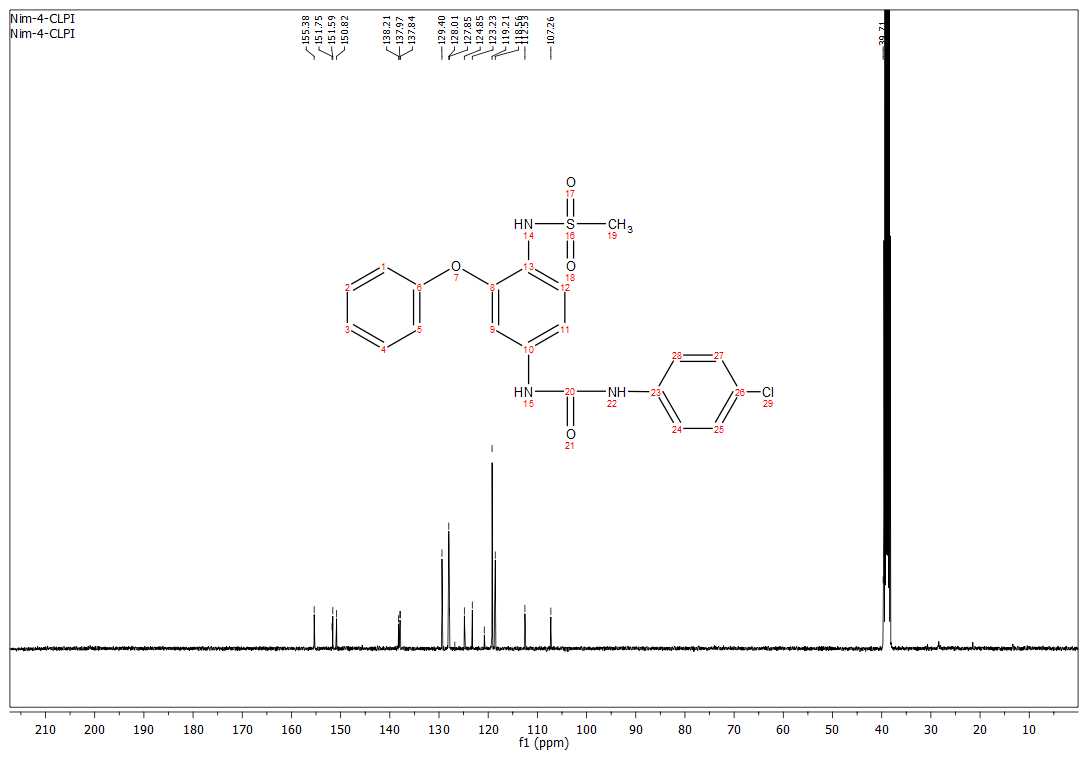


**Fig. S36**. ^13^C NMR spectrum (150 MHz, DMSO) of **3h.**

**Copies of the FTIR, HRMS, ^1^H NMR and ^13^C NMR spectra of synthesized N-[2-Phenoxy-4-(3-phenyl-ureido)-phenyl]-methanesulfonamide (3i)**


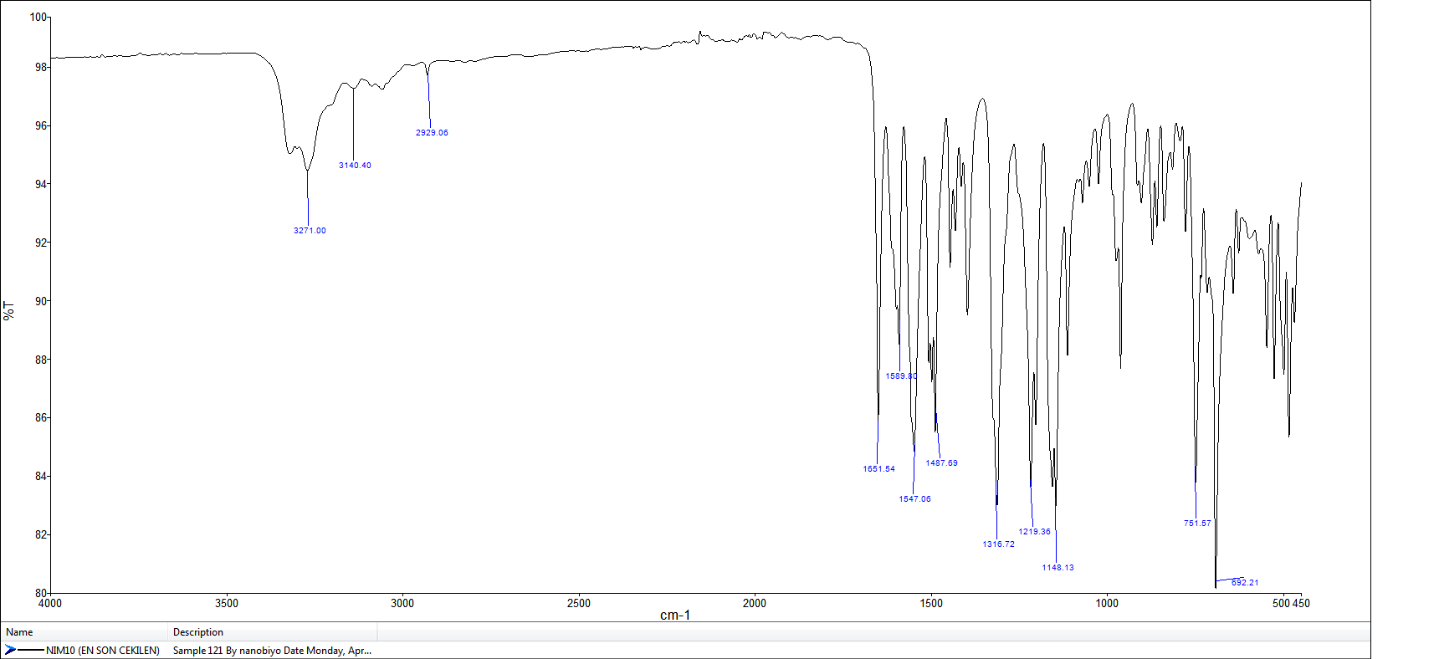


**Fig. S37**. FTIR spectrum of **3i**


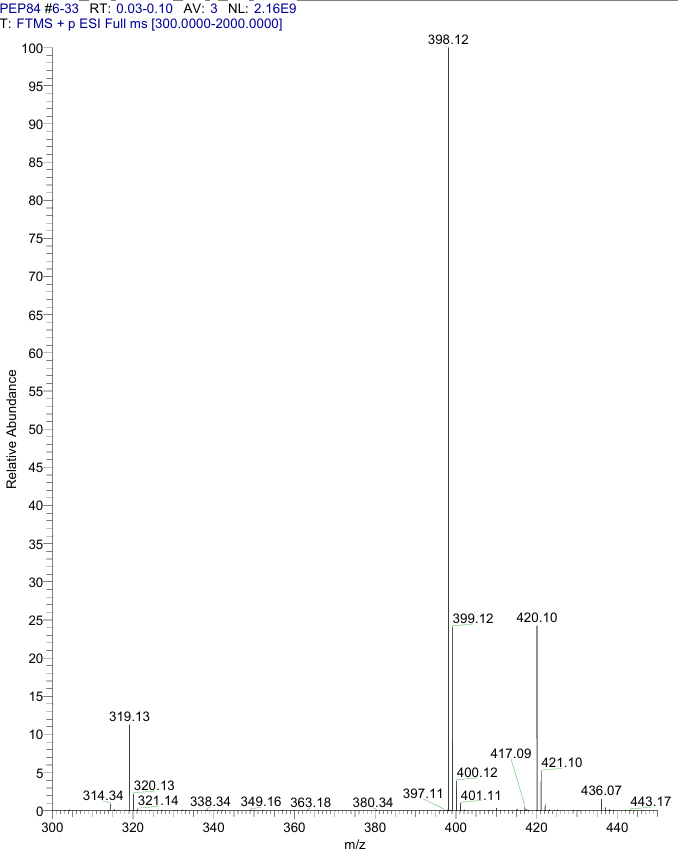


**Fig. S38**. HRMS (ESI-Orbitrap) spectrum of compound **3i**


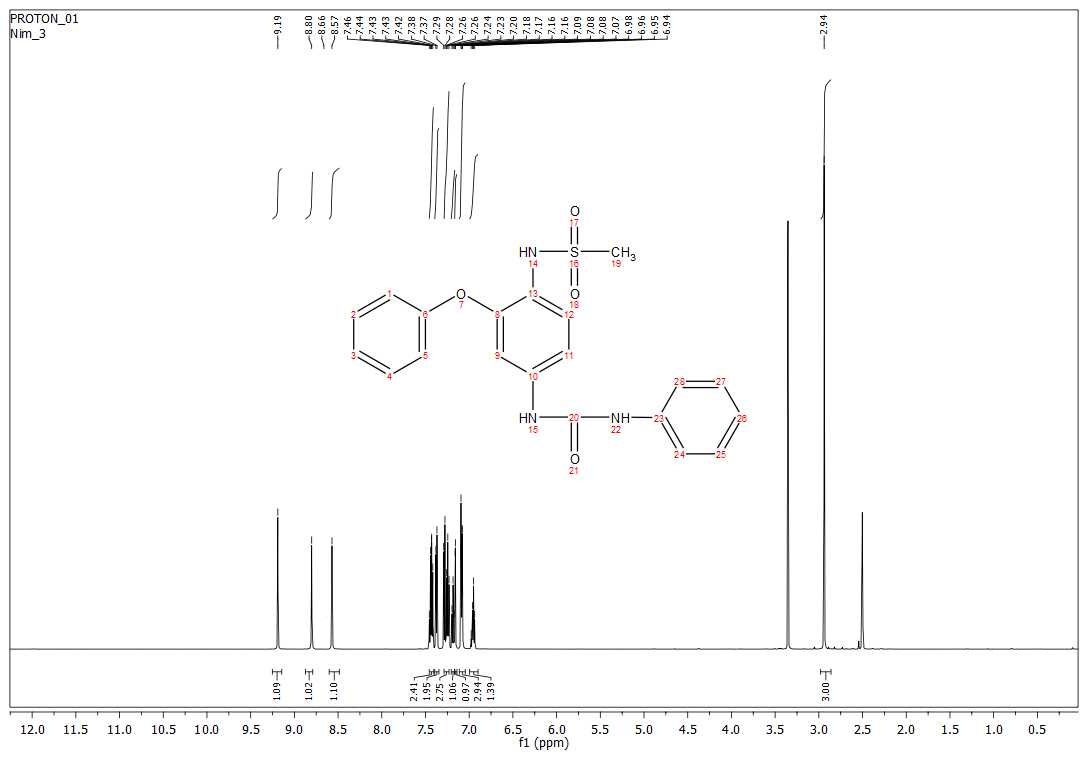


**Fig. S39**. ^1^H NMR spectrum (600 MHz, DMSO) of **3i.**


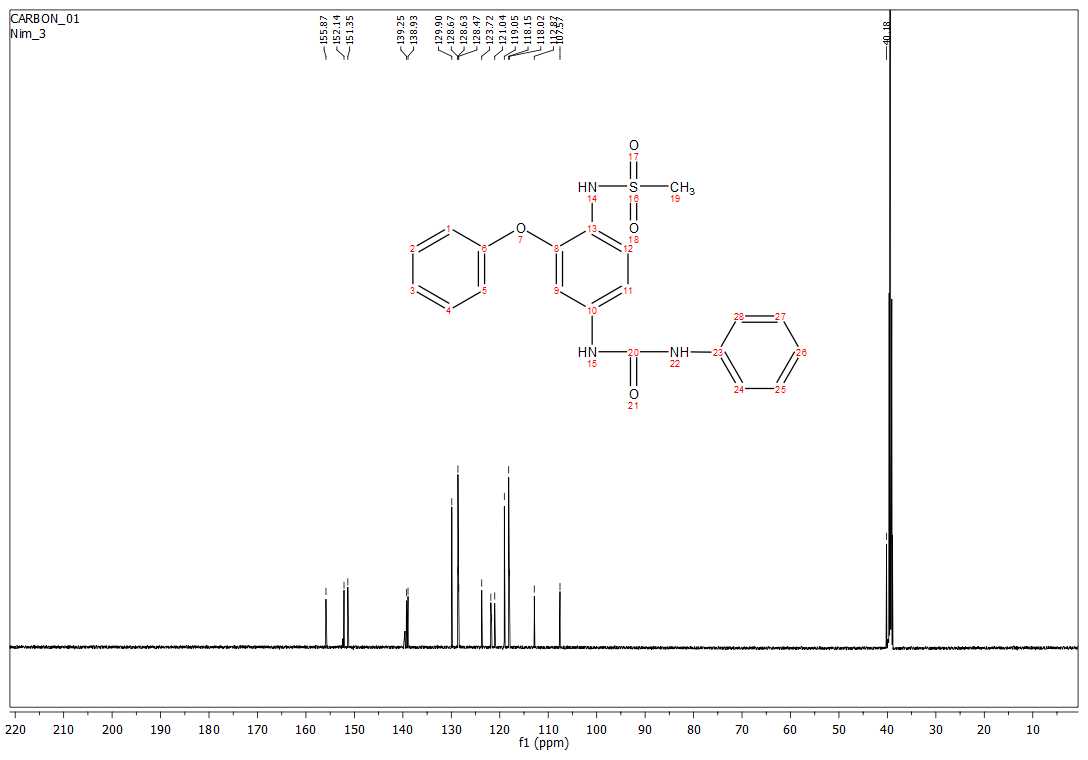


**Fig. S40**. ^13^C NMR spectrum (150 MHz, DMSO) of **3i.**

**Copies of the FTIR, HRMS, ^1^H NMR and ^13^C NMR spectra of synthesized N-[4-(3-Benzyl-ureido)-2-phenoxy-phenyl]-methanesulfonamide (3j)**


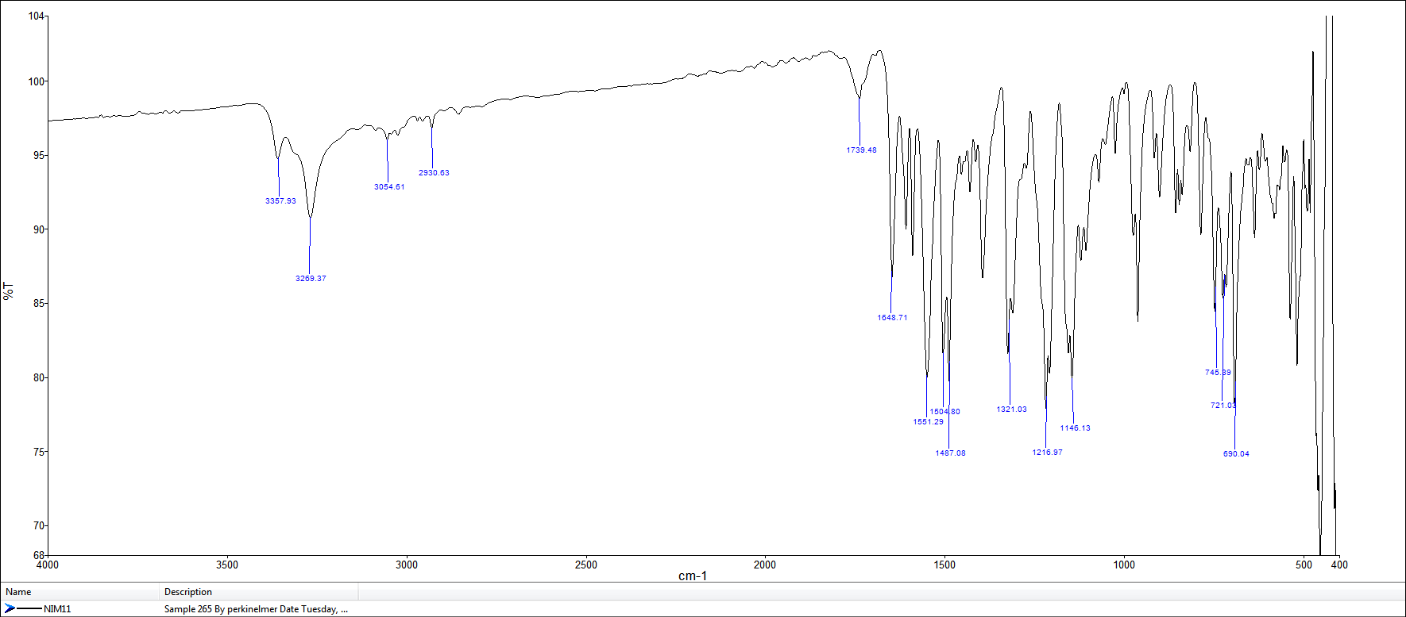


**Fig. S41**. FTIR spectrum of **3j.**

**
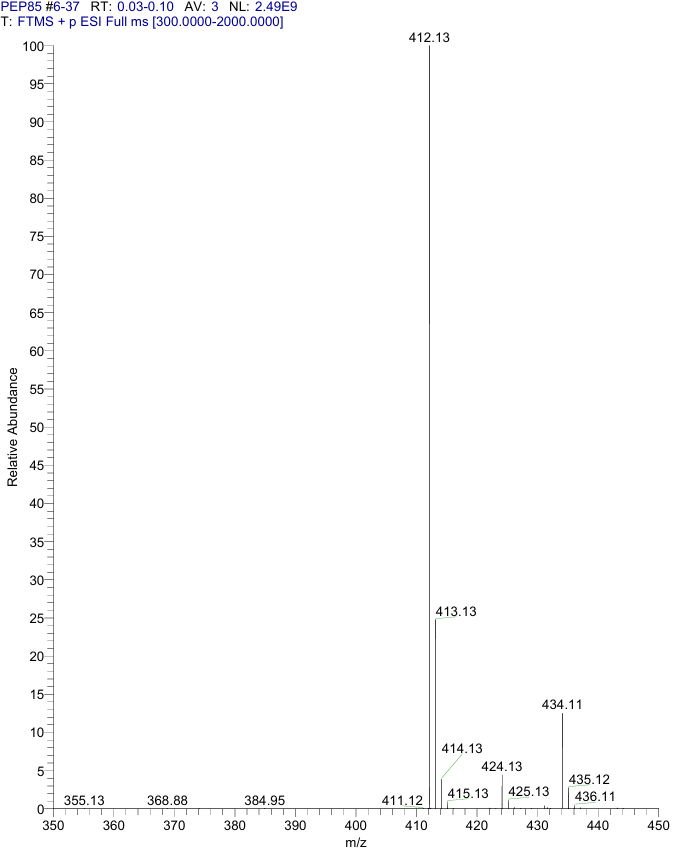
**

**Fig. S42**. HRMS (ESI-Orbitrap) spectrum of compound **3j.**


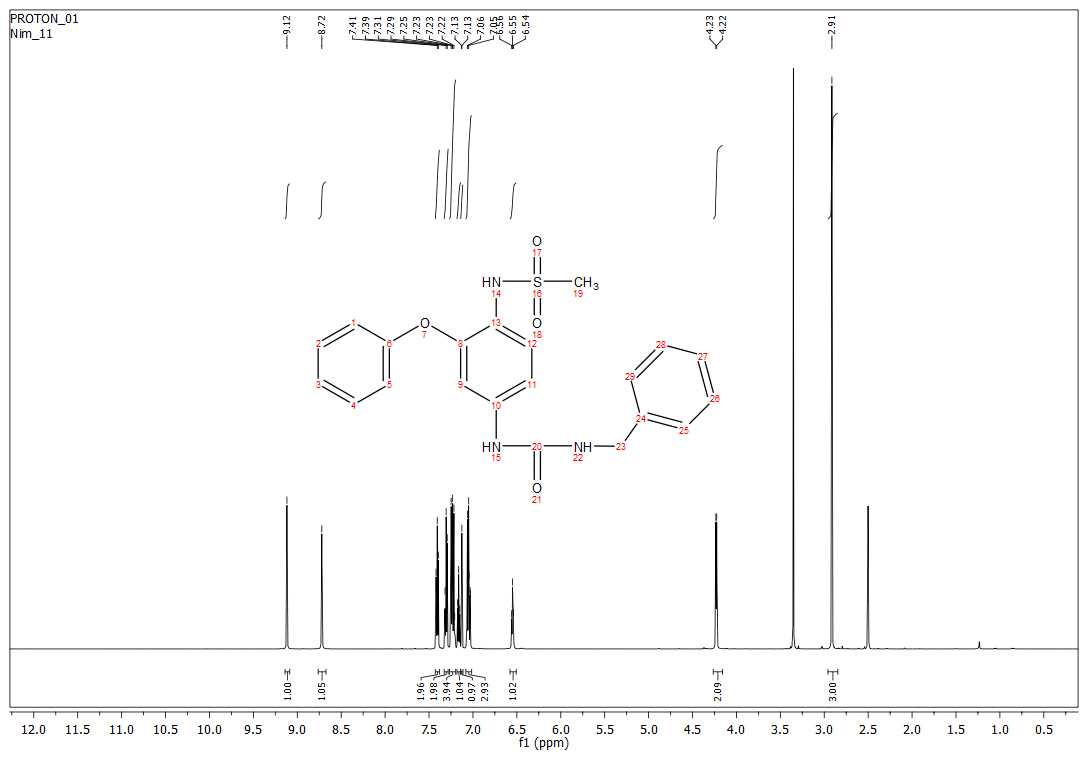


**Fig. S43**. ^1^H NMR spectrum (600 MHz, DMSO) of **3j.**


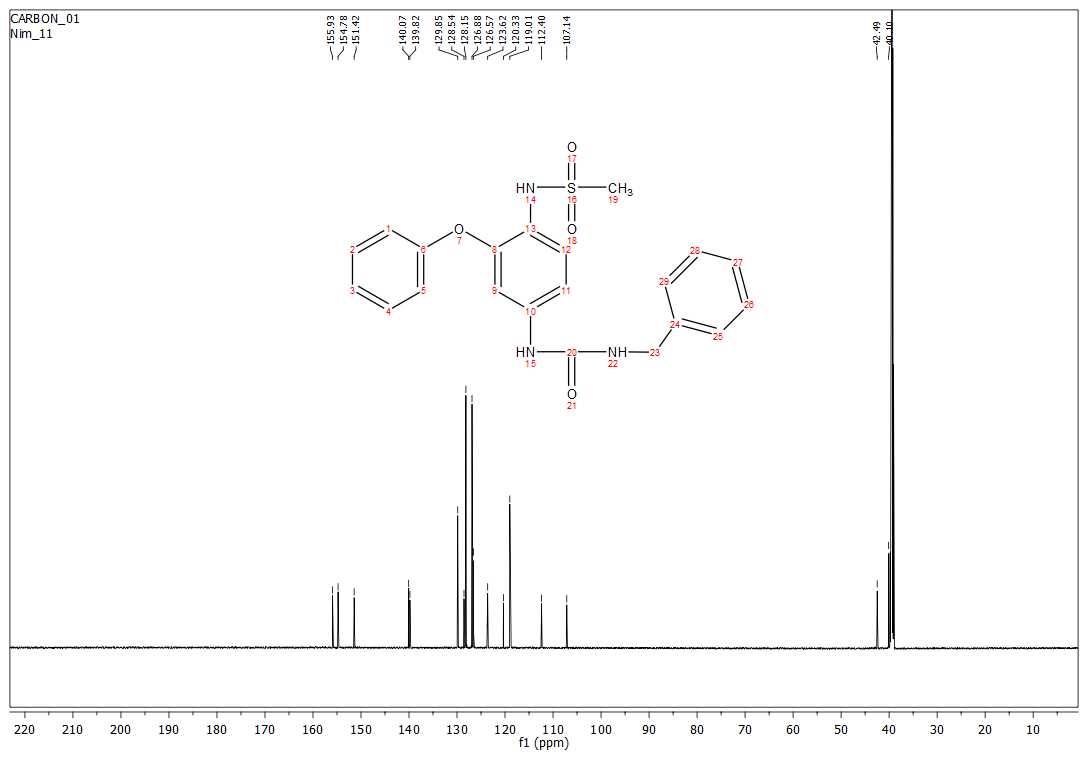


**Fig. S44**. ^13^C NMR spectrum (150 MHz, DMSO) of **3j.**

**Copies of the FTIR, HRMS, ^1^H NMR and ^13^C NMR spectra of synthesized N-{4-[3-(4-Methoxy-phenyl)-ureido]-2-phenoxy-phenyl}-methanesulfonamide (3k)**


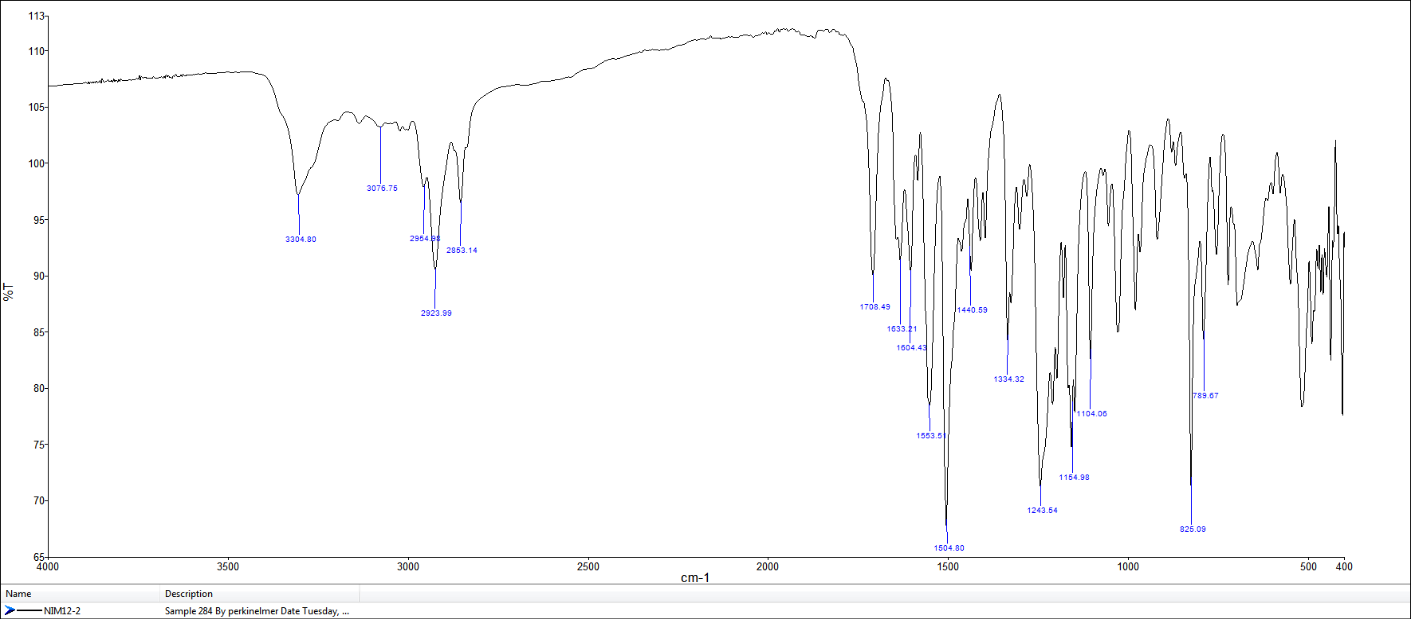


**Fig. S45**. FTIR spectrum of **3k.**

**
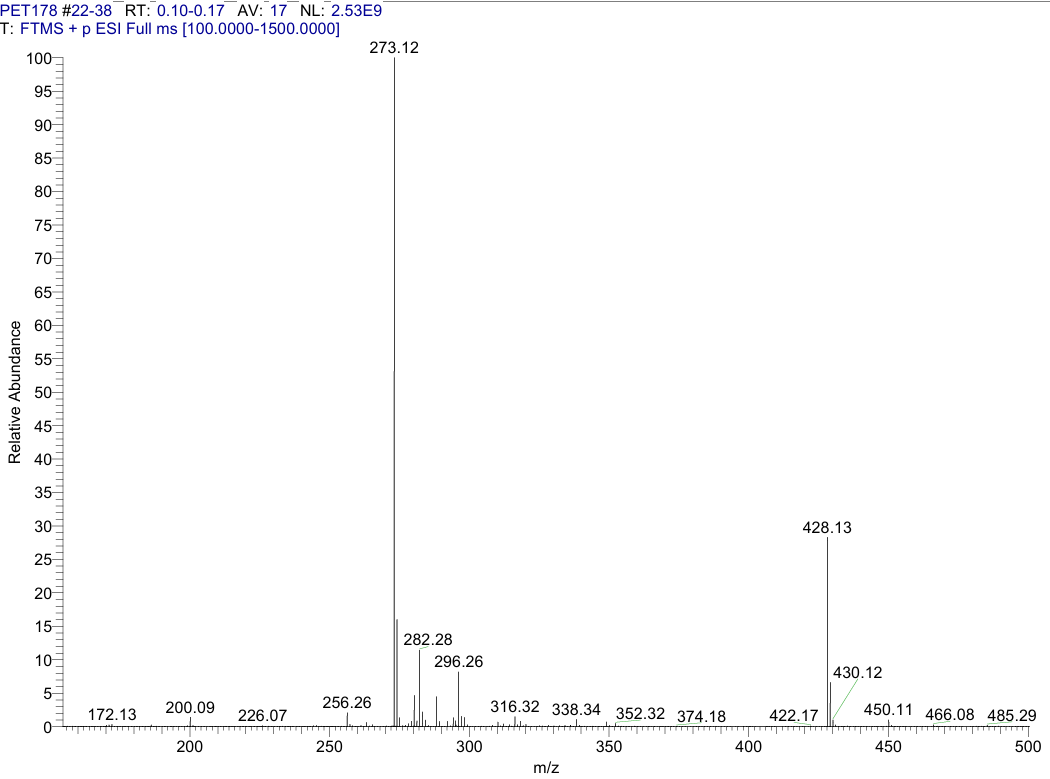
**

**Fig. S46**. HRMS (ESI-Orbitrap) spectrum of compound **3k.**


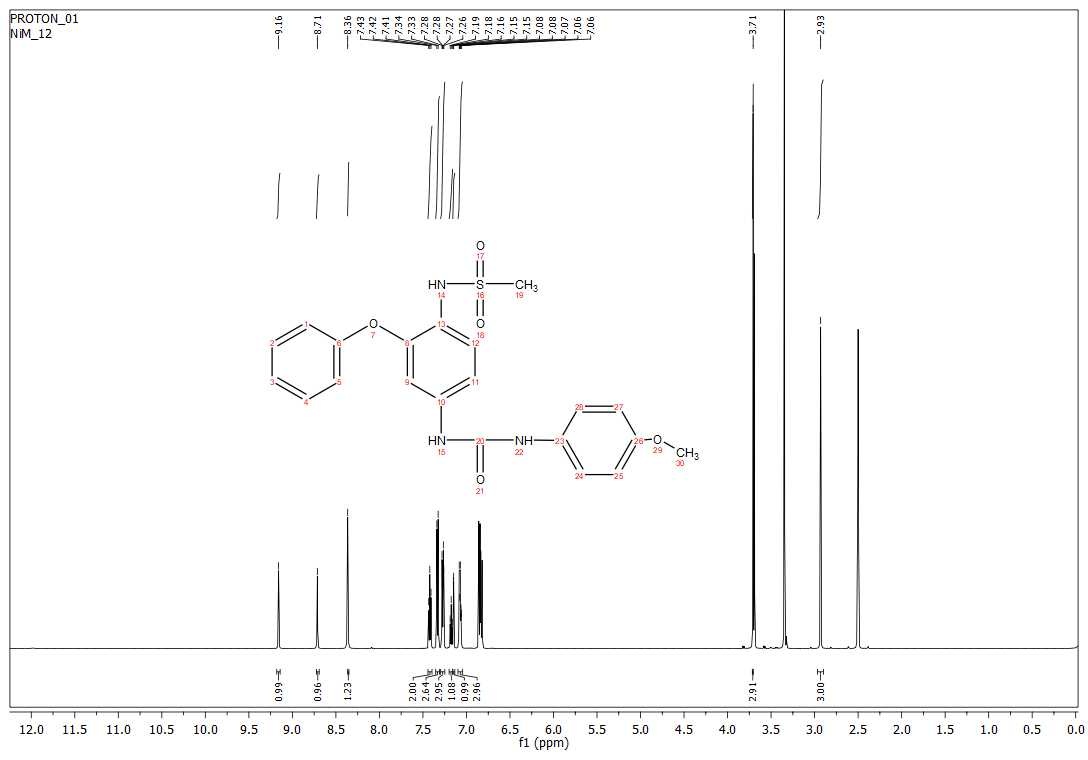


**Fig. S47**. ^1^H NMR spectrum (600 MHz, DMSO) of **3k**


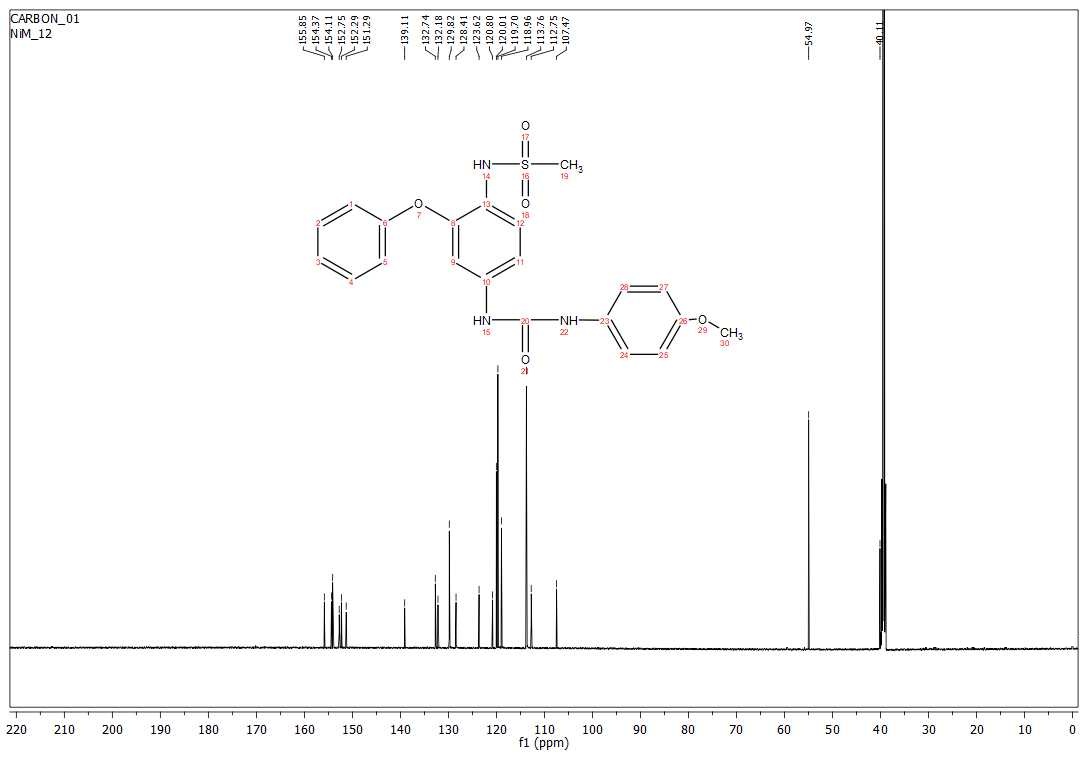


**Fig. S48**. ^13^C NMR spectrum (150 MHz, DMSO) of **3k.**

**Copies of the FTIR, HRMS, ^1^H NMR and ^13^C NMR spectra of synthesized N-[4-(3-Phenethyl-ureido)-2-phenoxy-phenyl]-methanesulfonamide (3l)**


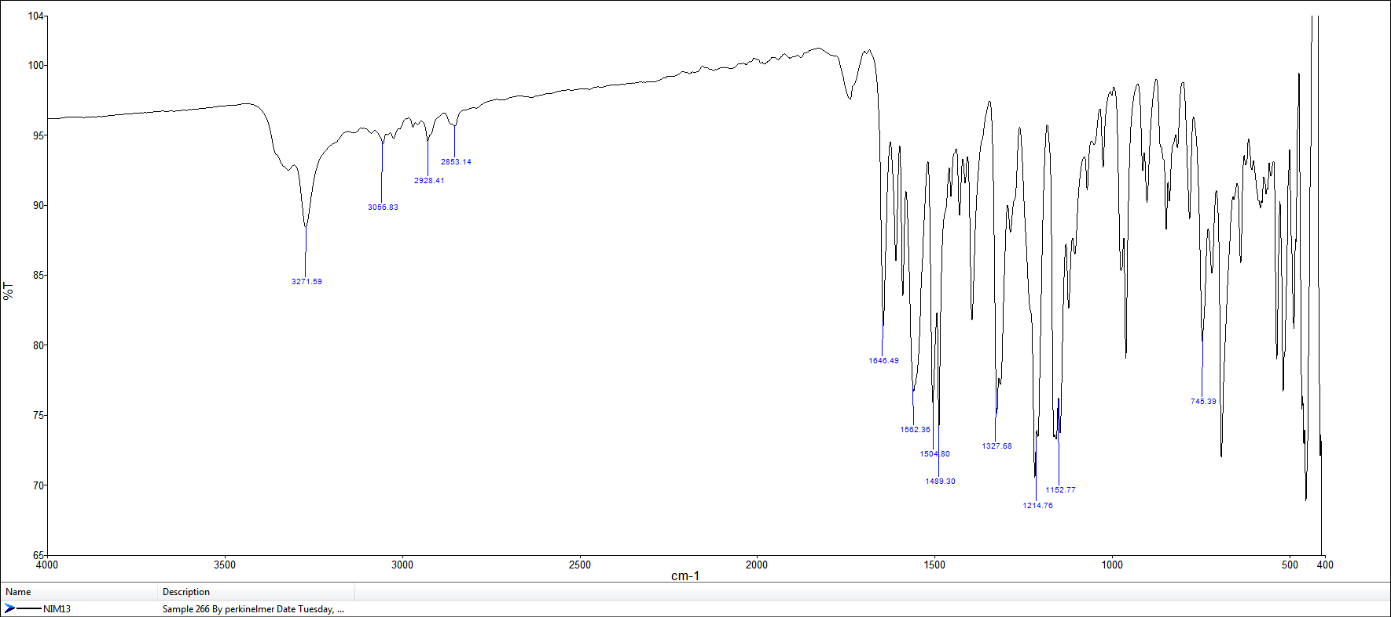


**Fig. S49**. FTIR spectrum of **3l.**


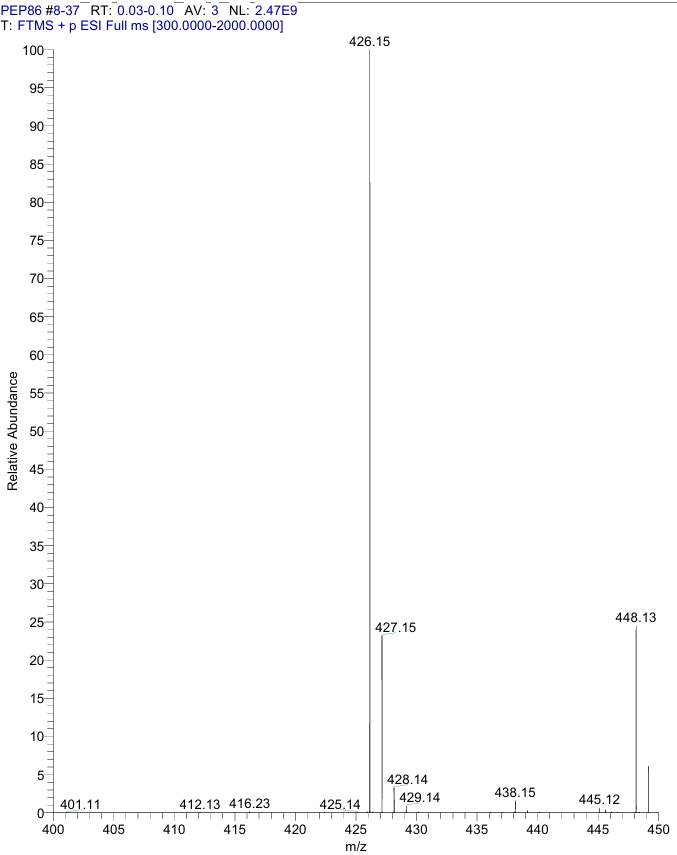


**Fig. S50**. HRMS (ESI-Orbitrap) spectrum of compound **3l.**


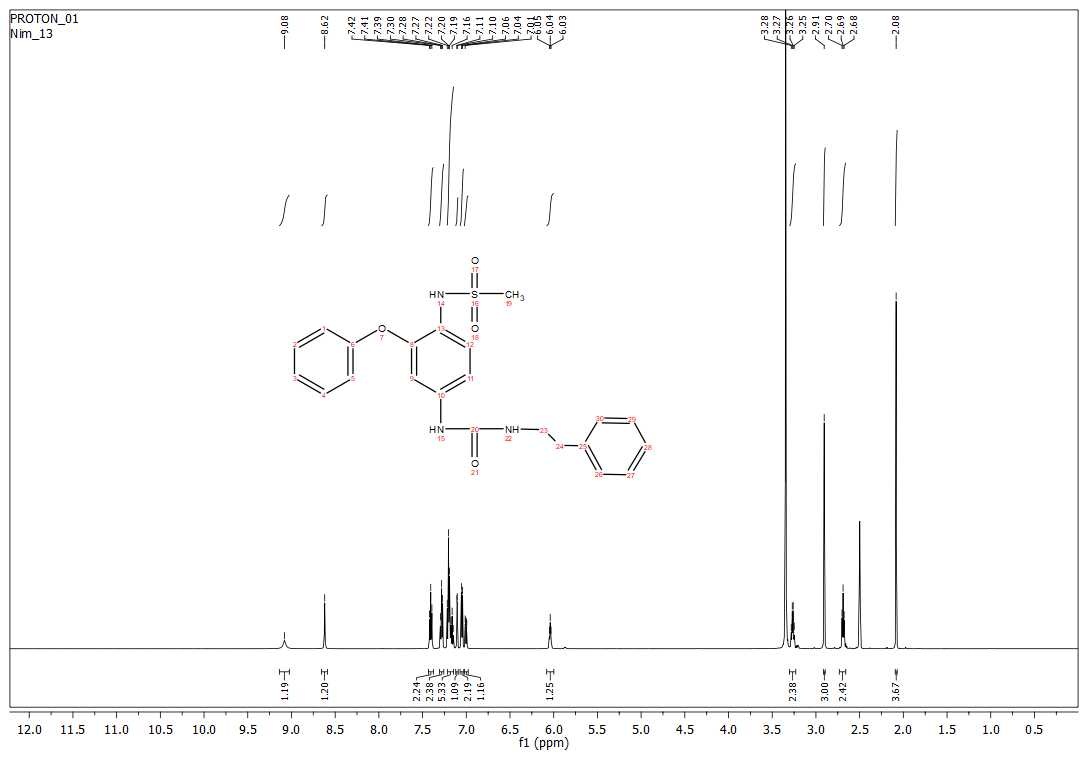


**Fig. S51**. ^1^H NMR spectrum (600 MHz, DMSO) of **3l**


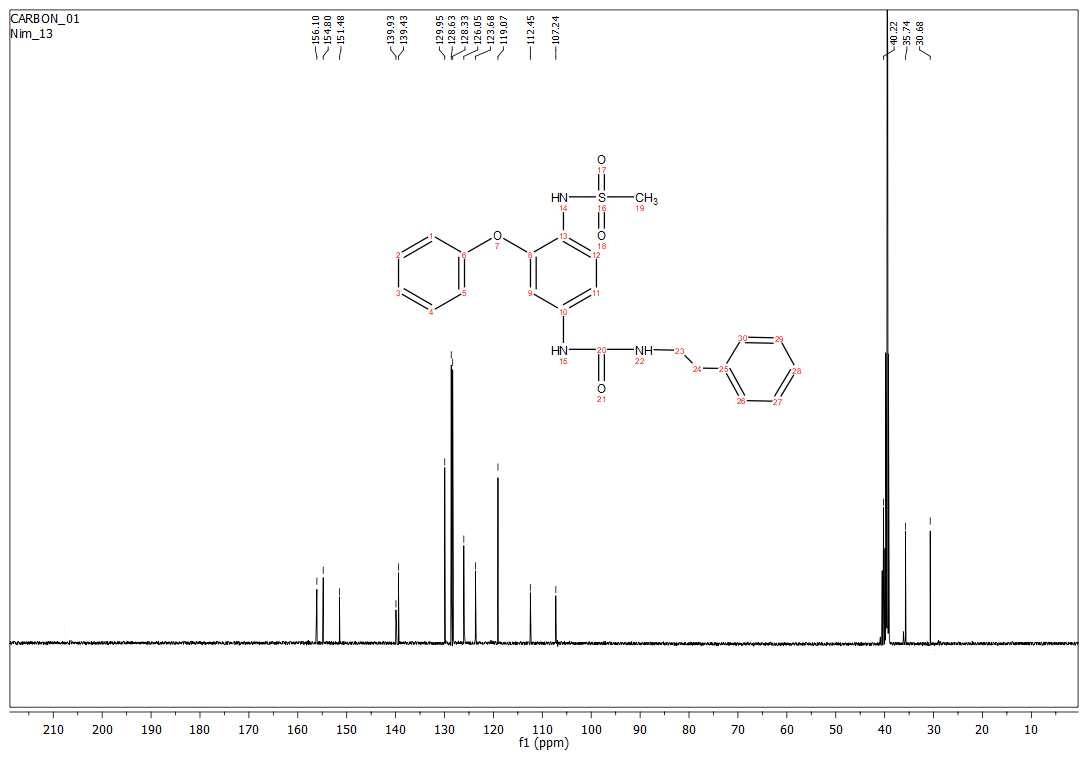


**Fig. S52**. ^13^C NMR spectrum (150 MHz, DMSO) of **3l.**

*Molecular Modeling and Docking Studies*

COX1: The X-ray crystallographic structure of PDB ID:1Q4G, resolution 2.00Å and COX-2: PDB ID:3NT1, resolution 1.73Å were retrieved from the protein data bank (https://www.rcsb.org/). All the co-crystallized ligands, water molecules, and ions were removed, but the HEM group was kept in the COX1 and COX2 proteins. COX-1 and COX-2 proteins were prepared with BIOVIA DS Studio's "Protein Preparation" protocol, and the ligands 3i, 3j, and 3l were prepared with the "Prepare ligands" protocol.

COX-1 and COX-2 protein PDB files were converted to PDBQT format with ADT Tools, and AutoDock VINA was executed one by one. COX-1 and COX-2 protein PDB files were converted to PDBQT format with ADT Tools, and AutoDock VINA was executed one by one.

The detailed procedures for ligand and enzyme preparations were reported elsewhere^22^. The computational docking experiment was carried out for all three compounds, **3i**, **3j**, and **3l**, and the results were presented in Table S1.

| **Proteins** | **MetAP2 Protein** | | **COX1** | | **COX2** | |
| --- | --- | --- | --- | --- | --- | --- |
| **Comp. No** | **Free energy of binding (kcal/mol)** | **Inhibition Constant, Ki**  **(nanoMol)** | **Free energy of binding (kcal/mol)** | **Inhibition Constant, Ki**  **(nanoMol)** | **Free energy of binding (kcal/mol)** | **Inhibition Constant, Ki**  **(nanoMol)** |
| **3i** | -8.0 | 0.737 | -9.0 | 250 | -9.9 | 54 |
| **3j** | -7.9 | 1.50 | -9.4 | 130 | -8.9 | 300 |
| **3l** | -7.8 | 1.73 | -10.4 | 23 | -8.6 | 490 |

**Table S1 .** Calculated molecular docking scores (ΔG, kcal/mol) and inhibition constants (Kᵢ, µM) of compounds **3i, 3j** and **3l.**

| **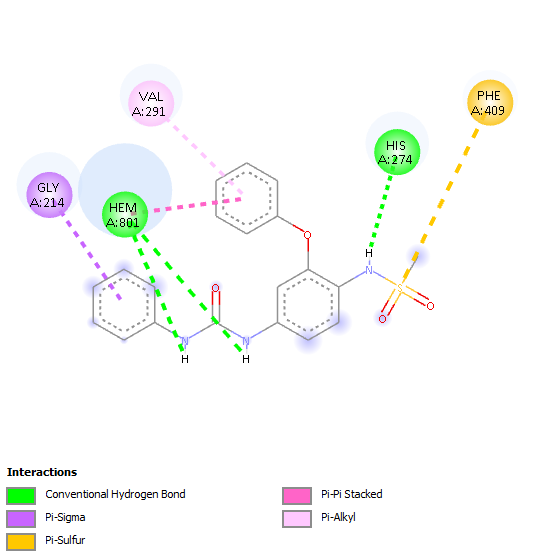**  **COX1-3i** | 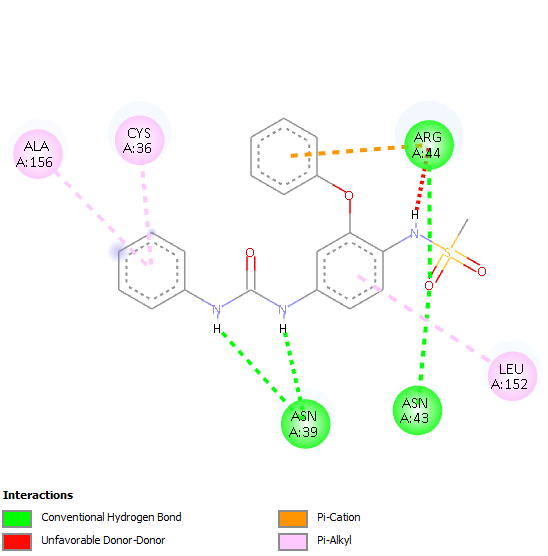  **COX2-3i** |
| --- | --- |
| 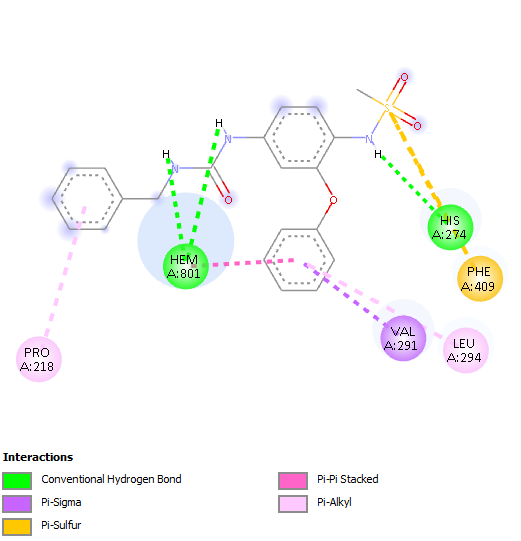  **COX1-3j** | 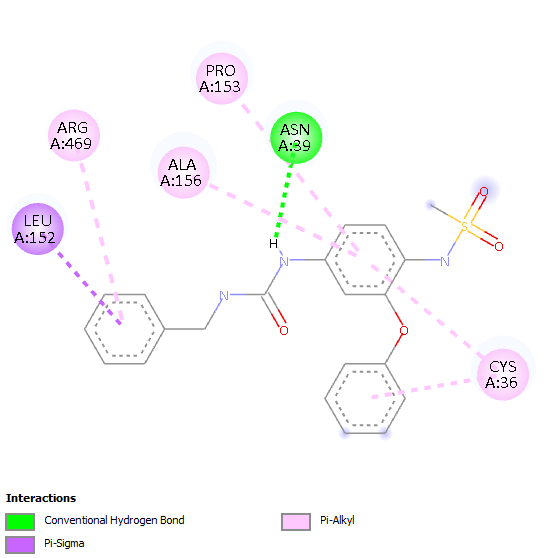  **COX2-3j** |
| 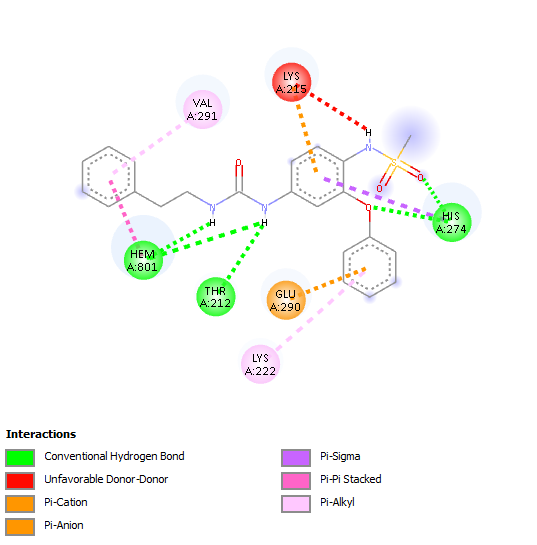  **COX1-3l** | 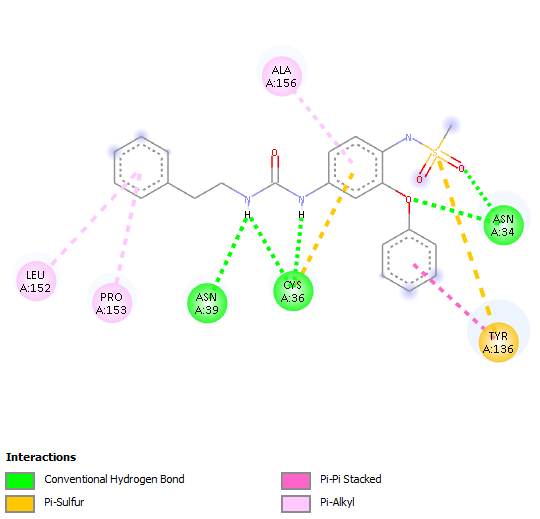  **COX2-3l** |

**Table S2.** The 2D interaction networks of compounds **3i**, **3j**, and **3l** docked into the active sites of COX-1 and COX-2, highlighting key amino acid residue interactions**.**
